# Salicylic Acid as Ionic Liquid Formulation May Have Enhanced Potency to Treat Some Chronic Skin Diseases

**DOI:** 10.3390/molecules27010216

**Published:** 2021-12-30

**Authors:** Joanna Klebeko, Paula Ossowicz-Rupniewska, Ewelina Świątek, Joanna Szachnowska, Ewa Janus, Stefka G. Taneva, Elena Krachmarova, Maya Guncheva

**Affiliations:** 1 Department of Chemical Organic Technology and Polymeric Materials, Faculty of Chemical Technology and Engineering, West Pomeranian University of Technology in Szczecin, Piastów Ave. 42, 71-065 Szczecin, Poland; joanna.klebeko@gmail.com (J.K.); possowicz@zut.edu.pl (P.O.-R.); ewelinaswiatek94@gmail.com (E.Ś.); j.szachnowskaa@gmail.com (J.S.); ejanus@zut.edu.pl (E.J.); 2 Institute of Biophysics and Biomedical Engineering, Bulgarian Academy of Sciences, Acad. G. Bonchev Str. 21, 1113 Sofia, Bulgaria; sgtaneva@gmail.com; 3 Institute of Molecular Biology “Roumen Tsanev”, Bulgarian Academy of Sciences, Acad. G. Bonchev Str., Block 21, 1113 Sofia, Bulgaria; elenakrachmarova@bio21.bas.bg; 4 Institute of Organic Chemistry with Centre of Phytochemistry, Bulgarian Academy of Sciences, Acad. G. Bonchev Bl. 9, 1113 Sofia, Bulgaria

**Keywords:** salicylic acid-based ionic liquids, keratinocytes, fibroblasts, cytotoxicity, anti-inflammatory activity, bovine serum albumin, binding

## Abstract

In recent years, numerous studies have shown that conversion of conventional drugs in ionic liquid (IL) formulation could be a successful strategy to improve their physicochemical properties or suggest a new route of administration. We report the synthesis and detailed characterization of eight salicylic acid-based ILs (SA-ILs) containing cation non-polar or aromatic amino acid esters. Using in vitro assays, we preliminary evaluated the therapeutic potency of the novel SA-ILs. We observed that conversion of the SA into ionic liquids led to a decrease in its cytotoxicity toward NIH/3T3 murine embryo fibroblasts and human HaCaT keratinocytes. It should be mentioned is that all amino acid alkyl ester salicylates [AAOR][SA] inhibit the production of the proinflammatory cytokine IL-6 in LPS-stimulated keratinocytes. Moreover, keratinocytes, pretreated with [PheOMe][SA] and [PheOPr][SA] seem to be protected from LPS-induced inflammation. Finally, the novel compounds exhibit a similar binding affinity to bovine serum albumin (BSA) as the parent SA, suggesting a similar pharmacokinetic profile. These preliminary results indicate that SA-ILs, especially those with [PheOMe], [PheOPr], and [ValOiPr] cation, have the potential to be further investigated as novel topical agents for chronic skin diseases such as psoriasis and acne vulgaris.

## 1. Introduction

The development of innovative drug formulations has been the focus of pharmaceutical research and industry for the last few decades. Novel nanoscale delivery systems are attractive for targeted delivery and aim to increase the drug tissue bioavailability, especially chemotherapeutic drugs [1]. Compared to conventional oral medicines, many modified dosage forms provide sustained, extended, or slow-release or action, which ensure the release of the active substance at optimal therapeutic concentrations and subsequently minimize the toxic effects and allow for the reduction in dosing frequency [2]. Nowadays, more than 40% of the small molecule oral drugs on the market and 90% of the newly synthesized by pharmaceutical companies’ drug candidates are poor water-soluble compounds [3,4]. In addition, many biologically active compounds from aromatic and medicinal plants are also poor water-soluble compounds and not stable, which limits their therapeutic application [5]. The development of novel formulations of oral drugs with enhanced water solubility or proposing alternative routes of administration can be a successful approach to address this problem [4,6]. Another major problem of the pharmaceutical industry is the ability of solid drugs to crystallize into different polymorphic forms. The different polymorphs of one and the same molecule may have different physicochemical properties, including water solubility, bioavailability, tissue distribution, and therapeutic efficacy [7]. Numerous strategies such as pH adjustment, conversion of the drug into salt form, co-crystallization, use of excipients, particle size reduction, lipid-based formulation, and others have been applied to resolve the problems with low water solubility and stability of drugs [8,9].

In recent years, ionic liquids (ILs) have attracted an exponentially increasing interest in biomedicine and pharmacy as biologically active agents, solvents for selective extraction of biologically active compounds, media for co-crystallization, in development of diagnostic tools, etc. [10]. In addition, numerous studies have revealed that ILs based on active pharmaceutical ingredients can be a promising alternative to conventional drugs [11]. ILs are organic salts comprising a bulky asymmetric organic cation and organic or inorganic anion. Their physicochemical properties and pharmaceutical activities can be easily modified by selecting the cation or anion or their modifications [12]. For example, ILs containing cholinium, trimethylammonium, aminoacid ethyl ester, or tertrabutylphosphonium cations and methotrexate anion (antineoplastic agent) showed at least 5000 times higher solubility in simulated body fluids than that of the free methotrexate and are two times more soluble compared to the sodium salt [13]. In addition, it was reported that most of the methotrexate-based ILs exhibited enhanced activity compared to the parent molecule, which is attributed to facilitating penetration of the drug into the membrane of HeLa cells (cervical cancer cells) disrupting its physiological functions, ultimately leading to cell death. Similar effects, namely enhanced cytotoxicity towards tumor cell lines and improved selectivity, were reported for many ILs based on doxorubicin, ampicillin, mitoxantrone, betulinic acid, and many others [14]. ILs also provide a rational platform in the fight against bacterial resistance to antibiotics. For example, Ferraz et al. demonstrated that, while penicillin G and amoxicillin do not have bactericidal or bacteriostatic activity on the resistant Gram-negative *E. coli* strains CTX M9 and CTX M2, as well as the methicillin-resistant *S. aureus* ATCC 43 300, their IL formulations containing 1-ethyl-3methyl-imidazolium, 1-hexadecylpyrrolidinium, or triethylammonium cations display very strong antibacterial activity towards these bacteria [15]. Moreover, in vivo studies have shown that numerous pyrrolidinium, quaternary ammonium bromides, bis(trifluoromethanesulfonyl)imides, or hexafluorophosphates in combination with designed appropriate physicochemical and mechanistic characteristics have potential in dermal and dental tissue engineering, in wound healing, and regeneration due to their antimicrobial and antifouling properties [16]. The IL strategy to improve the drug formulation is investigated extensively also for another widely prescribed group of drugs—non-steroidal anti-inflammatory drugs (NSAIDs). They are used to relieve pain, reduce fever symptoms, and inhibit inflammation [17,18]. Chronic inflammatory diseases require long-term treatment with NSAIDs, which, although relatively safe, have some side effects. They are related to the route of administration (mainly orally) and low solubility [18,19]. In a recently published study, Chantereau et al. proposed novel wound dressings based on cholinium ibuprofenate, cholinium ketoprofenate, and cholinium (*S*)-naproxenate incorporated into bacterial nanocellulose as an innovative method for topical drug application [20]. On the other hand, Parra-Ruiz et al. synthesized dual-functional polymerizable salts containing quaternary ammonium cations linked to NSAIDs, effectively providing a 10-day sustained drug release. The materials exhibited both good antibacterial and anti-inflammatory activity [21]. It has been observed that deep eutectic lidocaine ibuprofen in the composition of IL microemulsion is an efficient transport system for transdermal delivery of artemisinin, an effective antimalarial drug [22]. It should be mentioned that the medicinal application of dual active ionic liquids containing cations with antimicrobial, anesthetic, and anti-inflammatory activity and the s counter ion of NSAID is an innovative approach to optimize drug formulations [23,24]. The two ions may have a synergistic effect or different site interaction mechanism and minimize the number of drugs taken from the patient. NSAIDs are highly hydrophobic molecules with low to moderate solubility in aqueous media. IL formulation of NSAIDs with an appropriate cation may significantly increase the solubility of drugs in water or simulated body fluids. For example, ibuprofenates with short-chain alcohol-functionalized imidazolium, triethylammonium, or cholinium cations showed up to three orders of magnitude and 10 times enhanced water solubility in comparison to that of the ibuprofen in neutral or sodium salt form [25]. Previous studies have shown that ILs based on ketoprofen or naproxen containing cation amino acid esters are not toxic to mouse macrophages RAW264.7 and have a similar binding profile to serum albumin [26,27]. On the other hand, cations such as amino acid alkyl esters, donepezil, *N*-methyl-2-pyrrolidonium, and others enhance NSAIDs’ skin penetration and are the basis of the development of novel formulations for transdermal application [28,29,30,31].

Salicylic acid (SA) is the metabolite of acetylsalicylic acid and is taken in high doses, is considered NSAID, and possesses analgesic, anti-inflammatory, and antirheumatic activities. In contrast, low doses of SA have antiplatelet action [32]. It is also in food conservant, bactericide, and antiseptic. In topical formulations, salicylic acid has keratolytic, comedolytic, and bacteriostatic properties. It is used to treat chronic skin diseases such as psoriasis and acne, characterized by hyperproliferation of keratinocytes and inflammation [33]. The common side effects of dermal agents containing SA are irritation, salicylate poisoning, and the risk of hyperpigmentation [34]. Only a few published studies are focused on the SA-based ILs. However, the results are promising and suggest that this could successfully develop new, safer, and effective SA formulations. For example, 4-aminosalicylate combined with cholinium cation exhibited up to 25-fold higher solubility in water, gastric fluid, and intestinal fluid than SA, suggesting its enhanced bioavailability [35]. On the other hand, salicylates with cation 3-diethylamino-1-propanol or 3-dimethylamino-1-propanol are more effective antioxidants than SA [36]. In a recent study, Moshikur et al. demonstrated that ILs of SA with cation ethyl esters of proline, alanine, or aspartic acid are biocompatible, and their skin penetration is 3 to 6 times more effective than that of sodium salicylate [37].

This study is focused on the synthesis of novel SA-based ILs and preliminary evaluation of their therapeutic potency. We prepared and characterized eight SA-ILs containing cation non-polar or aromatic amino acid esters in detail. l-valine, l-leucine, and l-phenylalanine were selected for the research because they belong to the essential exogenous amino acids and are involved in many processes in the body. l-valine and l-leucine are branched-chain amino acids that enhance energy, increase endurance, and aid in muscle tissue (such as bones, skin, and muscles) recovery and repair. Furthermore, leucine can assist in preventing the breakdown of muscle proteins that sometimes occur after trauma or severe stress. Additionally, l-phenylalanine is used, among others, in the treatment of chronic pain, osteoarthritis, or rheumatoid arthritis. For this reason, the mentioned amino acids were used as conjugates with ibuprofen. Amino acid alkyl esters with an alkyl chain length of 1 to 4 were used in the research because our previous studies on ibuprofen derivatives showed that these chains had the most significant potential [38,39]. In vitro, we evaluated the cytotoxicity and anti-inflammatory activities of the SA-ILs on NIH/3T3 murine embryo fibroblasts and human HaCaT keratinocytes. We also assessed the effect of the cation on SA binding to bovine serum albumin.

## 2. Results and Discussion

### 2.1. Synthesis of the Target Salicylic Acid-Based Ionic Liquids

**Scheme 1:**
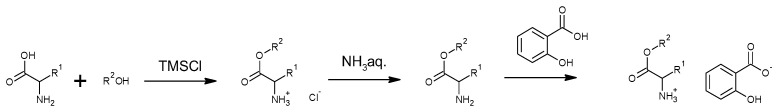
General synthetic procedure for amino acid alkyl ester salicylate, [AAOR][SA].

Eight ILs containing amino acid alkyl ester cations, [AAOR], and the salicylic acid anion [SA] were synthesized by using a slight modification of an established three-step procedure as described in [37,38,39,40] (Scheme 1).

The synthesized compounds used were as follows: l-phenylalanine methyl ester salicylate, [PheOMe][SA]; l-valine ethyl ester salicylate, [ValOEt][SA]; l-leucine ethyl ester salicylate, [LeuOEt][SA]; l-phenylalanine ethyl ester salicylate, [PheOEt][SA]; l-valine propyl ester salicylate, [ValOPr][SA]; l-valine isopropyl ester salicylate [ValOiPr][SA]; l-valine butyl ester salicylate, [ValOBu][SA]; and l-phenylalanine propyl ester salicylate, [PheOPr][SA], which were identified based on spectroscopic methods (ie: ^1^H and ^13^C-NMR, FT-IR, UV-Vis) and elemental analysis. Thus, obtaining the desired structures was confirmed. NMR and FT-IR spectra can be found in the Supplementary Materials in Figures S1–S21.

The melting points (T_m_), thermal stability (T_onset_), and the maximum decomposition temperature (T_max_) are presented in Table 1. TG and DSC curves for [AAOR][SA] are available in Figures S22–S35 (Supplementary Materials). It has been demonstrated that the amino acid type and the alkyl chain’s length and branching influence the stability, solubility, and melting point of the salicylic acid derivatives. These properties can be projected by appropriately selecting the type of amino acid and the alcohol appropriate for esterification. All the compounds obtained had a lower melting point than salicylic acid.

The DSC curves showed two melting points for [PheOMe][SA] and [ValOPr][SA], indicating that these compound’s crystalline forms were obtained. The obtained salicylic acid derivatives showed a lower melting point than the starting acid in all cases. In the case of the following derivatives, [ValOEt][SA], [ValOPr][SA], [ValOBu][SA], [LeuOEt][SA], and [PheOEt][SA], the melting points were lower than 100 °C. For this reason and their ionic structure, they can be included in the group of ionic liquids. Unfortunately, the obtained modifications, due to the too high melting point (they are not liquids at room temperature), will not be able to prevent the phenomenon of polymorphism. The conversion of salicylic acid into salts of amino acid alkyl esters increased the thermal stability of the derivatives obtained. All the derivatives obtained had a higher maximum decomposition temperature (T_max_) than the parent acid. The onset of the thermal degradation was the highest for [PheOEt][SA], while it was the lowest for [ValOEt][SA]. As can be seen, phenylalanine salts had higher thermal stability than the salts of alkyl amino acids (leucine and valine). The length of the alkyl chain of an amino acid also affects its stability. The longer the chain, the higher the thermal stability.

Since [AAOR][SA] are obtained from optically pure amino acids, the specific and molar rotation of all the compounds obtained were determined. These values are presented in Table 1. The value of specific rotation +8.7 for [ValOPr][SA] was lower compared to the +18.6 for [PheOMe][SA]. As can be seen, the value of optical rotation depends on the type of amino acid and the proportion of the amino acid part of the compound.

One of the critical properties affecting a drug’s bioavailability, and hence its effectiveness, is drug solubility. Table 2 shows the results of the solubility in water of salicylic acid and its derivatives expressed in g of compound per dm^3^ and the amount of active compound in g per dm^3^. As can be seen, the solubility clearly depends on the type of amino acid and the length of the alkyl chain. As can be seen, the elongation of the alkyl chain in both the amino acid part and the ester part significantly reduces the water solubility of the compounds. All the phenylalanine derivatives showed a lower solubility than the starting acid. The solubility of l-valine and l-leucine alkyl ester salicylates was markedly higher than salicylic acid, exceeding it by even 3 times higher (based on the concentration of active substance). The highest solubility in water was obtained for [ValO-Et][SA] (12.709 ± 0.013 (g SA·dm^−3^)), while [PheOPr][SA] had the lowest (0.458 ± 0.001 (g SA·dm^−3^)).

The solubility of the obtained compounds in typical solvents was also determined. The results are summarized in Table 3. Solvents were ranked with decreasing value of empirical solvent polarity parameters, E_T(30)_ [41]. It was shown that all tested compounds were insoluble in water and *n*-hexane, while they were soluble in DMSO. They showed differences in solubilities in other solvents. They mostly had solubility, determined according to this methodology, similar to salicylic acid. Improved solubility of obtained derivatives in chloroform was observed compared to salicylic acid.

### 2.2. Cytotoxicity and Anti-Inflammatory Potential of [AAOR][SA]

At first, we assessed in vitro safety of the SA-ILs using 3T3 neutral red uptake assay, a protocol recommended by European Commission as a reliable and sensitive method to predict oral toxicity [42,43]. The estimated half-maximal inhibitory concentration (IC50) for SA and its IL derivatives were in the millimolar range (0.9–4.4 mM). Therefore, the compounds are considered non-cytotoxic [44]. Furthermore, as seen in Table 4, in general, the conversion of SA to a salt having a counter ion AAOR led to an increase of 24 h IC_50_, which makes them safer than the parent NSAID.

Interestingly, [ValOBu][SA] showed the same cytotoxicity as that of the pure SA. Our results are consistent with the observation of Egorova and co-workers on the contribution of SA to the cytotoxicity of three types of SA-ILs toward fibroblast and colon adenocarcinoma cells. Similar to us, they found that the cytotoxicities of the pure SA and its ILs are comparable regardless of whether the NSAID is in their anion, covalently linked to the cation, or involved both in the anion and cation [45].

Next, using MTT assay, we assessed the effect of [AAOR][SA] on the viability of human keratinocytes (HaCaT) cells. The method is based on converting the tetrazolium dye into formazan, and the measured optical density is proportional to the number of viable cells. The estimated 24 h IC_50_ is shown in Table 5. A 48 h exposure of HaCat cells to [ValOR][SA] at a concentration near or less than one-tenth of IC_50_ has a moderate effect on cell proliferation similar to those produced by SA (Table 5). On the contrary, the three [PheOR][SA] are utterly safe for keratinocytes at this concentration.

**Figure 1:**
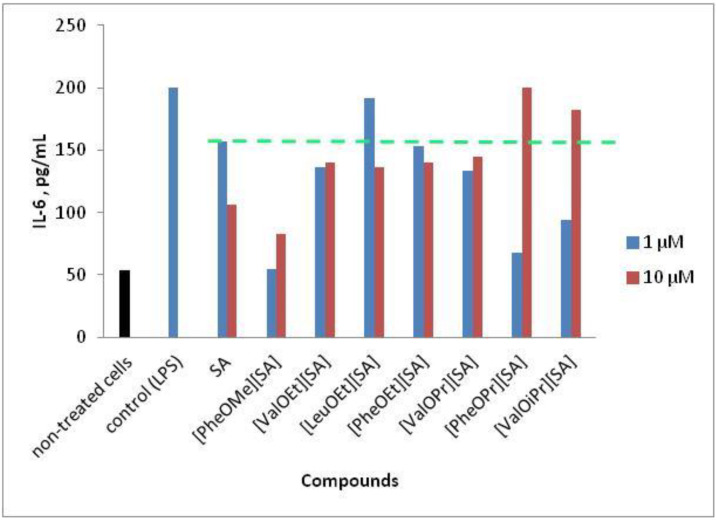
Cytokine levels in the supernatant of LPS-stimulated HaCaT cells were non-treated and treated with 1 or 10 µM [AAOR][SA] or SA.

Interleukin-6 (IL-6) is overexpressed in psoriatic skin and stimulates keratinocyte proliferation [46]. It is predominant in the inflammatory lessons of acne vulgaris and is associated with the pathogenesis of the disease [47]. We assessed the effect of [AAOR][SA] on the protein level of IL-6 in a model of LPS-induced inflammation in human keratinocyte cells (Figure 1). It is noteworthy that [AAOR][SA] were tested at non-toxic concentrations.

In the control experiments, stimulation of HaCaT with LPS resulted in a robust increase in the level of IL-6, which is an indication for induced inflammation. However, compared to control, all tested compounds suppressed the release of IL-6. The effect of [PheOEt][SA], [ValOEt][SA], and [ValOEt][SA] for the two tested concentrations is comparable to those produced by 1 µM SA. Interestingly, HaCaT cells treated with 1µM of [PheOMe][SA] or [PheOPr][SA] seemed to be completely protected from inflammation, and the level of IL-6 estimated for these two samples is equal to those measured for non-stimulated with LPS cells. Interestingly, no effect or negligible inhibition of the IL-6 production was observed for HaCaT cells treated with 10 μM [PheOPr][SA] or [ValOiPr][SA], or 1 μM [LeuOEt][SA].

### 2.3. Energetics of [AAOR][SA]/BSA Binding Interaction assesed by Isothermal Titration Calorimetry

**Figure 2:**
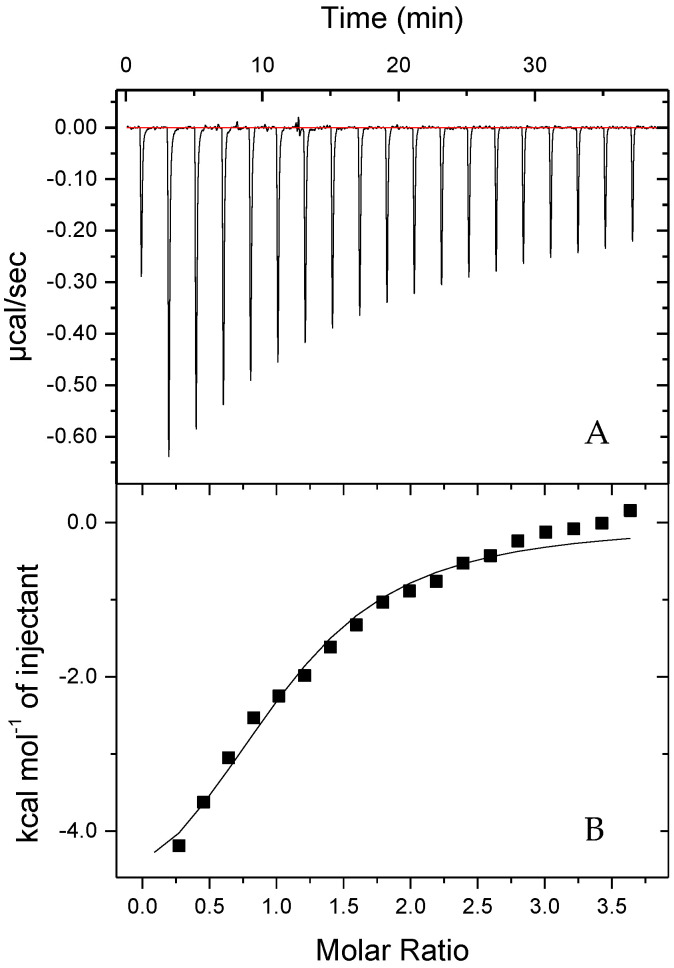
Calorimetric titration of SA (500 µM) with BSA (27 µM) at 25 °C, pH 7.4, raw data (**A**), integrated data, and fit (solid line) of the binding isotherm by the independent binding sites model (**B**).

We assessed the effect of conversion of SA into ILs on its molecular interactions with bovine serum albumin (BSA). Assessment of the drug–serum protein interactions gives essential information for predicting its pharmacokinetics, which impacts drug effectiveness and/or toxicity. Human serum albumin (HSA) is a major transport protein of endogenous and exogenous compounds found in plasma. BSA is highly homologous to HSA and due to its availability is widely used in preliminary binding studies. Figure 2 shows representative isothermal titration calorimetry (ITC) profile of SA/BSA binding interaction. Each peak in the binding isotherm represents a single injection of the ligand into BSA solution (Figure 2A). The binding isotherm refers to the heat of each injection vs. the molar ratio of the ligand and protein (Figure 2B). The data fit the independent binding sites model best and were processed using a standard nonlinear least-squares regression binding model. The respective binding isotherms for SA-ILs/BSA are available in Figures S36–S43 (Supplementary Materials). The thermodynamic parameters for interactions of SA-ILs and BSA obtained from ITC are summarized in Table 5. The results suggest similar interactions of SA-ILs with BSA as compared to those of pure SA. The binding of is SA-ILs to BSA spontaneous process. The negative values of enthalpy (ΔH) and positive values of entropy (ΔS) suggest that the interactions between the protein and ligands are predominantly electrostatic by nature. However, hydrophobic interactions also assist the binding. The binding affinity is in the same range and varies approximately up to 5-fold within the series. Moreover, the obtained values of the dissociation constant (Kd) are in the same range as those reported in the literature for other NSAIDs such as naproxen, ketoprofen, etc., which exclusively bind to site II A of the serum albumins [48,49]. The conversion of SA into ILs did not influence the binding stoichiometry.

**Figure 3:**
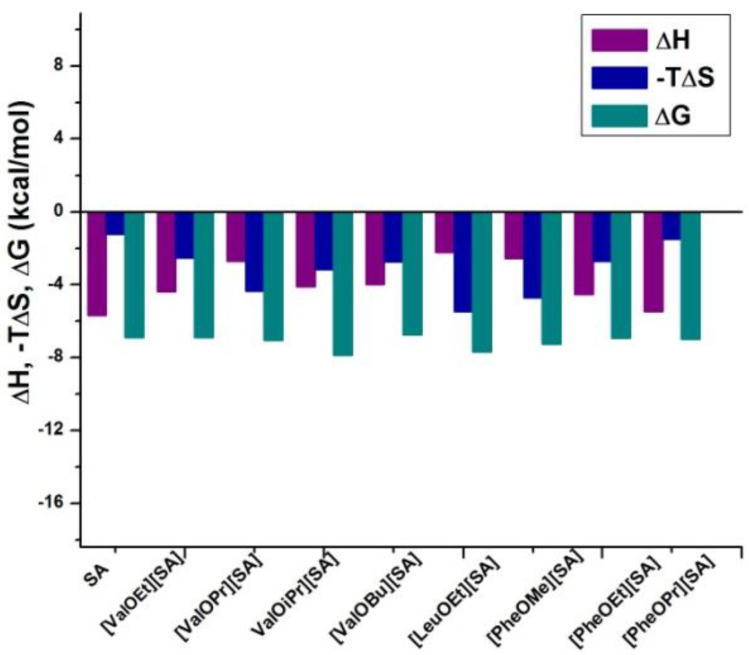
Contribution of the enthalpic (ΔH) and entropic (-TΔS) terms to the Gibbs free energy (ΔG) of the SA and [AAOR][SA] binding to BSA (bar graphs).

The thermodynamic parameters for interactions of SA-ILs and BSA obtained from ITC are summarized in Table 6, and the contribution of the enthalpy (ΔH) and entropy (ΔS) changes to the Gibbs free energy (ΔG) of binding is presented for the studied SA-ILs in Figure 3. ITC data revealed that the binding interaction is dominated by a favorable ΔH, suggesting an electrostatic nature of the interaction that is, however, also assisted by favorable entropic term (−TΔS), indicating a contribution of the hydrophobic effect to the binding affinity (Figure 3).

Although the binding affinity of the studied SA-ILs to BSA is in the same range, the contribution of the enthalpic and the entropic terms to DG differs for the different SA-ILs. DS had the lowest contribution to the [PheOpr][SA] binding to BSA, which is close to the one of pure SA, and the strongest contribution to the binding of [LeuOEt][SA] to BSA, which most differs from the binding energetics of SA to BSA. The conversion of SA into ILs did not influence the binding stoichiometry. Moreover, the obtained values of Kd are in the same range as those reported in the literature for other NSAIDs, such as naproxen, ketoprofen, etc., which exclusively bind to site II A of the serum albumins [48,49].

## 3. Materials and Methods

### 3.1. Materials

Salicylic acid (≥99.0%) and trimethylsilyl chloride (TMSCl) (≥99%) were purchased from Sigma-Aldrich (Geel, Belgium). l-valine, l-leucine (≥99%) and l-phenylalanine (≥99%) were provided from Carl Roth (Karlsruhe, Germany). Methanol and propan-1-ol were high purity and purchased from Chempur (Piekary Ślaskie, Poland). Chloroform, propan-2-ol, and ethyl acetate were high purity and obtained from P. P. H. Stanlab (Lublin, Poland). Ethanol, ammonia solution 25%, *n*-hexane, and toluene, all with high purity, as well as diethyl ether (99.8%), were provided from Avantor (Gliwice, Poland). Deuterated chloroform (CDCl_3_) 99.8 atom %D, containing 1% (*v*/*v*) TMS, was purchased from Eurisotop (Saint-Aubin, France).

NIH/3T3 murine fibroblasts were purchased from American Type Culture Collection (ATCC), while human keratinocytes (HaCaT cells) were from cell line service (CLS) (Eppelheim, Germany).

Dulbecco’s Modified Eagle’s Medium high glucose, with 4500 mg/L glucose, l-glutamine, and sodium bicarbonate, and without sodium pyruvate, which was liquid, sterile-filtered, and suitable for cell culture, as well as trypsin-EDTA solution, 0.25% with phenol red, bovine serum albumin (heat shock fraction, protease-free, free fatty acids free, essentially globulin free), and neutral red and thiazolyl blue tetrazolium bromide were purchased from Sigma. Fetal bovine serum was supplied from Gibco Brazil origin EU approved (Fisher Scientific, Waltham, MA, USA). Penicillin-streptomycin solution, ×100 was provided by EuroClone (Pero, Italy).

The ELISA kit for human IL-6 was obtained from ImunnoTools GmbH (Friesoythe, Germany).

### 3.2. Synthesis of Amino Acid Alkyl Ester Salicylate [AAOR][SA]

[AAOR][SA] were synthesized using a slight modification of an established three-step method [37,38,39]. First, the appropriate amino acid was dispersed into 50 mL of alkyl alcohol at room temperature. Then, two molar equivalents of TMSCl were added to the mixture. The solution was stirred thoroughly at 50 °C for at least 24 h. Then, the excess of TMSCl and alcohol and formed by-products were removed by evaporation at 50 °C under vacuum. The product was purified from the residue of unreacted TMSCl and secondary TMSOH or TMSR was formed by washing with diethyl ether. Then, the product was dissolved in chloroform and filtered under reduced pressure to purify it from unreacted amino acids. The filtrate was distilled off under reduced pressure (50 °C, 10 mbar). The obtained hydrochloride was dried in a vacuum dryer at 50 °C, 5 mbar for 24 h. Then, the obtained amino acid alkyl was added to the small amount of distilled water and neutralized by adding one to three molar equivalents of 25% ammonium hydroxide aqueous solution. The solution was intensively mixed, and then the product was extracted with diethyl ether. The organic layer was dried using anhydrous Na_2_SO_4_ and then concentrated under a vacuum. Finally, obtained in the second step, amino acid alkyl ester (ValOR) was dissolved in chloroform, added into an equimolar amount of salicylic acid, and stirred thoroughly at room temperature for 30 min. Then, the solvent was removed by evaporation under vacuum at 40 °C. The obtained salicylate was dried in a vacuum oven at 50 °C for 24 h.

### 3.3. Identification and Properties of Amino Acid Alkyl Ester Salicylates [AAOR][SA]

For all compounds obtained, the identity was confirmed using ^1^H and ^13^C-NMR, FT-IR, UV-Vis, and elemental analysis. Furthermore, the physicochemical properties such as specific rotation, phase transition temperatures, and solubility were examined.

#### 3.3.1. Nuclear Magnetic Resonance (NMR)

The ^1^H and ^13^C-NMR spectra were recorded in CDCl_3_ on a DPX-400 Avance III HD spectrometer (Bruker, Bremen, Germany) operating at 400.13 MHz (^1^H) and 100.62 MHz (^13^C). TMS was used as an internal standard.

#### 3.3.2. Attenuated Total Reflectance Fourier Transform Infrared Spectroscopy (FTIR-ATR)

Spectra were recorded in FTIR 380 FTIR Spectrometer (Thermo Fisher Scientific Nicolet, Waltham, MA, USA) equipped with attenuated total reflectance (ATR) sampling accessory (diamond plate). Spectra were recorded in transmittance mode from 400 to 4000 cm^−1^, co-adding 16 interferograms at a resolution of 4 cm^−1^.

#### 3.3.3. UV-Vis

UV-Vis spectra were recorded on a Spectroquant^®^ Pharo 300 Spectrophotometer from Merck (Darmstadt, Germany). The solutions were prepared in absolute ethanol of concentration range 10^−4^–10^−5^ M. The measurements were performed in 10 mm quartz cell in the wavelength range 190–400 nm with the accuracy of ±1 nm.

#### 3.3.4. Elemental Analysis

The determination of elemental composition was carried out on Thermo Scientific™ FLASH 2000 CHNS/O Analyzer (Waltham, MA, USA). 2,5-Bis(5-(*tert*-butyl)-2-benzo-oxazol-2-yl)thiophene, l-cysteine, l-methionine, and sulphanilamide were used as standards in CHNS-mode, and acetanilide and benzoic acid were used for calibration in O-mode, respectively. The samples were prepared in the tin (CHNS analysis) or silver (O analysis) crucibles and were weighed with an accuracy of ±0.000001 g.

#### 3.3.5. Thermogravimetric Analysis (TG)

In a corundum crucible, TG was carried out on thermo-microbalance TG 209 F1 Libra (Netzsch, Selb, Germany). Samples between 5 and 10 mg were heated from 25 °C to 1000 °C with a heating rate of 10 °C∙min^−1^, under air atmosphere, with a flow rate of air at 25 cm^3^∙min^−1^ and nitrogen (as protective gas) at 10 cm^3^∙min^−1^.

#### 3.3.6. Differential Scanning Calorimetry (DSC)

Phase transition temperatures were performed using a DSC analysis on the differential calorimeter Q-100 (TA Instruments, New Castle, DE, USA) in a temperature range of 0 to the specified temperature with a heating rate of 10°/min in the nitrogen atmosphere. The specified temperature was the individual temperature for each compound, and it was at least 10 °C lower than the onset decomposition temperature determined from TG analysis. Indium and mercury were used as standards to calibrate the temperature. Heat calibration used indium.

#### 3.3.7. Specific Rotation

The measurement of specific rotation [α]_D_^20^ was performed on AUTOPOL IV Polarimeter from Rudolph Research Analytical (Hackettstown, NJ, USA) at 20 ± 0.1 °C and in 589 nm wavelength. The concentrations of compounds were about 0.005 g cm^−3^ in ethanol as a solvent.

#### 3.3.8. Solubility

The solubility analysis of salicylic acid and its derivatives in water was performed by assessing the concentration of saturated solutions. Then, the obtained saturated solutions were diluted 100 times and analyzed. The analysis was made using high-performance liquid chromatography (HPLC) with a DAD/FLD detector-SHIMADZU Nexera-i LC-2040C 3D High Plus liquid chromatograph (Kyoto, Japan). A mixture of 50% acetonitrile and 50% water was used as the mobile phase. The flow rate was 1 cm^3^·min^−1^. The column temperature was 30 °C. A Luna^®^ Omega 3 µm Polar C18 100 Å column with dimensions of 150 × 4.6 mm was used. The injection volume for these samples was 50 mm^3^. Each measurement was performed in triplicate, and the results were averaged.

The solubility of salicylic acid and its derivatives in conventional organic solvents (polar and non-polar) was evaluated following the modified Vogel method at the temperature of 25 °C. This method classified the compound as soluble, partly soluble, and practically insoluble. For this purpose, dimethyl sulfoxide, ethanol, chloroform, ethyl acetate, diethyl ether, toluene, and *n*-hexane were used as solvents.

#### 3.3.9. Identification of Salicylic Acid Derivatives

[PheOMe][SA]—l-phenylalanine methyl ester salicylate was obtained as a white solid in 89% yield and was identified by ^1^H and ^13^C-NMR, FTIR, and elemental analysis.

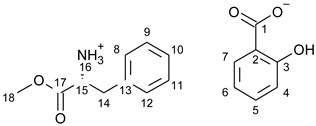



^1^H-NMR (400 MHz, CDC_l3_) δ in ppm: 7.66 (dd, *J*_7,6_ = 7.8, 1.8 Hz, 1H, H7), 7.32–7.27 (m, 1H, H5), 7.18–7.04 (m, 5H, H8, H9, H10, H11, H12), 6.83 (dd, *J*_4,3_ = 8.3, 1.1 Hz, 1H, H4), 6.71 (ddd, *J*_6,5_ = 8.1, 7.3, 1.2 Hz, 1H, H6), 4.09–3.98 (m, 1H, H15), 3.56 (s, 3H, H18), 3.13 (dd, *J*_15,14_ = 14.1, 5.6 Hz, 1H, H14), 3.04 (dd, *J*_15,14_ = 14.1, 7.0 Hz, 1H, H14); ^13^C-NMR (100 MHz, CDCl_3_) δ in ppm: 174.85 (C1), 171.24 (C17), 161.66 (C3), 134.43 (C13), 134.38 (C5), 130.64 (C7), 129.22 (C8, C12), 128.99 (C10), 127.67 (C9, C11), 118.52 (C2), 117.00 (C6), 115.86 (C4), 54.42 (C15), 52.76 (C18), 37.90 (C14); FT-IR ν (ATR): 2950, 2619, 2362, 1745, 1594, 1559, 1497, 1481, 1453, 1384, 1355, 1307, 1238, 1213, 1189, 1156, 1139, 1086, 1030, 996, 948, 921, 893, 862, 814, 756, 746, 702, 666, 536, 454, 429 cm^−1^; Elemental analysis: Calc. (%) for C_17_H_18_NO_5_ (316.329 g/mol) C (64.55), H (5.74), N (4.43), O (25.29), Found: C (64.61), H (5.69), N (4.45), O (25.28); UV-Vis (EtOH): λ_max_ = 210, 236, 304 nm; [α]_D_^20^ = +18.6 (c = 0.511 g/100 cm^3^ EtOH); T_m_ = 94.2 °C.

[ValOEt][SA]–l-valine ethyl ester salicylate was obtained as a white solid in 97% yield and was identified by ^1^H and ^13^C-NMR, FTIR, and elemental analysis.

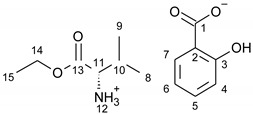



^1^H-NMR (400 MHz, CDCl_3_) δ in ppm: 9.43 (s, 3H, H12), 7.72 (d, *J*_7,6_ = 7.8, 1H, H7), 7.24 (t, *J*_5,4_ = 6.2 Hz, 1H, H5), 6.80 (d, *J*_4,3_ = 7.4 Hz, 1H, H4), 6.68 (t, *J*_6,5_ = 7.4 Hz, 1H, H6), 4.10–3.89 (m, 2H, H14), 3.73 (d, *J*_11,12_ = 4.6 Hz, 1H, H11), 2.29–2.13 (m, 1H, H10), 1.08 (t, *J*_15,14_ = 7.4 Hz, 3H, H15), 0.94 (2d, 6H, H8, H9); ^13^C-NMR (100 MHz, CDCl_3_) δ in ppm: 175.43 (C1), 169.33 (C13), 161.63 (C3), 133.86 (C5), 130.66 (C7), 118.25 (C2), 117.12 (C6), 116.82 (C4), 62.34 (C14), 58.56 (C11), 30.12 (C10), 18.19 (C9), 17.99 (C8), 13.90 (C15); FT-IR ν (ATR): 2972, 2118, 1728, 1594, 1549, 1481, 1448, 1383, 1353, 1306, 1226, 1158, 1141, 1113, 1092, 1074, 1051, 1030, 1010, 951, 859, 831, 804, 752, 702, 665, 565, 534, 451 cm^−1^; Elemental analysis: Calc. (%) for C_14_H_21_NO_4_ (283.324 g/mol) C (59.35), H (7.47), N (4.94), O (28.24), Found: C (60.06), H (7.73), N (4.93), O (28.23); UV-Vis (EtOH): λ_max_ = 210, 232, 300 nm; [α]_D_^20^ = +11.0 (c = 0.510 g/100 cm^3^ EtOH); T_m_ = 59.90 °C.

[LeuOEt][SA]—l-leucine ethyl ester salicylate was obtained as a white solid in 94% yield and was identified by ^1^H and ^13^C-NMR, FTIR, and elemental analysis.

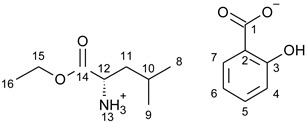



^1^H-NMR (400 MHz, CDCl_3_) δ in ppm: 8.31 (s, 3H, H13), 7.71 (d, *J*_7,6_ = 7.8, 1H, H7), 7.24 (t, *J*_5,4_ = 8.3 Hz, 1H, H5), 6.81 (d, *J*_4,3_ = 8.2 Hz, 1H, H4), 6.67 (t, *J*_6,5_ = 7.4 Hz, 1H, H6), 4.01–3.88 (m, 2H, H15), 3.78 (d, *J*_12,13_ = 7.2 Hz, 1H, H12), 1.77–1.67 (m, 1H, H10), 1.60 (t, *J*_11,10_ = 7.2 Hz, 2H, H11), 1.05 (t, *J*_16,15_ = 7.1 Hz, 3H, H16), 0.76 (2d, 6H, H8, H9); ^13^C-NMR (100 MHz, CDCl_3_) δ in ppm: 175.78 (C1), 170.52 (C14), 161.67 (C3), 133.62 (C5), 130.55 (C7), 118.06 (C2), 117.87 (C6), 116.81 (C4), 62.25 (C15), 51.69 (C12), 40.33 (C11), 24.36 (C10), 22.15 (C9), 21.87 (C8), 13.79 (C16); FT-IR ν (ATR): 2958, 2935, 2871, 2629, 2349, 2170, 1736, 1618, 1595, 1554, 1483, 1453, 1385, 1371, 1353, 1308, 1253, 1227, 1187, 1158, 1139, 1093, 1055, 1030, 1018, 995, 949, 861, 826, 806, 754, 704, 666, 455 cm^−1^; Elemental analysis: Calc. (%) for C_15_H_22_NO_5_ (229.339 g/mol) C (60.80), H (7.48), N (4.73), O (27.00), Found: C (60.85), H (7.34), N (4.72), O (26.94); UV-Vis (EtOH): λ_max_ = 212, 232, 300 nm; [α]_D_^20^ = +13.5 (c = 0.508 g/100 cm^3^ EtOH); T_m_ = 68.2 °C.

[PheOEt][SA]—l-phenylalanine ethyl ester salicylate was obtained as a white solid in 94% yield and was identified by ^1^H and ^13^C-NMR, FTIR, and elemental analysis.

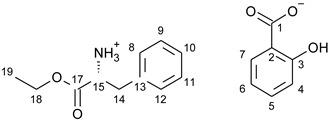



^1^H-NMR (400 MHz, CDCl_3_) δ in ppm: 7.64 (dd, *J*_7,6_ = 7.8, 1.8 Hz, 1H, H7), 7.29–7.16 (m, 1H, H5), 7.15–7.01 (m, 5H, H8, H9, H10, H11, H12), 6.81 (dd, *J*_4,3_ = 8.2, 1.1 Hz, 1H, H4), 6.70–6.64 (m, 1H, H6), 4.03 (t, *J*_14,15_ = 6.5 Hz, 1H, H15), 3.94 (qd, *J*_19,18_ = 7.2, 5.1 Hz, 2H, H18), 3.08 (dd, *J*_15,14_ = 6.5, 3.3 Hz, 2H, H14), 1.02 (t, *J* = 7.1 Hz, 3H, H19); ^13^C-NMR (100 MHz, CDCl_3_) δ in ppm: 175.17 (C1), 170.24 (C17), 161.64 (C3), 134.22 (C13), 134.00 (C5), 130.64 (C7), 129.26 (C8, C12), 128.89 (C10), 127.62 (C9, C11), 118.32 (C2), 116.87 (C6), 116.77 (C4), 62.24 (C18), 54.40 (C14), 37.59 (C14), 13.82 (C19); FT-IR ν (ATR): 2950, 2619, 2363, 1744, 1659, 1594, 1560, 1497, 1481, 1453, 1384, 1355, 1307, 1238, 1213, 1189, 1156, 1139, 1086, 1061, 1030, 996, 948, 921, 893, 862, 814, 756, 746, 702, 666, 638, 567, 536, 480, 454, 430 cm^−1^; Elemental analysis: Calc. (%) for C_18_H_20_NO_5_ (330.356 g/mol) C (65.44), H (6.10), N (4.24), O (24.22), Found: C (65.48), H (6.15), N (4.18), O (24.25); UV-Vis (EtOH): λ_max_ = 210, 232, 300 nm; [α]_D_^20^ = +16.8 (c = 0.519 g/100 cm^3^ EtOH); T_m_ = 98.4 °C.

[ValOPr][SA]—l-valine propyl ester salicylate was obtained as a white solid in 98% yield and was identified by ^1^H and ^13^C-NMR, FTIR, and elemental analysis.

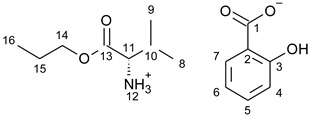



^1^H-NMR (400 MHz, CDCl_3_) δ in ppm: 7.79 (d, *J*_7,6_ = 7.8, 1H, H7), 7.30 (t, *J*_5,4_ = 8.6 Hz, 1H, H5), 6.87 (d, *J*_4,3_ = 8.3 Hz, 1H, H4), 6.75 (t, *J*_6,5_ = 7.6 Hz, 1H H6), 4.06–3.90 (m, 2H, H14), 3.75 (d, *J*_11,12_ = 4.6 Hz, 1H, H11), 2.31–2.17 (m, 1H, H10), 1.61–1.50 (m, 2H, H15), 1.01 (2d, 6H, H8, H9), 0.86 (t, *J*_15,16_ = 7.4 Hz, 3H, H16); ^13^C-NMR (100 MHz, CDCl_3_) δ in ppm: 175.53 (C1), 170.33 (C13), 161.64 (C3), 133.59 (C5), 130.65 (C7), 118.10 (C2), 117.61 (C6), 116.74 (C4), 67.67(C14), 58.72 (C11), 30.39 (C10), 21.69 (C15), 18.13 (C9), 18.08 (C8), 10.24 (C16); FT-IR ν (ATR): 2970, 2165, 2118, 1737, 1593, 1566, 1513, 1480, 1453, 1402, 1380, 1349, 1306, 1289, 1250, 1217, 1173, 1156, 1143, 1110, 1072, 1057, 1030, 960, 929, 862, 828, 804, 753, 706, 665, 567, 532, 452 cm^−1^; Elemental analysis: Calc. (%) for C_15_H_23_NO_4_ (297.350 g/mol) C (60.59), H (7.80), N (4.71), O (26.90), Found: C (60.47), H (7.82), N (4.71), O (26.91); UV-Vis (EtOH): λ_max_ = 210, 232, 300 nm; [α]_D_^20^ = +8.7 (c = 0.566 g/100 cm^3^ EtOH); T_m_ = 79.7 °C.

[PheOPr][SA]—l-phenylalanine propyl ester salicylate was obtained as a white solid in 90% yield and was identified by ^1^H and ^13^C-NMR, FTIR, and elemental analysis.

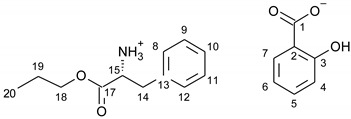



^1^H NMR (400 MHz, CDCl_3_) δ in ppm: 7.73 (dd, J_7,6_ = 7.8, 1.8 Hz, 1H, H7), 7.36–7.31 (m, 1H, H5), 7.23–7.11 (m, 5H, H8, H9, H10, H11, H12), 6.89 (dd, J_4,3_ = 8.3, 1.1 Hz, 1H, H4), 6.75 (ddd, J_6,5(7)_ = 8.1, 7.3, 1.2 Hz, 1H, H6), 4.13 (t, J_14,15_ = 6.5 Hz, 1H, H15), 3.91 (td, J_19,18_ = 6.8, 2.3 Hz, 2H, H18), 3.16 (dd, J_15,14_ = 6.5, 3.1 Hz, 2H, H14), 1.55–1.42 (m, 2H, H19), 0.78 (t, J = 7.4 Hz, 3H, H20); ^13^C NMR (100 MHz, CDCl_3_) δ in ppm: 175.16 (C1), 170.30 (C17), 161.62 (C3), 134.18 (C13), 133.94 (C5), 130.64 (C7), 129.22 (C8, C12), 128.89 (C10), 127.61 (C9, C11), 118.29 (C2), 116.84 (C6), 116.79 (C4), 67.76 (C18), 54.36 (C14), 37.55 (C14), 21.56 (C19), 10.13 (C20); FT-IR ν (ATR): 3029, 2970, 2940, 2880, 2625, 1740, 1626, 1660, 1595, 1559, 1496, 1482, 1454, 1386, 1353, 1307, 1255, 1230, 1157, 1140, 1085, 862, 818, 754, 702, 666, 458 cm^−1^; Elemental analysis: Calc. (%) for C_18_H_20_NO_5_ (344.382 g/mol) C (66.26), H (6.44), N (4.07), O (23.23), Found: C (66.27), H (6.43), N (4.08), O (23.24); UV-Vis (EtOH): λ_max_ = 209, 231, 301 nm; [α]_D_^20^ = +9.0 (c = 0.525 g/100 cm^3^ EtOH); T_m_ = 101.8 °C.

[ValOiPr][SA]—l-valine isopropyl ester salicylate was obtained as a white solid in 95% yield and was identified by ^1^H and ^13^C-NMR, FTIR, and elemental analysis.

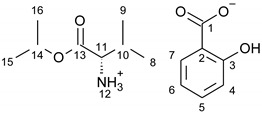



^1^H-NMR (400 MHz, CDCl_3_) δ in ppm: 8.10 (s, 3H, H12), 7.80 (d, *J*_7,6_ = 7.8 Hz, 1H), 7.30 (t, *J*_5,4_ = 6.2, Hz, 1H, H5), 6.87 (d, *J*_4,3_ = 7.2 Hz, 1H, H4), 6.75 (t, *J*_6,5_ = 7.3 Hz, 1H, H6), 5.04–4.90 (m, 1H, H14), 3.76 (d, *J*_11,12_ = 4.3 Hz, 1H, H11), 2.41–2.15 (m, 1H, H10), 1.21 (d, *J*_9_,_10_ = 6.2 Hz, 3H, H9), 1.15 (d, *J*_8,10_ = 6.2 Hz, 3H, H8), 1.02 (2d, 6H, H15, H16); ^13^C-NMR (100 MHz, CDCl_3_) δ in ppm: 175.40 (C1), 169.43 (C13), 161.64 (C3), 133.67 (C5), 130.69 (C7), 118.16 (C2), 117.39 (C6), 116.75 (C4), 70.49 (C14), 58.52 (C11), 30.22 (C10), 21.59 (C15), 21.46 (C16), 18.02 (C9), 17.96 (C8); FT-IR ν (ATR): 2970, 2359, 2193, 1737, 1593, 1566, 1513, 1480, 1454, 1402, 1380, 1349, 1306, 1289, 1250, 1217, 1173, 1156, 1143, 1110, 1073, 1057, 1030, 959, 929, 862, 829, 804, 753, 706, 664, 566, 532, 452 cm^−1^; Elemental analysis: Calc. (%) for C_15_H_23_NO_4_ (297.350 g/mol) C (60.59), H (7.80), N (4.71), O (26.90), Found: C (60.47), H (7.79), N (4.71), O (26.94); UV-Vis (EtOH): λ_max_ = 210, 232, 300 nm; [α]_D_^20^ = +12.5 (c = 0.577 g/100 cm^3^ EtOH); T_m_ = 102.6 °C.

[ValOBu][SA]—l-valine butyl ester salicylate was obtained as a white solid in 93% yield and was identified by ^1^H and ^13^C-NMR, FTIR, and elemental analysis.

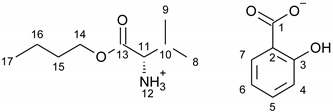



^1^H-NMR (400 MHz, CDCl_3_) δ in ppm: 8.32 (s, 3H, H12), 7.80 (d, *J*_7,6_ = 7.8 Hz, 1H, H7), 7.31 (t, *J*_5,4_ = 1H, H5), 6.88 (d, *J*_4,3_ = 8.2, Hz, 1H, H4), 6.76 (t, *J*_6,5_ = 7.2 Hz, 1H, H6), 4.18–3.92 (m, 2H, H14), 3.79 (d, *J*_11,12_ = 4.6 Hz, 1H, H11), 2.33–2.21 (m, 1H), 1.59–1.43 (m, 2H, H15), 1.35–1.21 (m, 2H, H16), 1.02 (2d, 6H, H8, H9), 0.86 (t, *J*_17,16_ = 7.4 Hz, 3H, H17); ^13^C-NMR (100 MHz, CDCl_3_) δ in ppm: 175.39 (C1), 169.93 (C13), 161.65 (C3), 133.80 (C5), 130.68 (C7), 118.22 (C2), 117.15 (C6), 116.81 (C4), 66.14 (C14), 58.59 (C11), 30.28 (C10), 30.23 (C15), 18.97 (C16), 18.09 (C9), 18.05 (C8), 13.55 (C17); FT-IR ν (ATR): 2980, 2573, 2119, 1919, 1728, 1659, 1594, 1556, 1481, 1450, 1381, 1350, 1308, 1293, 1254, 1235, 1176, 1156, 1135, 1114, 1070, 1031, 967, 946, 886, 860, 825, 805, 751, 703, 665, 566, 535, 455, 430 cm^−1^; Elemental analysis: Calc. (%) for C_15_H_23_NO_4_ (311.377 g/mol) C (61.72), H (8.09), N (4.50), O (25.69), Found: C (61.46), H (8.14), N (4.50), O (25.73); UV-Vis (EtOH): λ_max_ = 208, 232, 300 nm; [α]_D_^2^^0^ = +9.6 (c = 0.512 g/100 cm^3^ EtOH); T_m_ = 70.7 °C.

### 3.4. Cell Culture

Murine 3T3 fibroblasts and human keratinocytes (HaCaT) were maintained in Dulbecco’s Modified Eagle’s Medium (DMEM) high glucose with 10% fetal bovine serum and 1% antibiotics (10,000 μg/mL streptomycin and 10,000 units/mL penicillin) at 37 °C with 5% CO2. For the cytotoxicity assays, 3T3 cells (2 × 104) or HaCaT (2 × 104) were grown as a monolayer (70–80% confluence) at DMEM high glucose with 10% FBS. Then, cells were incubated for 24/48 h with various concentrations of SA or [AAOR][SA] at 0 to 20 mM. Finally, control experiments with non-treated cells were performed.

### 3.5. Neutral Red Uptake Assay (NRU)

The effect of the compounds on the viability of murine 3T3 fibroblasts was estimated using NRU assay. After the incubation time (24 h) cells, media were removed, then to each well we added 100 µL of medium containing neutral red (50 µg/mL). After a 3 h incubation at 37 °C, the supernatant was withdrawn, and cells were fixed with 100 µL of a solution containing glacial acid-ethanol-water (1:50:49, *v*/*v*/*v*). The plates were shaken for 10 min at room temperature. Then, the absorption at 540 nm was measured using a microplate reader (Biosan). The results were calculated for each sample concentration as a percentage of medium control. The values are presented as mean ± standard deviation of three independent experiments.

### 3.6. MTT Assay

We applied an assay based on the reduction of methyl thiazol tetrazolium bromide (MTT) to assess the effect of the compounds on the viability of HaCaT cells. Briefly, after treatment with SA or [AAOR][SA] for 24 or 48 h, the media were removed. Then cells were incubated with 100 µL of media containing MTT with a final concentration 50 µg/100 µL for 3 h at 37 °C. After that, the cells were lysed with a solution of DMSO:ethanol (1:1 *v*/*v*). The absorbance of the reduced formazan dye product was read at 560 nm in a microplate reader (Biosan). The results were calculated for each sample concentration as a percentage of medium control. The values are presented as mean ± standard deviation of three independent experiments.

### 3.7. Quantitative Estimation of IL-6 Cytokine

HaCaT cells were seeded in a 24-well plate at a density of 5 × 104 cells/well and maintained in DMEM high glucose with 10% FBS until an 80% confluent monolayer was reached. Then, media were changed with serum-free media, and cells were incubated with 1 or 10 µM SA or [AAOR][SA] for 12 h at 37 °C. Next, in each well we added 0.5 µM of LPSm and plates were incubated for another 12 h at 37 °C. Control experiments with LPS-treated cells only were conducted. Controls with cells treated with neither compounds nor LPS were also performed. After that, the cell culture supernatant was collected, and the IL-6 cytokine was measured using an ELISA kit.

### 3.8. Isothermal Titration Calorimetry (ITC)

ITC experiments were carried out using a MicroCal ITC200 (Malvern) with an operating cell volume of 300 μL. The protein solution (27 µM) was placed in the calorimeter cell and titrated with ligands in 20 mM phosphate saline buffer, pH 7.4. Injections of 2 µL were performed under a constant stirring speed of 250 rpm, and at 120 s intervals (the first injection of 1 µL was not considered in the analysis). All experiments were performed at 25 °C.

The heat of the dilution of SA or [AAOR][SA] into a buffer was used to correct the data. The binding isotherms were analyzed with a model of independent binding sites using the Origin software 7.0 provided by MicroCal.

## 4. Conclusions

We synthesized and characterized eight novel salicylic acid-based ionic liquids in detail with respect to their structure and physicochemical properties. We observed that conversion of the SA into ionic liquids led to a decrease in its cytotoxicity toward murine fibroblasts and human keratinocytes. Noteworthy to mention is that all [AAOR][SA] inhibit the production of the proinflammatory cytokine IL-6 in LPS-stimulated keratinocytes. Moreover, keratinocytes pretreated with [PheOMe][SA] and [PheOPr][SA] seem to be protected from LPS-induced inflammation. Finally, the novel compounds exhibit a similar binding affinity to BSA as the parent SA, suggesting a similar pharmacokinetic profile. These preliminary results indicate that [AAOR][SA], especially those with [PheOMe], [PheOPr], and [ValOiPr] cations, have the potential to be further investigated as novel topical agents for chronic skin diseases such as psoriasis and acne vulgaris.

## Figures and Tables

**Table 1:** The properties of salicylic acid and amino acids alkyl esters salicylates.

No.	Compound	Color	T_m_/°C	T_onset_/°C	T_max_/°C	[α]_λ_^T^
1	SA	white	135.0	171.3	199.1	0.0
2	[ValOEt][SA]	white	57.5	157.6	187.5	+11.0
3	[ValOiPr][SA]	white	102.6	163.1	198.7	+12.5
4	[ValOPr][SA]	white	67.6/79.7	177.9	208.7	+8.7
5	[ValOBu][SA]	white	68.8	171.8	205.7	+9.6
6	[LeuOEt][SA]	white	68.2	159.2	194.0	+13.5
7	[PheOMe][SA]	white	94.4/120.6	170.5	205.7	+18.6
8	[PheOEt][SA]	white	98.4	181.1	212.4	+16.8
9	[PheOPr][SA]	white	101.8	185.0	212.3	+9.0

T_m_—melting point, T_onset_—the onset of the thermal degradation, T_max_—maximum decomposition temperature, [α]^λ^_T_—specific rotation.

**Table 2:** The solubility of salicylic acid and l-proline alkyl ester naproxenates in water at 25 °C.

Compound	Solubility in Water at 25 °C	
	g·dm^−^^3^	g SA·dm^−3^
SA	3.852 ± 0.004	3.852 ± 0.004
[ValOEt][SA]	26.070 ± 0.026	12.709 ± 0.013
[ValOiPr][SA]	18.458 ± 0.018	8.574 ± 0.008
[ValOPr][SA]	21.789 ± 0.024	10.121 ± 0.011
[ValOBu][SA]	14.490 ± 0.014	6.427 ± 0.006
[LeuOEt][SA]	14.377 ± 0.014	8.659 ± 0.008
[PheOMe][SA]	2.307 ± 0.002	1.007 ± 0.001
[PheOEt][SA]	9.732 ± 0.010	4.069 ± 0.004
[PheOPr][SA]	1.143 ± 0.001	0.458 ± 0.001

**Table 3:** Solubility in typical solvents of salicylic acid and amino acids alkyl esters salicylates at 25 °C.

No.	Compound	Water (63.1)	Ethanol (51.9)	DMSO (45.1)	Chloroform (39.1)	Ethyl Acetate (38.1)	Diethyl ether (34.5)	Toluene (33.9)	*n*-Hexane (31.0)
1	SA	+/−	+	+	+/−	+	+	+/−	−
2	[ValOEt][SA]	+/−	+	+	+	+	+	+	−
3	[ValOiPr][SA]	+/−	+	+	+	+/−	+/−	+	−
4	[ValOPr][SA]	+/−	+	+	+	+	+	+	−
5	[ValOBu][SA]	+/−	+/−	+	+	+	+	+	−
6	[LeuOEt][SA]	+/−	+/−	+	+	+	+	+	−
7	[PheOMe][SA]	−	+/−	+	+	+/−	+	−	−
8	[PheOEt][SA]	−	+/−	+	+	+/−	+	−	−
9	[PheOPr][SA]	−	+/−	+	+	+/−	+	−	−

The E_T(30)_ values of the solvents are given in parentheses; “+”: soluble >100 mg·cm^−3^; “+/−”: partially soluble 33–100 mg·cm^−3^; “−”: insoluble < 33 mg·cm^−3^ [40].

**Table 4:** Cytotoxicity of the studied compounds towards fibroblasts (NIH/3T3 cells).

No.	Compound	IC_50,_ μg/mL (in mM)
1	[PheOMe][SA]	991 ± 10 (≈3.2)
2	[ValOEt][SA]	700 ± 8 (≈2.5)
3	[LeuOEt][SA]	894 ± 12 (≈3.0)
4	[PheOEt][SA]	1062 ± 15 (≈3.2)
5	[ValOPr][SA]	1310 ± 17 (≈4.4)
6	[PheOPr][SA]	662 ± 9 (≈1.9)
7	[ValOiPr][SA]	658 ± 7 (≈2.2)
8	[ValOBu][SA]	309 ± 9 (≈1.0)
9	SA	300 ± 6 (≈2.2)

Values are means ± standard deviation from three consecutive experiments.

**Table 5:** Cytotoxicity of the tested compounds toward human keratinocytes (HaCaT cells).

No.	Compound	IC_50,_ mg/mL (in mM)	Viability of HaCaT after 48 h Treatment with 0.4 mM IL or SA
1	[PheOMe][SA]	1.3 ± 0.20(≈4.1)	98 ± 3
2	[ValOEt][SA]	0.6 ± 0.01 (≈2.1)	74 ± 2
3	[LeuOEt][SA]	0.8 ± 0.12 (≈2.1)	70 ± 3
4	[PheOEt][SA]	2.2 ± 0.15 (≈6.6)	105 ± 4
5	[ValOPr][SA]	0.5 ± 0.05 (≈1.8)	74 ± 2
6	[PheOPr][SA]	0.65 ± 0.1(≈1.9)	93 ± 4
7	[ValOiPr][SA]	0.9 ± 0.05 (≈3.1)	66 ± 2
8	[ValOBu][SA]	0.6 ± 0.2 (≈1.9)	74 ± 1
9	SA	0.2 ± 0.1 (≈1.6)	71 ± 3

Values are means ± standard deviation from three consecutive experiments.

**Table 6:** Thermodynamic binding parameters for the binding of SA and [AAOR][SA] with BSA in PBS buffer (pH 7.4) at 298.15 K.

BSA/IL Complex	Binding Sites (n)	K_a_ (×10^5^ M)	K_d_ (×10^−6^ M)	ΔH (Kcal mol^−1^)	ΔS (cal mol^−1^ K^−1^)	ΔG (kcal/mol)
BSA/SA	1.04	1.17	8.55	−5.68	4.1	−6.90
BSA/[ValOEt][SA]	1.14	1.13	8.77	−4.38	8.5	−6.90
BSA/[ValOPr][SA]	1.40	1.49	6.7	−2.72	14.6	−7.07
BSA/[ValOiPr][SA]	1.03	2.12	4.72	−4.1	10.6	−7.88
BSA/[ValOBu][SA]	1.24	0.89	11.1	−4.0	9.2	−6.75
BSA/[LeuOEt][SA]	1.05	4.43	2.26	−2.23	18.3	−7.69
BSA/[PheOMe][SA]	1.20	2.11	4.74	−2.56	15.8	−7.27
BSA/[PheOEt][SA]	1.06	1.23	8.13	−4.53	9.1	−6.94
BSA/[PheOPr][SA]	0.85	1.33	7.52	−5.48	5.1	−6.99

Values of each parameter listed in the table are average in two to three independent measurements.

## Data Availability

The data presented in this study are available on request from the corresponding author.

## Supplementary Materials

Figure S1. 1H NMR spectra of [PheOMe][SA]; Figure S2. 13C NMR spectra of [PheOMe][SA]; Figure S3. 1H NMR spectra of [ValOEt][SA]; Figure S4. 13C NMR spectra of [ValOEt][SA]; Figure S5. 1H NMR spectra of [LeuOEt][SA]; Figure S6. 13C NMR spectra of [LeuOEt][SA]; Figure S7. 1H NMR spectra of [PheOEt][SA]; Figure S8. 13C NMR spectra of [PheOEt][SA]; Figure S9. 1H NMR spectra of [ValOPr][SA]; Figure S10. 13C NMR spectra of [ValOPr][SA]; Figure S11. 1H NMR spectra of [PheOPr][SA]; Figure S12. 13C NMR spectra of [PheOPr][SA]; Figure S13. 1H NMR spectra of [ValOiPr][SA]; Figure S14. 13C NMR spectra of [ValOiPr][SA]; Figure S15. 1H NMR spectra of [ValOBu][SA]; Figure S16. 13C NMR spectra of [ValOBu][SA]; Figure S17. FT-IR spectra of [PheOMe][SA]; Figure S18. FT-IR spectra of [ValOEt][SA]; Figure S19. FT-IR spectra of [LeuOEt][SA]; Figure S20. FT-IR spectra of [PheOEt][SA]; Figure S21. FT-IR spectra of [ValOPr][SA]; Figure S22. FT-IR spectra of [PheOPr][SA]; Figure S23. FT-IR spectra of [ValOiPr][SA]; Figure S24. FT-IR spectra of [ValOBu][SA]; Figure S25. UV-Vis spectra of [PheOMe][SA]; Figure S26. UV-Vis spectra of [ValOEt][SA]; Figure S27. UV-Vis spectra of [LeuOEt][SA]; Figure S28. UV-Vis spectra of [PheOEt][SA]; Figure S29. UV-Vis spectra of [ValOPr][SA]; Figure S30. UV-Vis spectra of [PheOPr][SA]; Figure S31. UV-Vis spectra of [ValOiPr][SA]; Figure S32. UV-Vis spectra of [ValOBu][SA]; Figure S33. The TG, DTG, and c-DTA curves of [PheOMe][SA]; Figure S34. The TG, DTG, and c-DTA curves of [ValOEt][SA]; Figure S35. The TG, DTG, and c-DTA curves of [LeuOEt][SA]; Figure S36. The TG, DTG, and c-DTA curves of [PheOEt][SA]; Figure S37. The TG, DTG, and c-DTA curves of [ValOPr][SA]; Figure S38. The TG, DTG, and c-DTA curves of [PheOPr][SA]; Figure S39. The TG, DTG, and c-DTA curves of [ValOiPr][SA]; Figure S40. The TG, DTG, and c-DTA curves of [ValOBu][SA]; Figure S41. The DSC curves of [PheOMe][SA]; Figure S42. The DSC curves of [ValOEt][SA]; Figure S43. The DSC curves of [LeuOEt][SA]; Figure S44. The DSC curves of [PheOEt][SA]; Figure S45. The DSC curves of [ValOPr][SA]; Figure S46. The DSC curves of [PheOPr][SA]; Figure S47. The DSC curves of [ValOiPr][SA]; Figure S48. The DSC curves of [ValOBu][SA]; Figure S49. Raw data of titration of 350 µM [LeuOEt][SA] with 27 µM at 25 °C (upper panel). Integrated heat profile of calorimetric titration shown in upper panel (lower panel); Figure S50. Raw data of titration of 500 µM [PheOPr][SA] with 27 µM at 25 °C (upper panel). Integrated heat profile of calorimetric titration shown in upper panel (lower panel). Figure S51. Raw data of titration of 500 µM [PheOPr][SA] with 27 µM at 25 °C (upper panel). Integrated heat profile of calorimetric titration shown in upper panel (lower panel). The solid lines represent the best fit to a single binding site model. Figure S52. Raw data of titration of 350 µM [ValOiPr][SA] with 27 µM at 25 °C (upper panel). Integrated heat profile of calorimetric titration shown in upper panel (lower panel); Figure S53. Raw data of titration of 500 µM [ValOBu][SA] with 27 µM at 25 °C (upper panel). Integrated heat profile of calorimetric titration shown in upper panel (lower panel); Figure S54. Raw data of titration of 500 µM [ValOPr][SA] with 27 µM at 25 °C (upper panel). Integrated heat profile of calorimetric titration shown in upper panel (lower panel); Figure S55. Raw data of titration of 500 µM [ValOEt][SA] with 27 µM at 25 °C (upper panel). Integrated heat profile of calorimetric titration shown in upper panel (lower panel); Figure S56. Raw data of titration of 500 µM [PheOEt][SA] with 27 µM at 25 °C (upper panel). Integrated heat profile of calorimetric titration shown in upper panel (lower panel); Figure S57. Raw data of titration of 500 µM [PheOMe][SA] with 27 µM at 25 °C (upper panel). Integrated heat profile of calorimetric titration shown in upper panel (lower panel).
