# Supplementary material for: Salicylic Acid as Ionic Liquid Formulation May Have Enhanced Potency to Treat Some Chronic Skin Diseases
Source: Molecules. 2021 Dec 30;27(1):216. doi: 10.3390/molecules27010216 (PMC8746858; doi:10.3390/molecules27010216)
Supplement: Supplementary file 1 [file molecules-27-00216-s001.zip › molecules-1491405-supplementary.pdf]

molecules

MDPI

Supplementary Material

Salicylic Acid as Ionic Liquid Formulation May Have Enhanced Potency to Treat Some Chronic Skin Diseases

Joanna Klebeko [1], Paula Ossowicz-Rupniewska [1], Ewelina Świątek [1], Joanna Szachnowska [1], Ewa Janus [1], Stefka G. Taneva [2], Elena Krachmarova [3] and Maya Guncheva [4,*]

[1] Department of Chemical Organic Technology and Polymeric Materials, Faculty of Chemical Technology and Engineering,West Pomeranian University of Technology in Szczecin, Piastów Ave. 42, 71-065 Szczecin, Poland; joanna.klebeko@gmail.com (J.K.); possowicz@zut.edu.pl (P.O.-R.); ewelinaswiatek94@gmail.com (E.Ś.); j.szachnowskaa@gmail.com (J.S.); ejanus@zut.edu.pl (E.J.)

[2] Institute of Biophysics and Biomedical Engineering, Bulgarian Academy of Sciences, Acad. G. Bonchev Str. 21, 1113 Sofia, Bulgaria; sgtaneva@gmail.com

[3] Institute of Molecular Biology "Roumen Tsanev", Bulgarian Academy of Sciences, Acad. G. Bonchev Str, Block 21, 1113 Sofia, Bulgaria; elenakrachmarova@bio21.bas.bg

[4] Institute of Organic Chemistry with Centre of Phytochemistry, Bulgarian Academy of Sciences, Acad. G. Bonchev Bl. 9, 1113 Sofia, Bulgaria

* Correspondence: maya.guncheva@orgchm.bas.bg

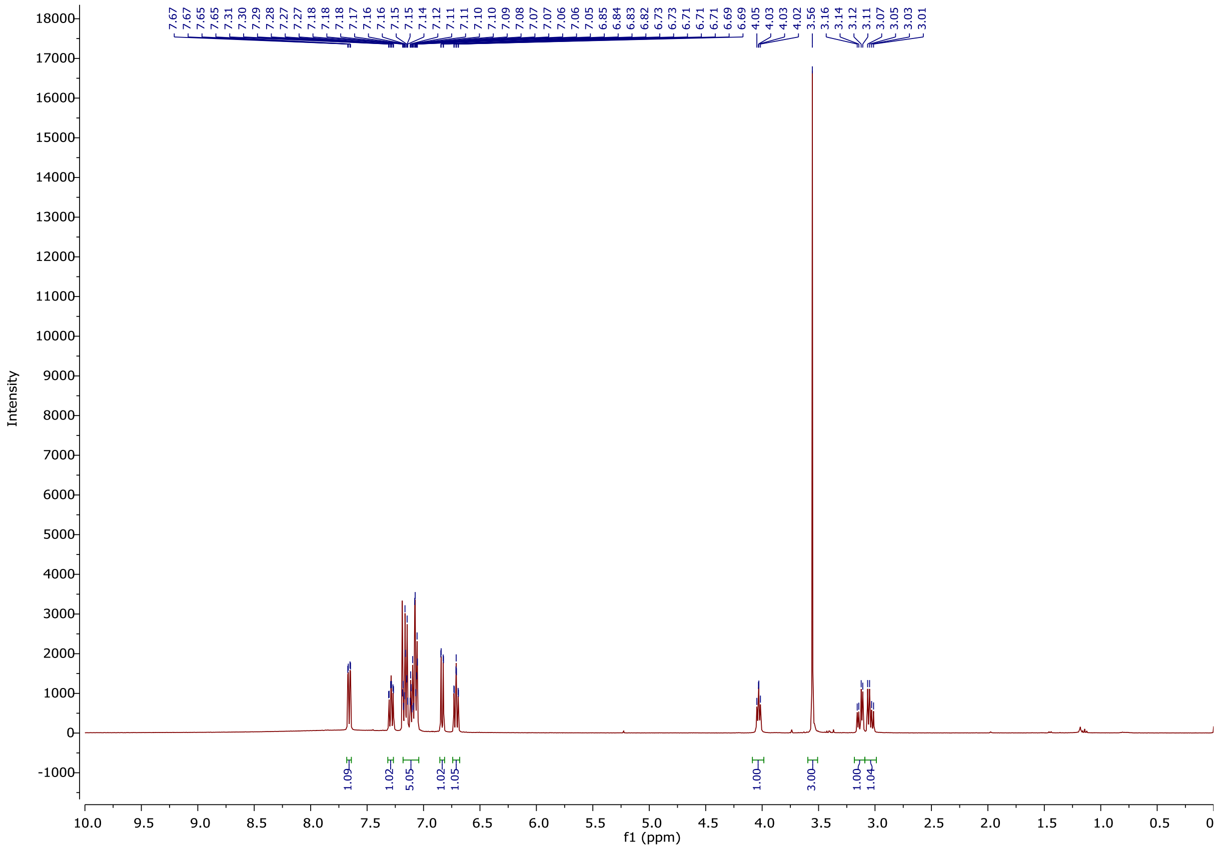

Figure S1. ^{1}H NMR spectra of [PheOMe][SA].

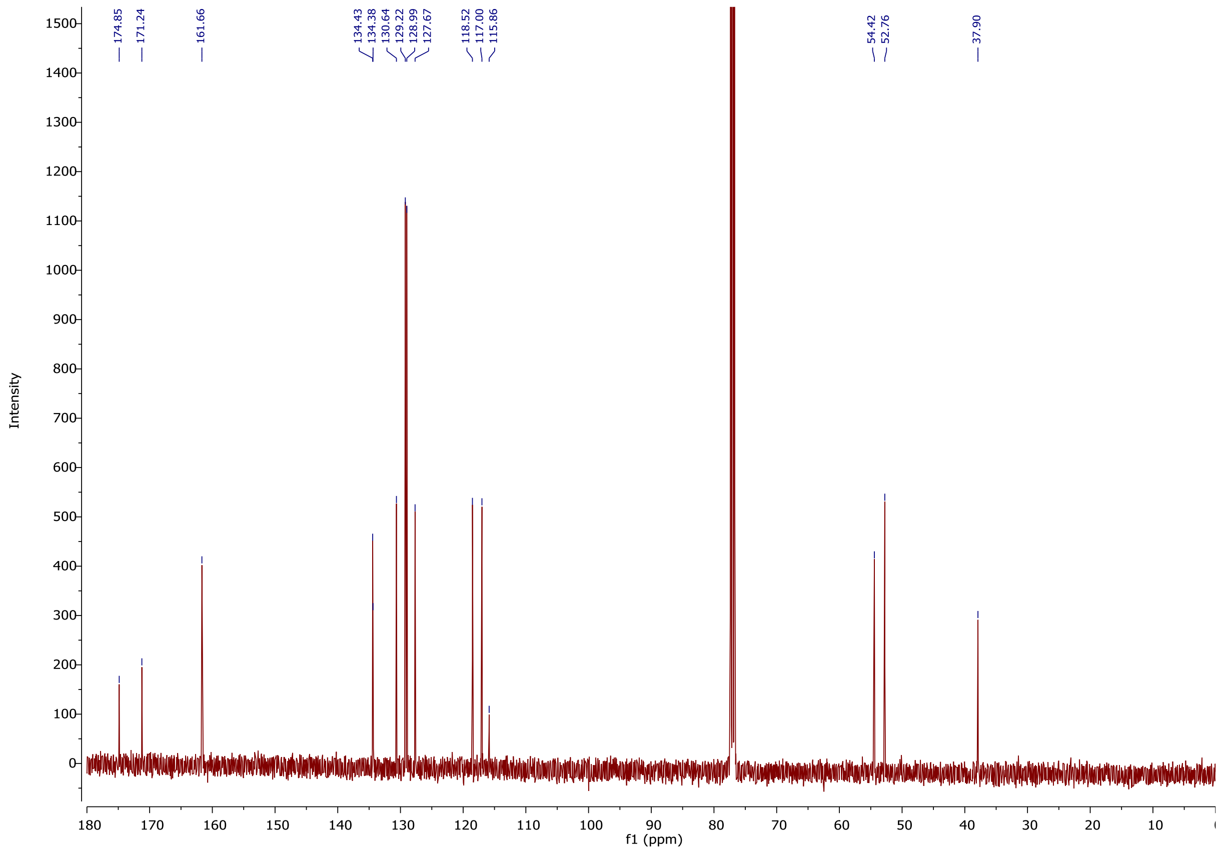

Figure S2. ^{13}C NMR spectra of [PheOMe][SA].

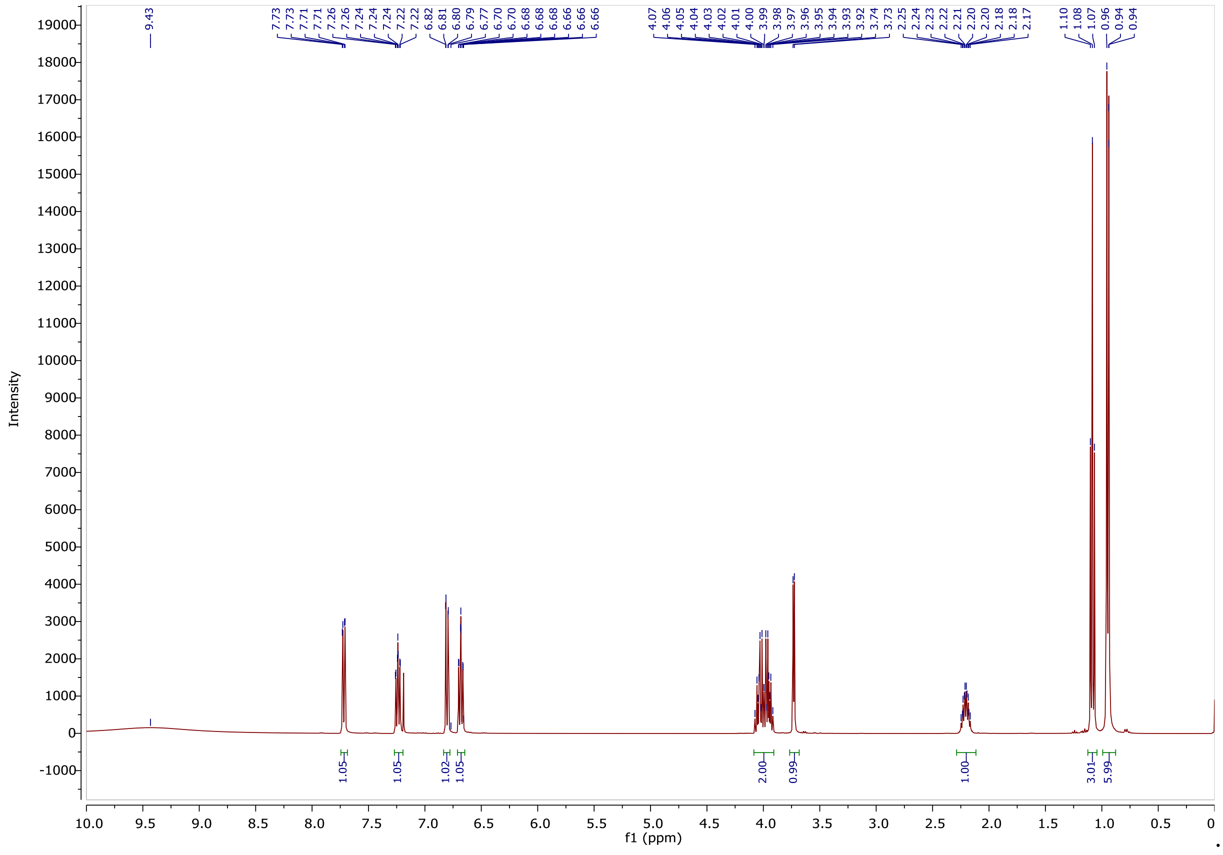

Figure S3. ^{1}H NMR spectra of [ValOEt][SA].

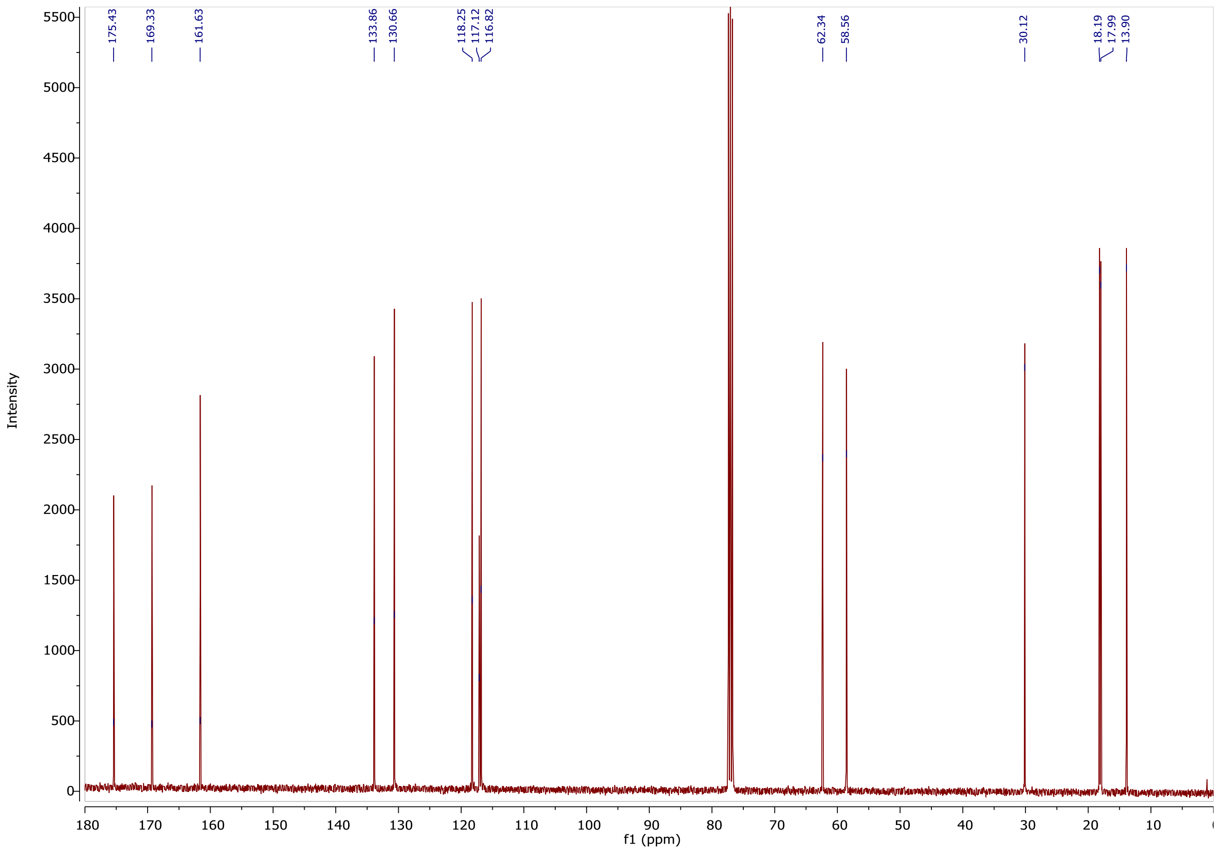

Figure S4. ^{13}C NMR spectra of [ValOEt][SA].

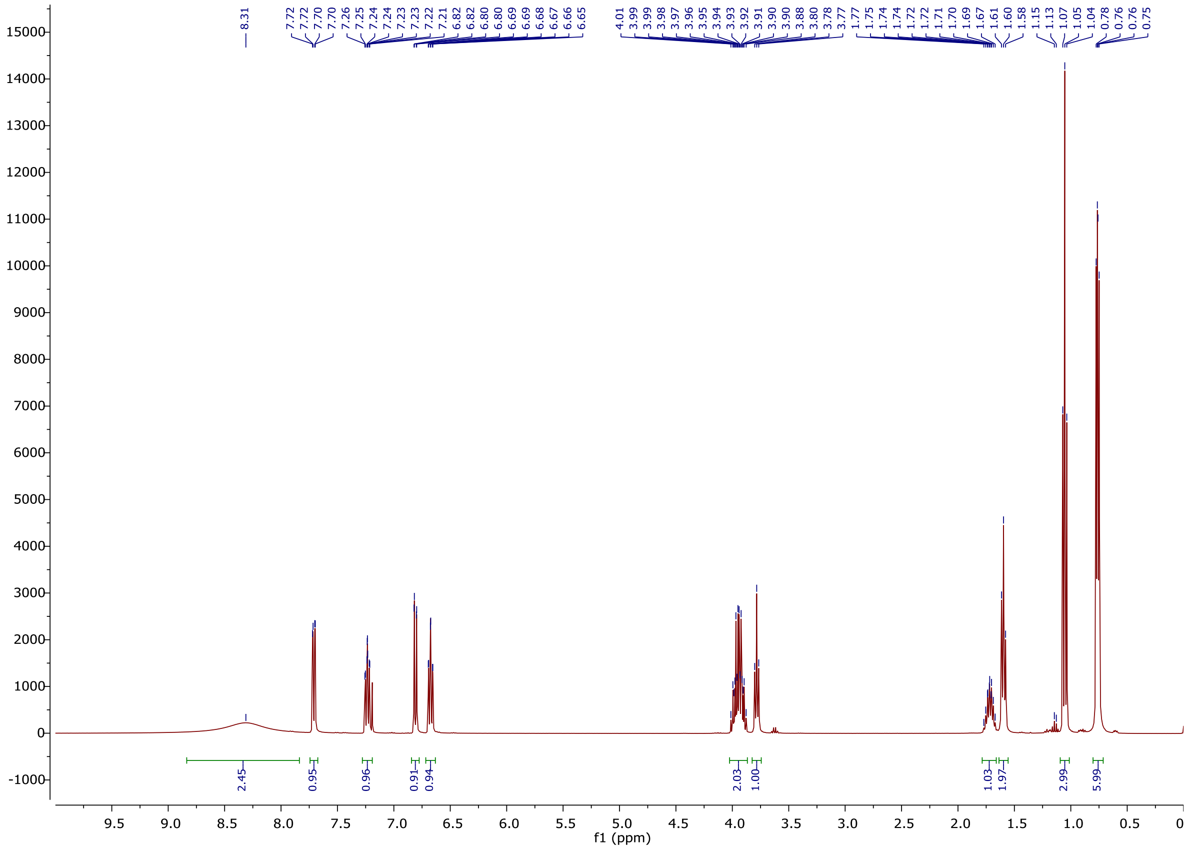

Figure S5. ^{1}H NMR spectra of [LeuOEt][SA].

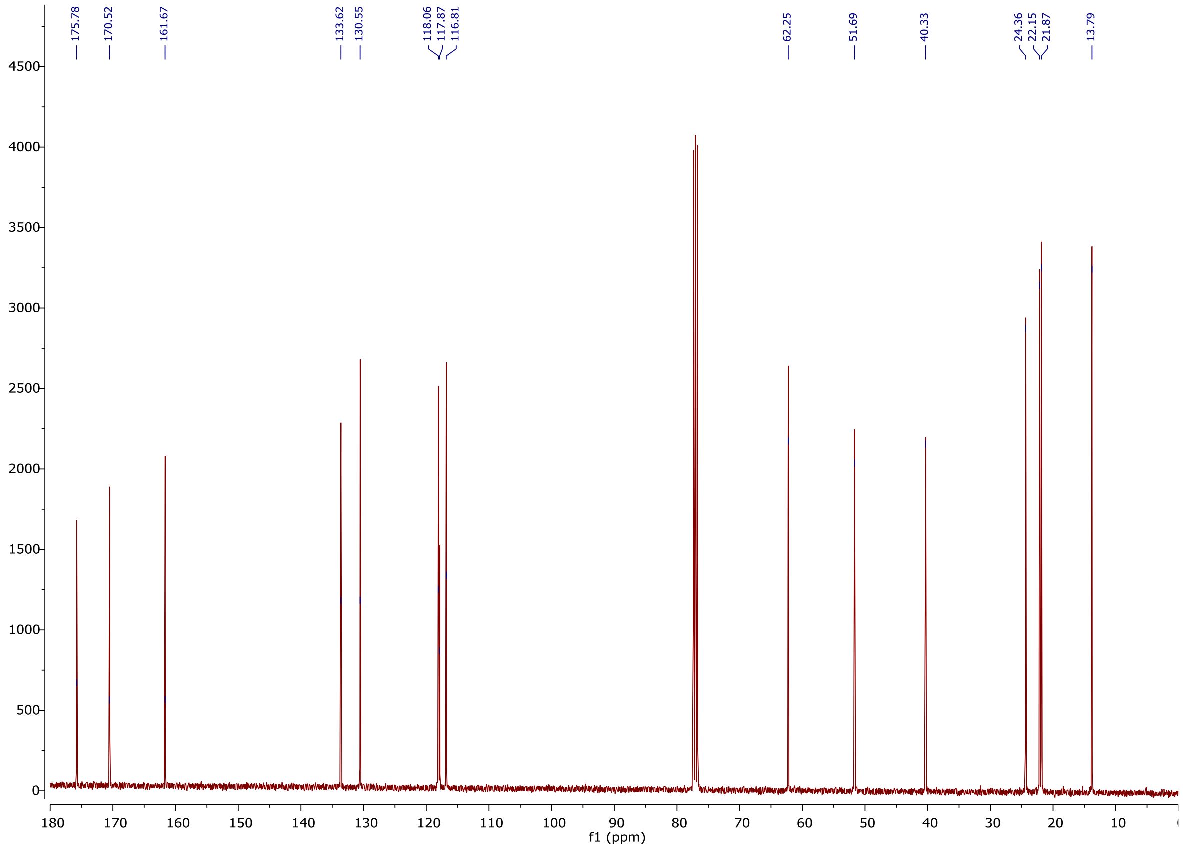

Figure S6. ^{13}C NMR spectra of [LeuOEt][SA].

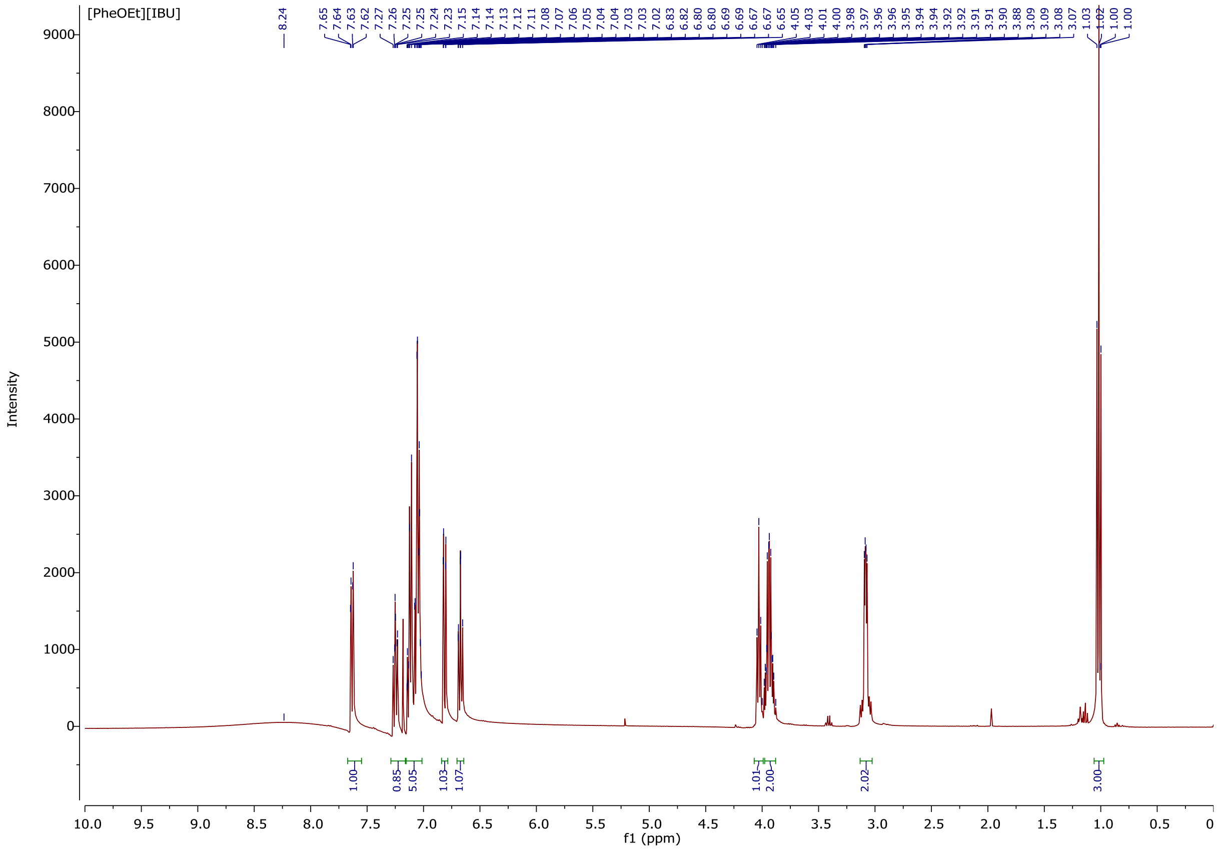

Figure S7. ^{1}H NMR spectra of [PheOEt][SA].

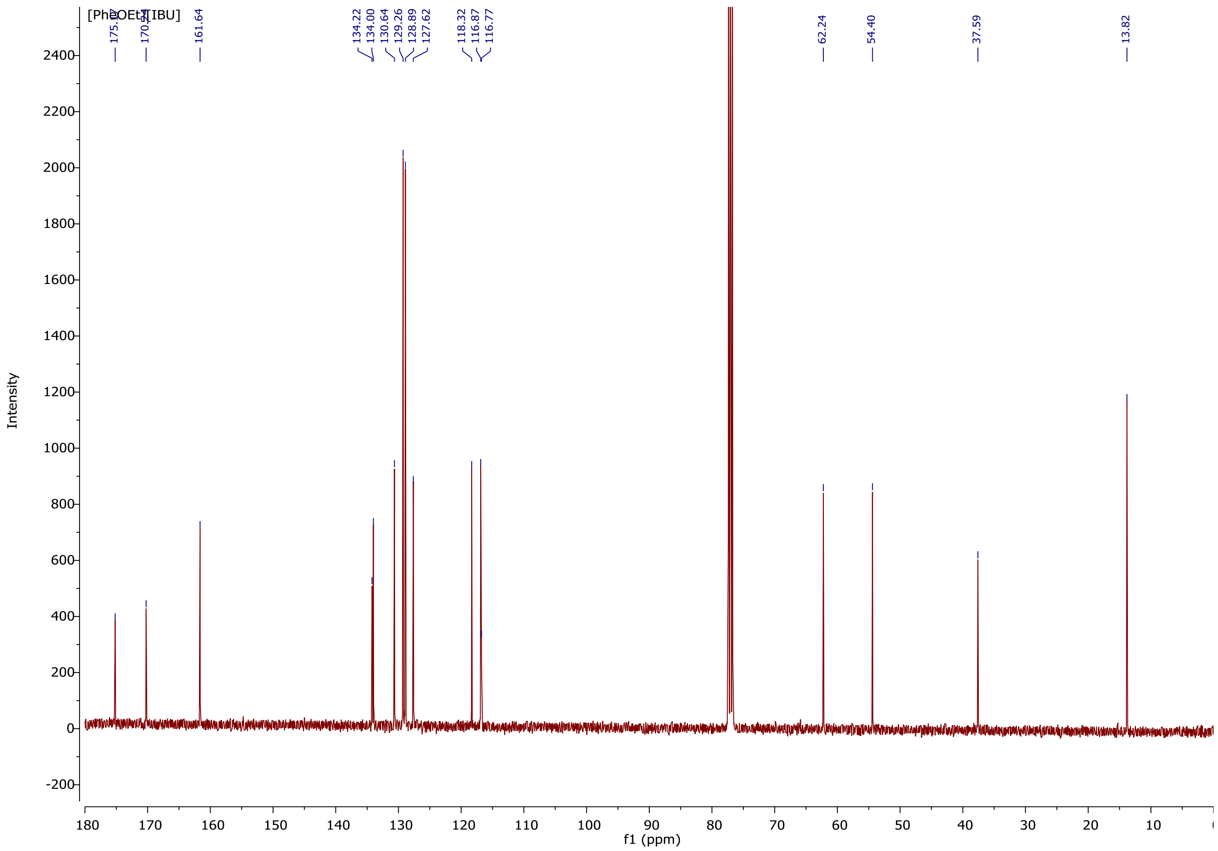

Figure S8. ^{13}C NMR spectra of [PheOEt][SA].

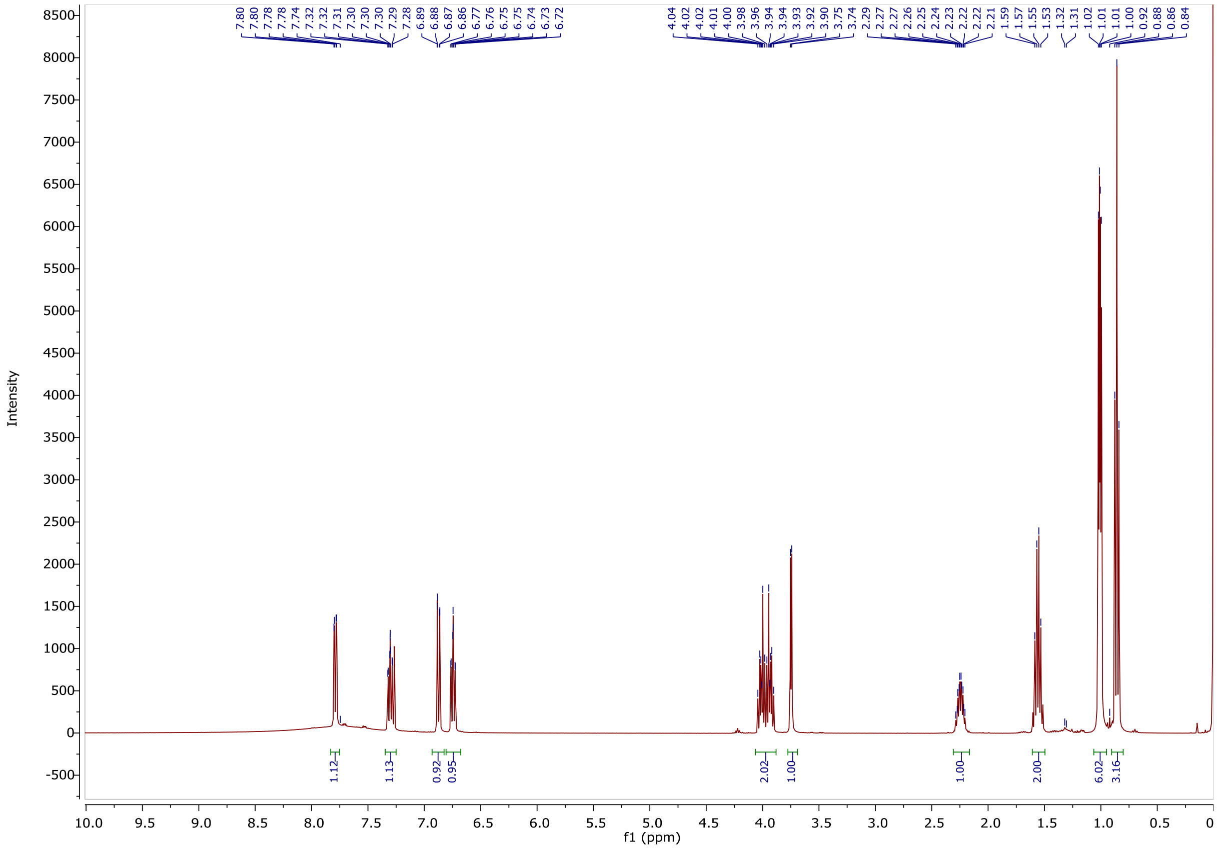

Figure S9. ^{1}H NMR spectra of [ValOPr][SA].

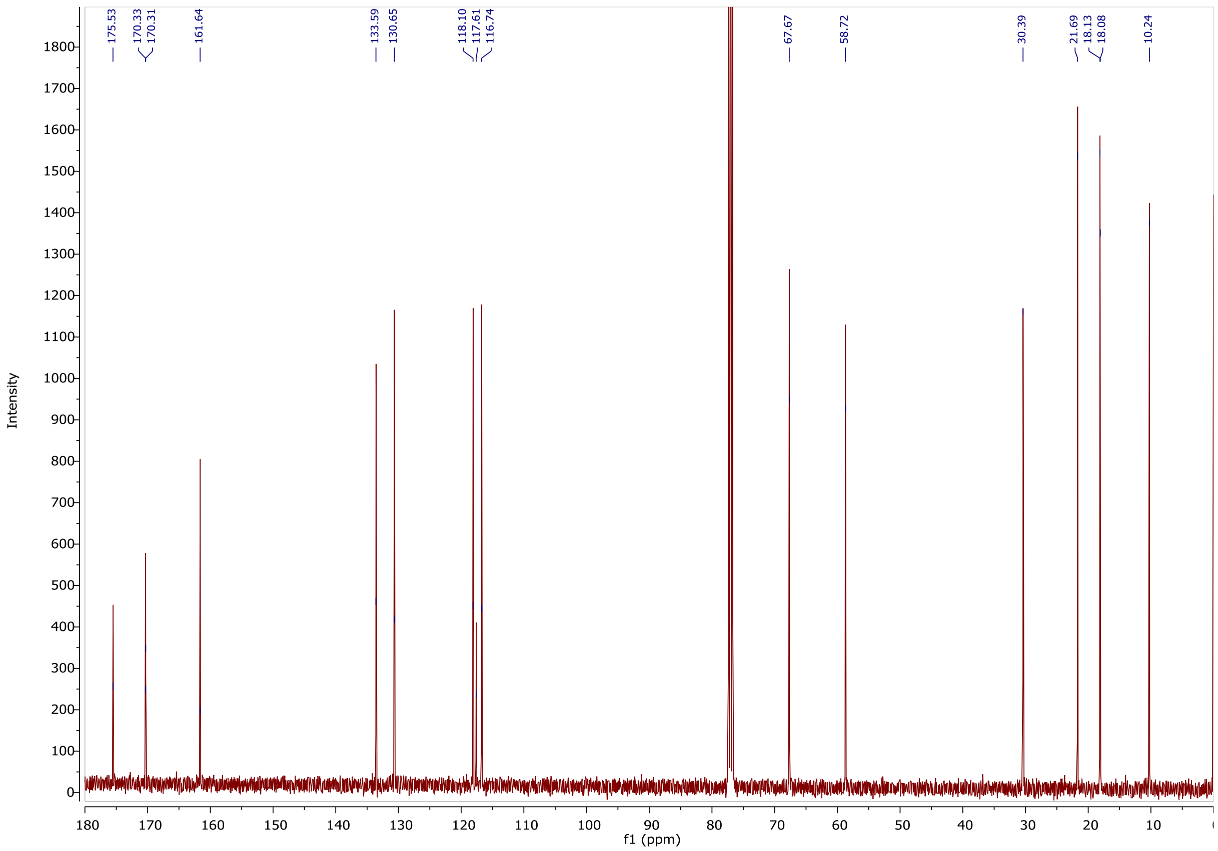

Figure S10. ^{13}C NMR spectra of [ValOPr][SA].

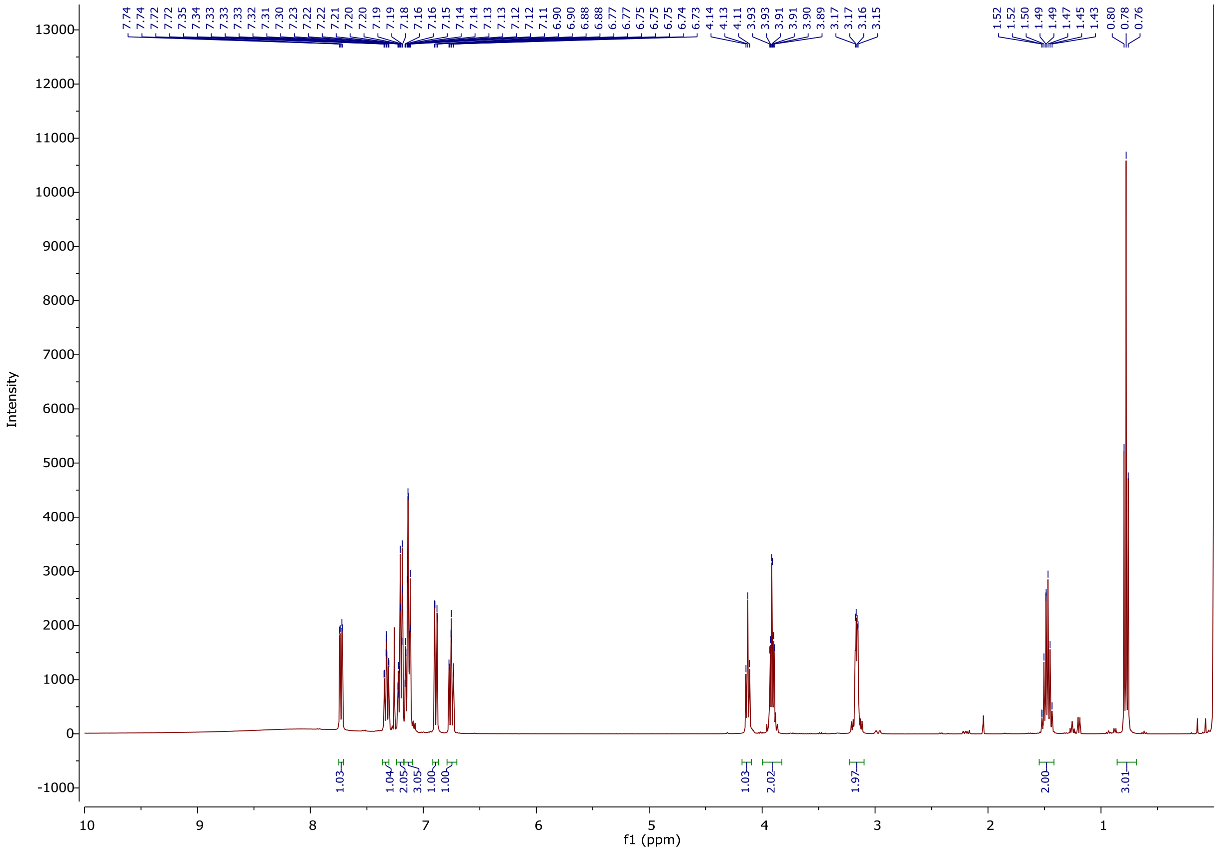

Figure S11. ^{1}H NMR spectra of [PheOPr][SA].

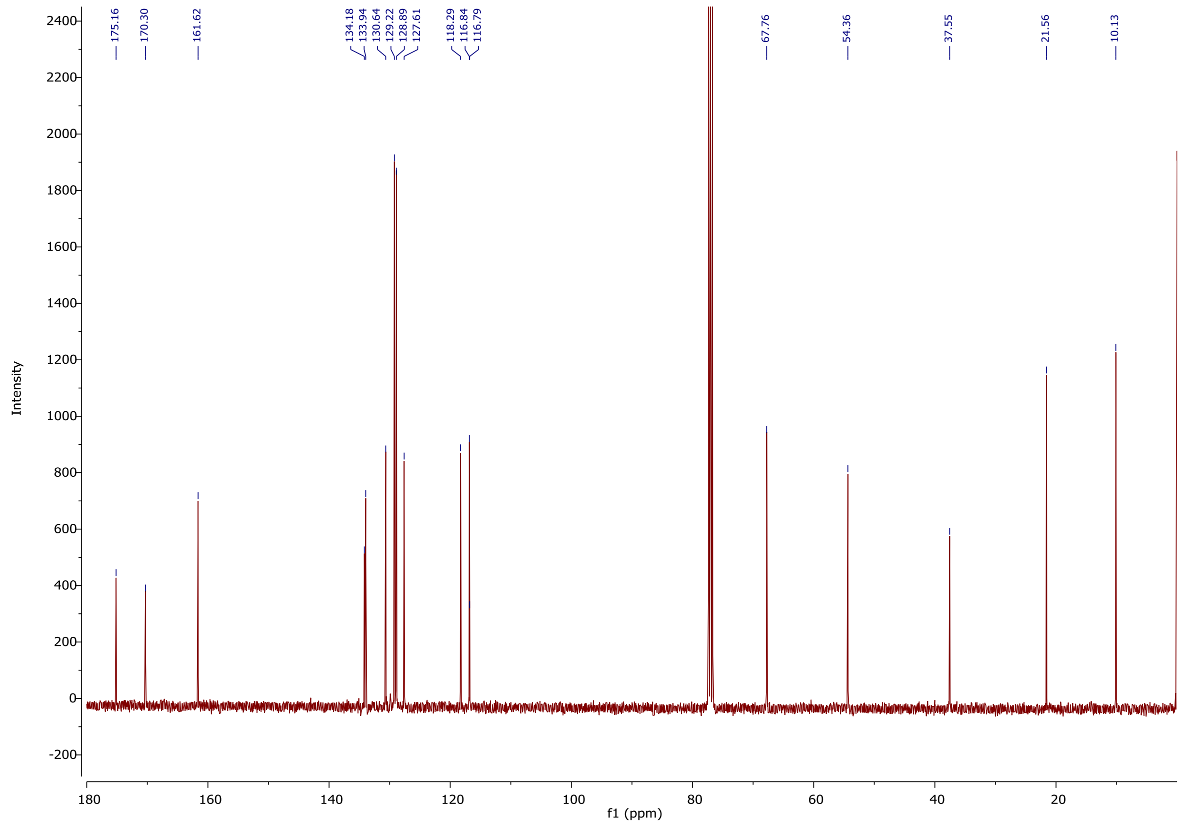

Figure S12. ^{13}C NMR spectra of [PheOPr][SA].

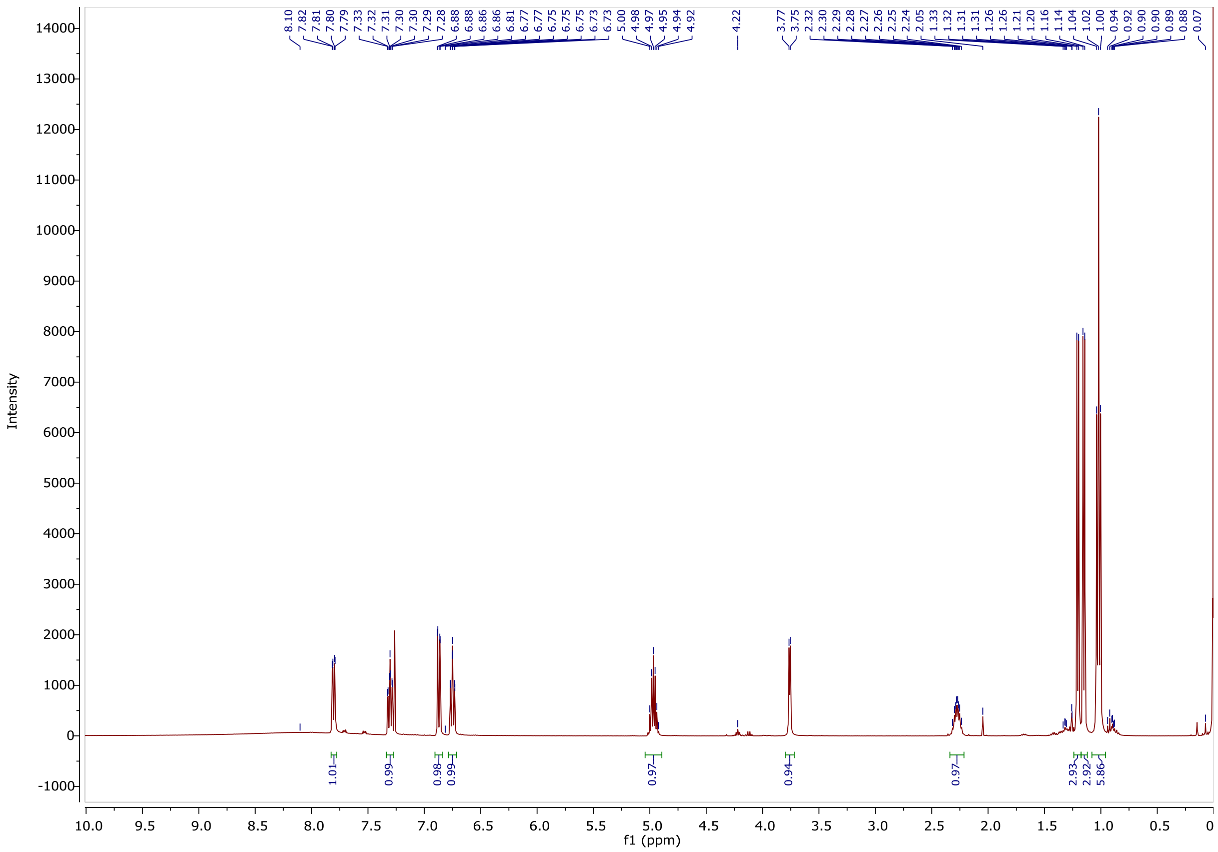

Figure S13. ^{1}H NMR spectra of [ValOiPr][SA].

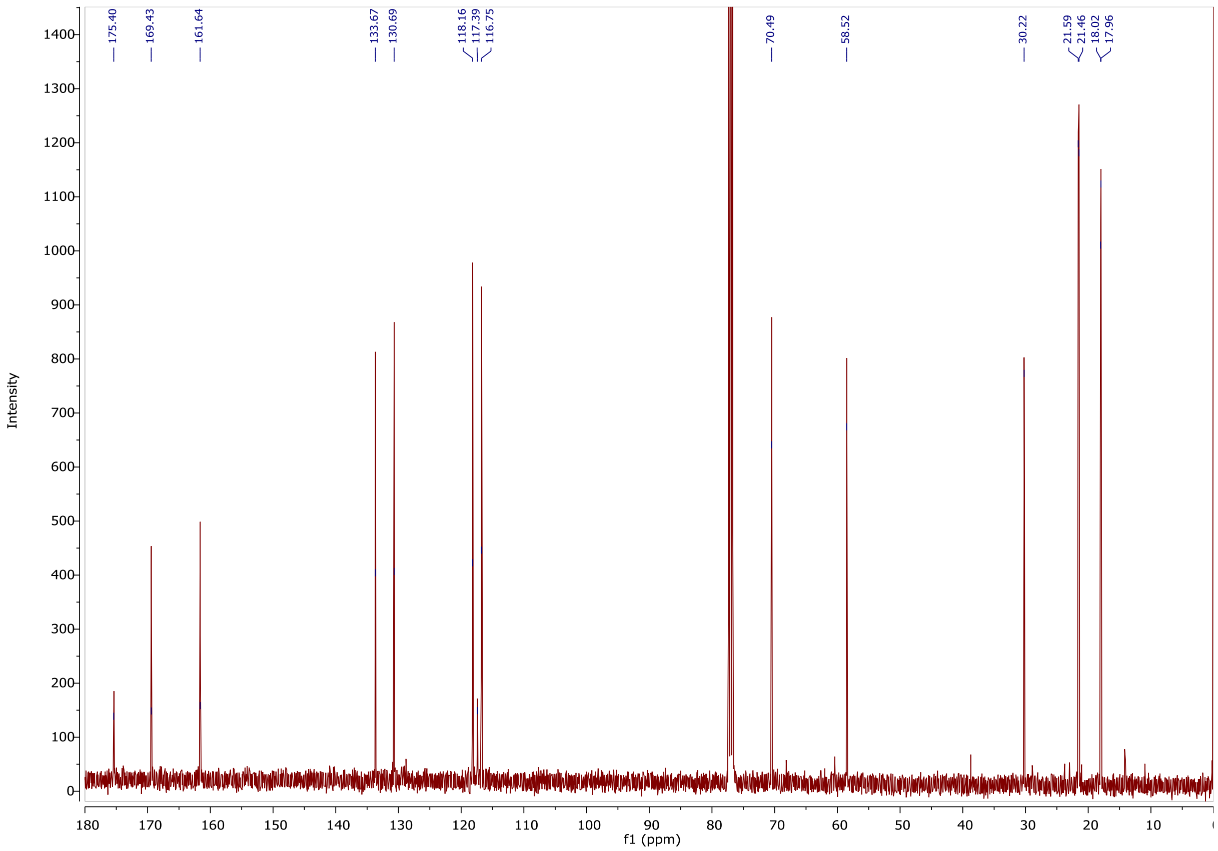

Figure S14. ^{13}C NMR spectra of [ValOiPr][SA].

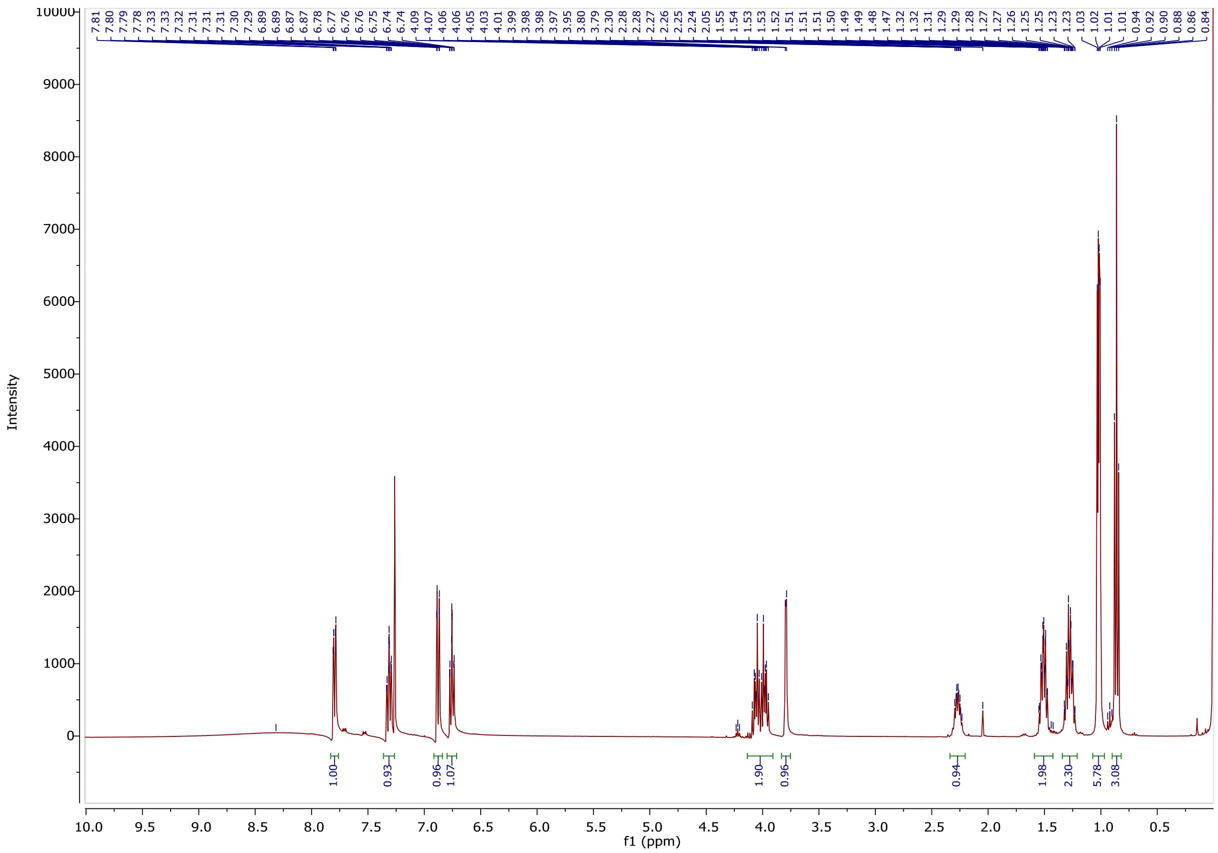

Figure S15. ^{1}H NMR spectra of [ValOBu][SA].

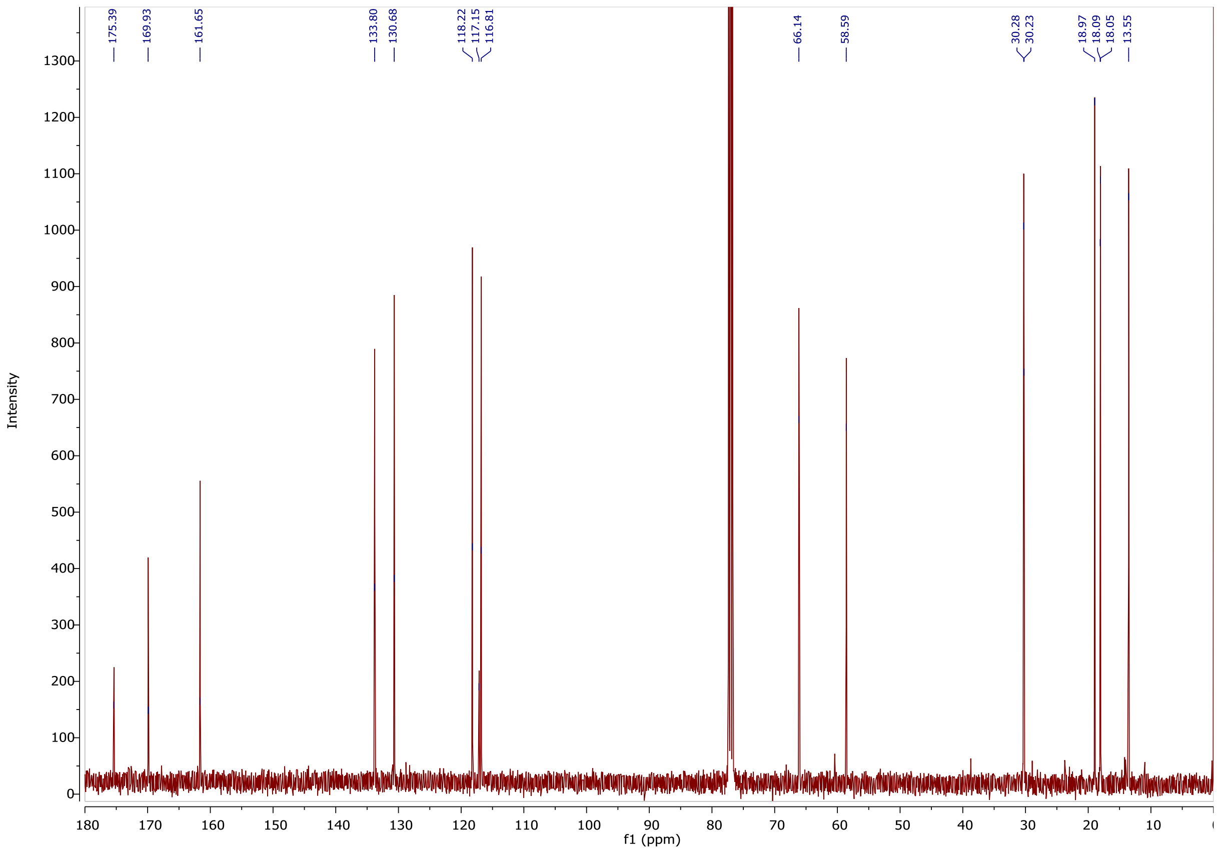

Figure S16. ^{13}C NMR spectra of [ValOBu][SA].

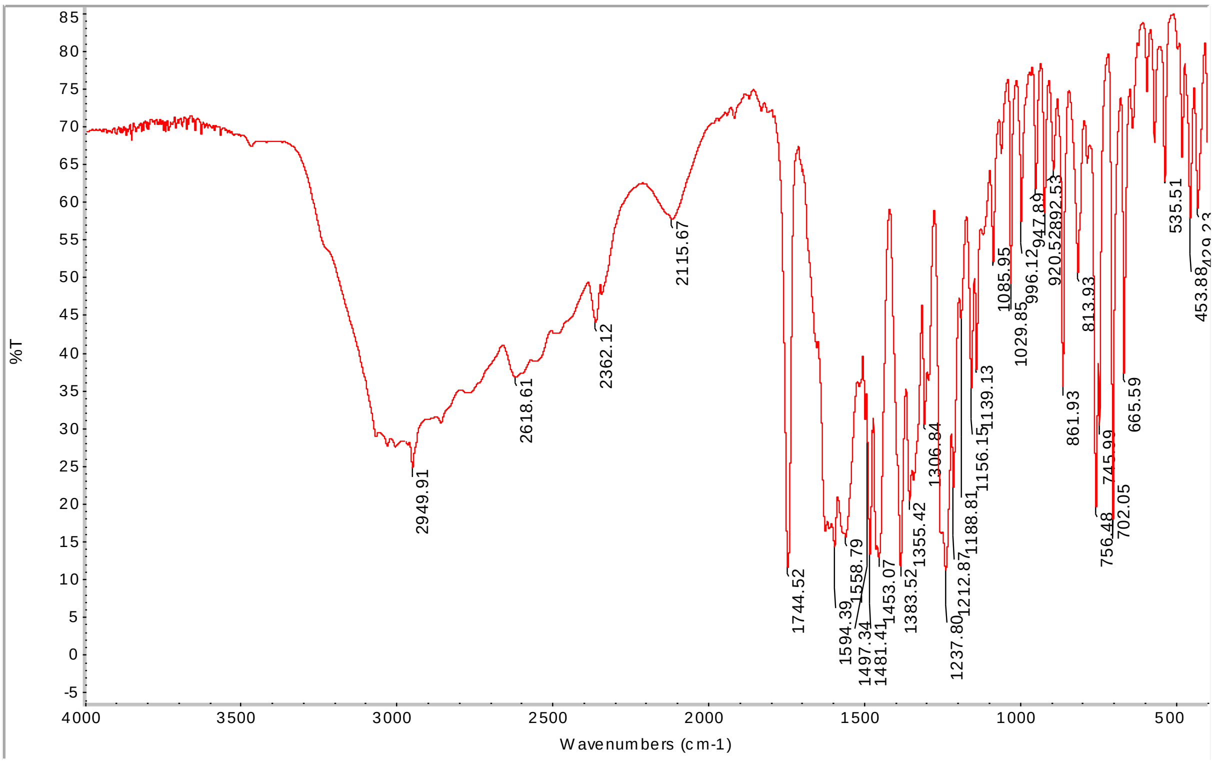

Figure S17. FT-IR spectra of [PheOMe][SA].

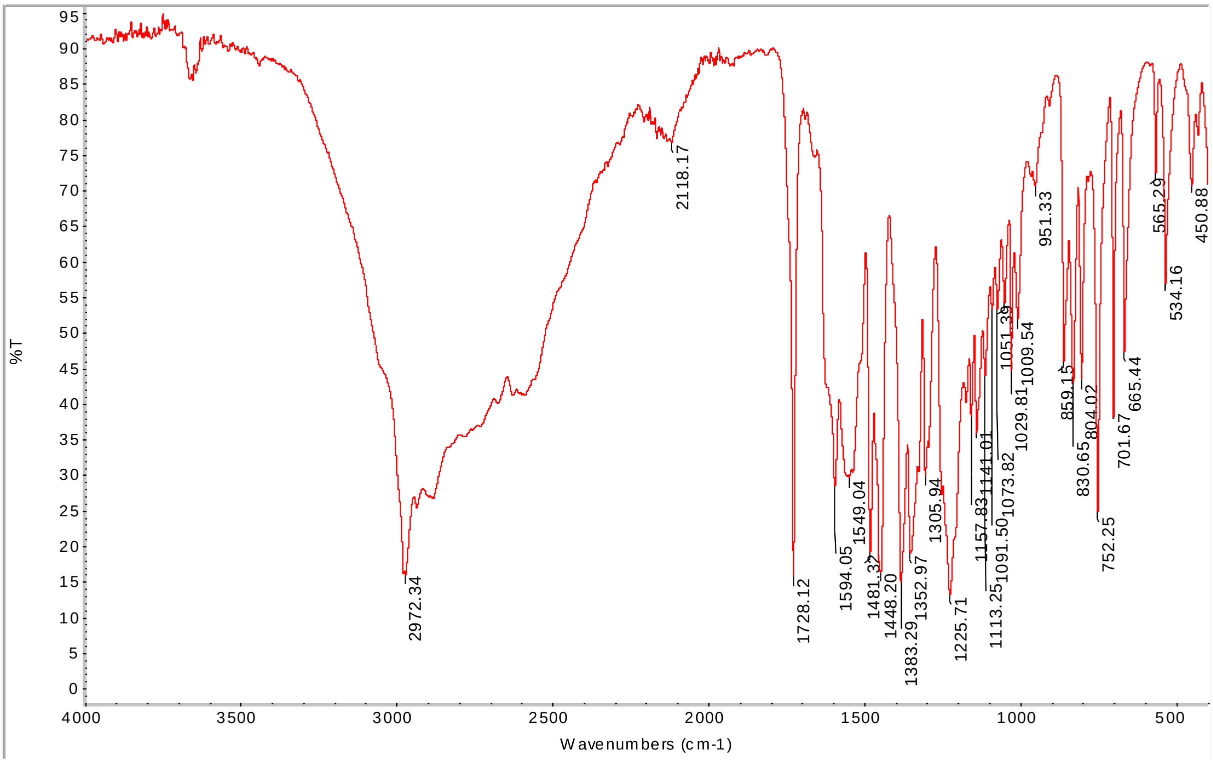

Figure S18. FT-IR spectra of [ValOEt][SA].

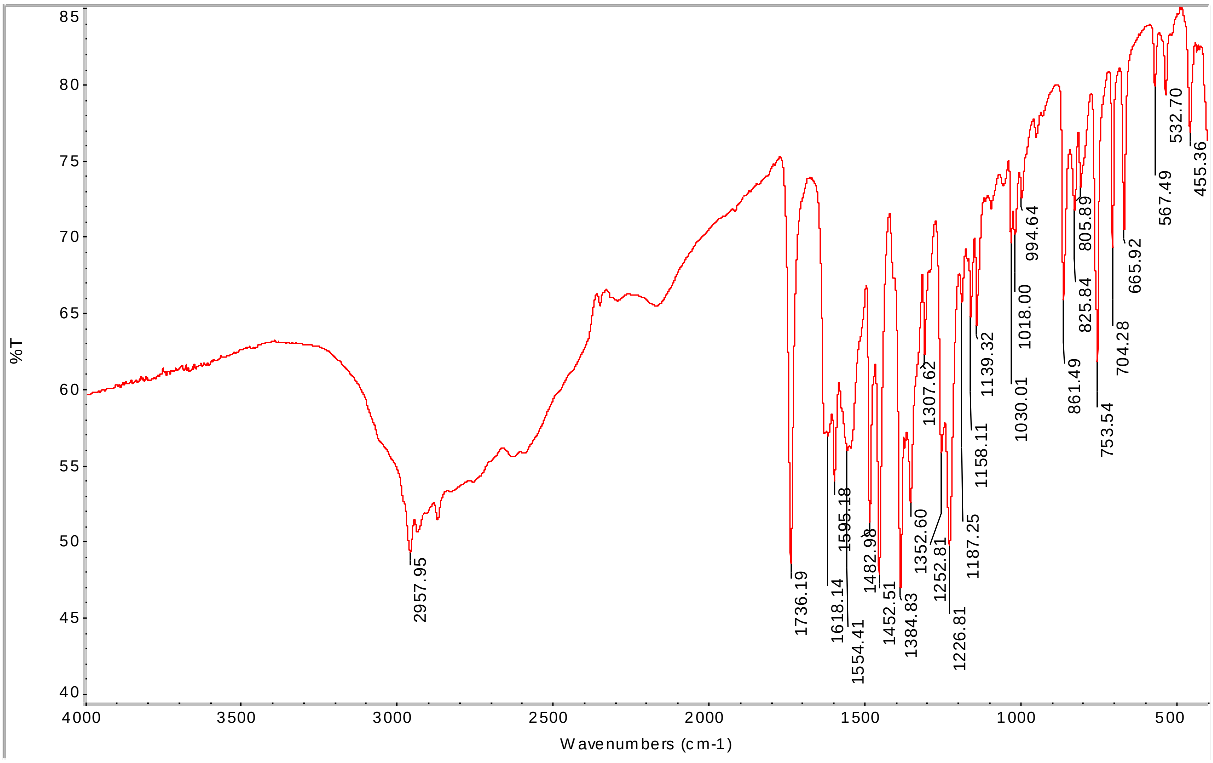

Figure S19. FT-IR spectra of [LeuOEt][SA].

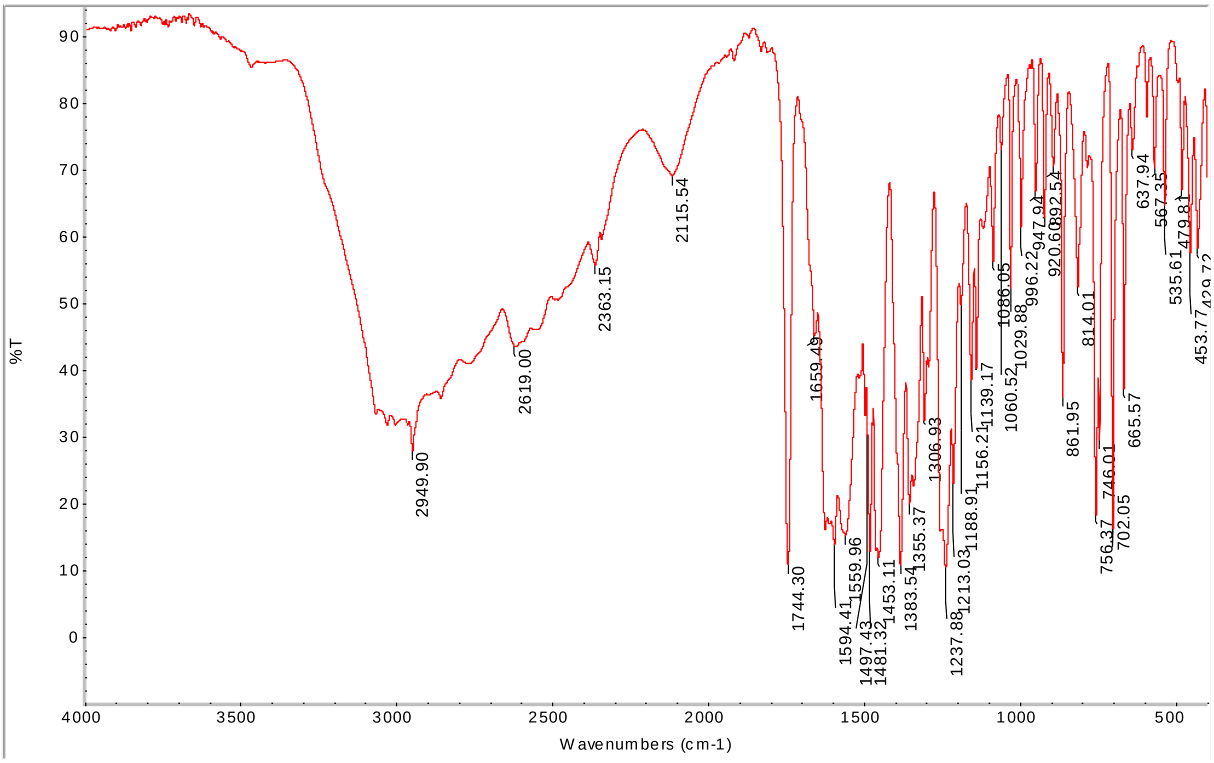

Figure S20. FT-IR spectra of [PheOEt][SA].

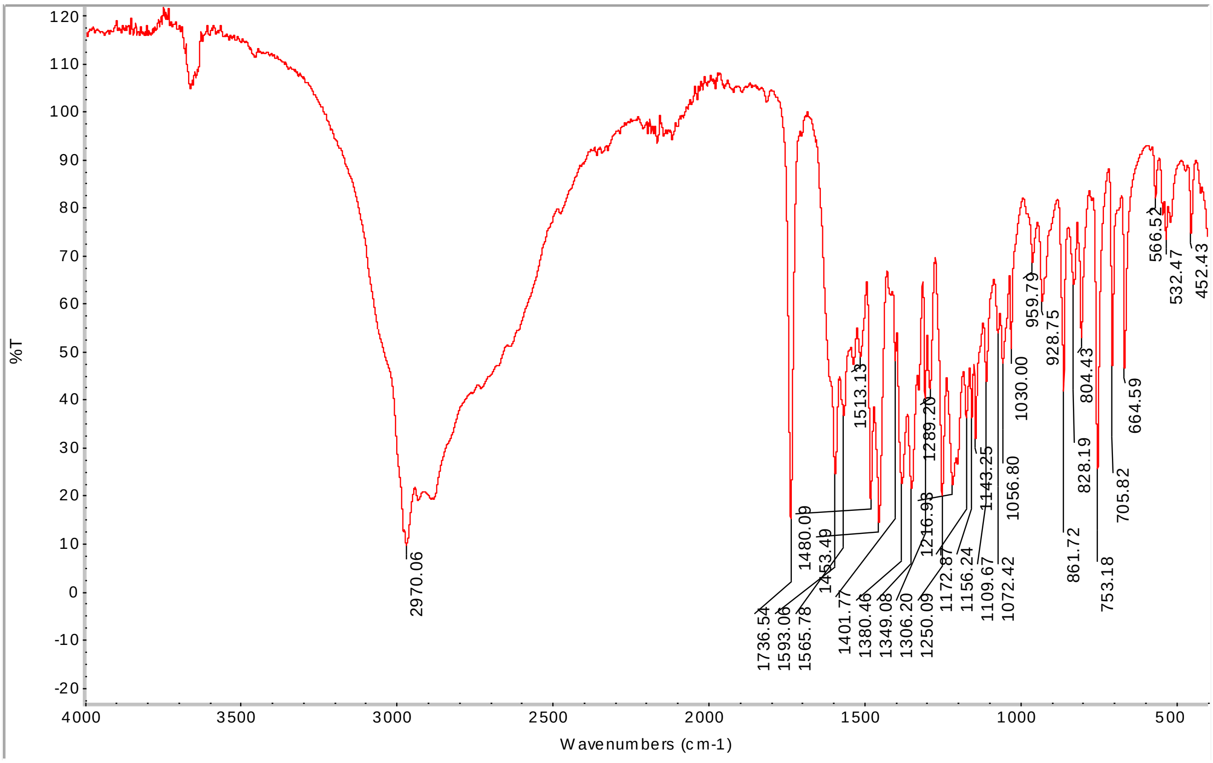

Figure S21. FT-IR spectra of [ValOPr][SA].

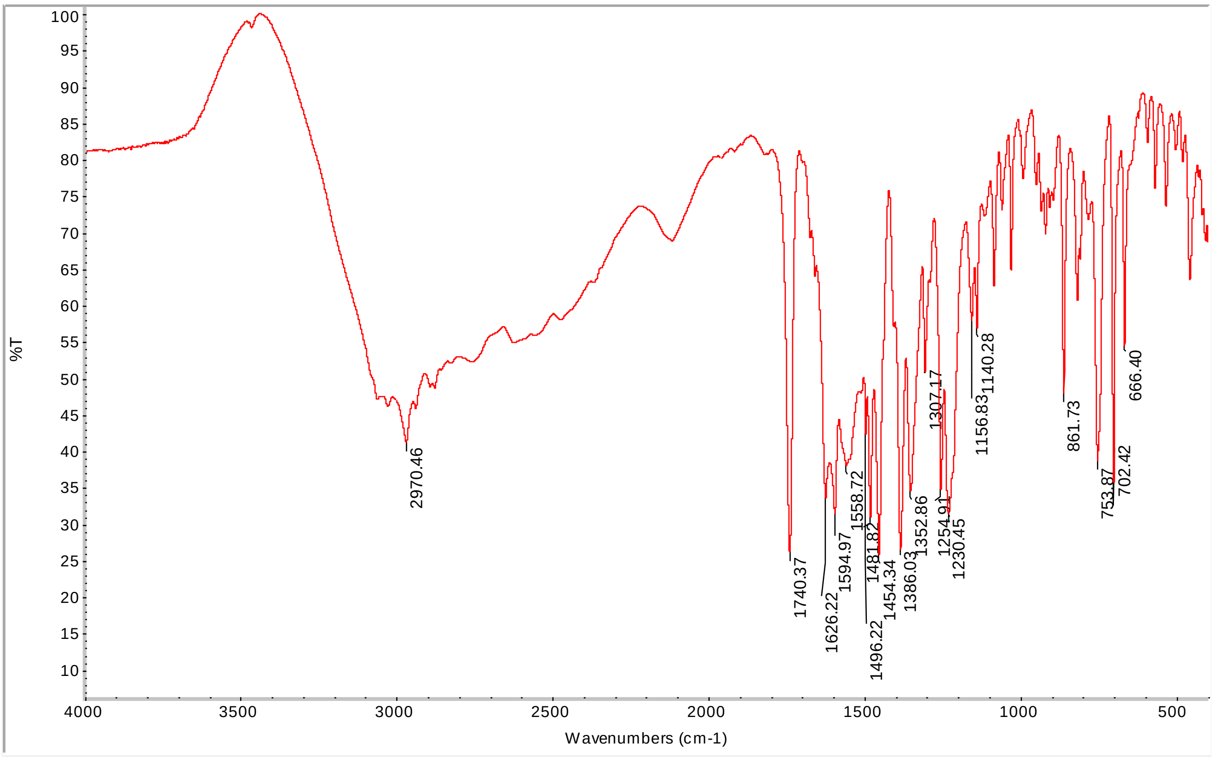

Figure S22. FT-IR spectra of [PheOPr][SA].

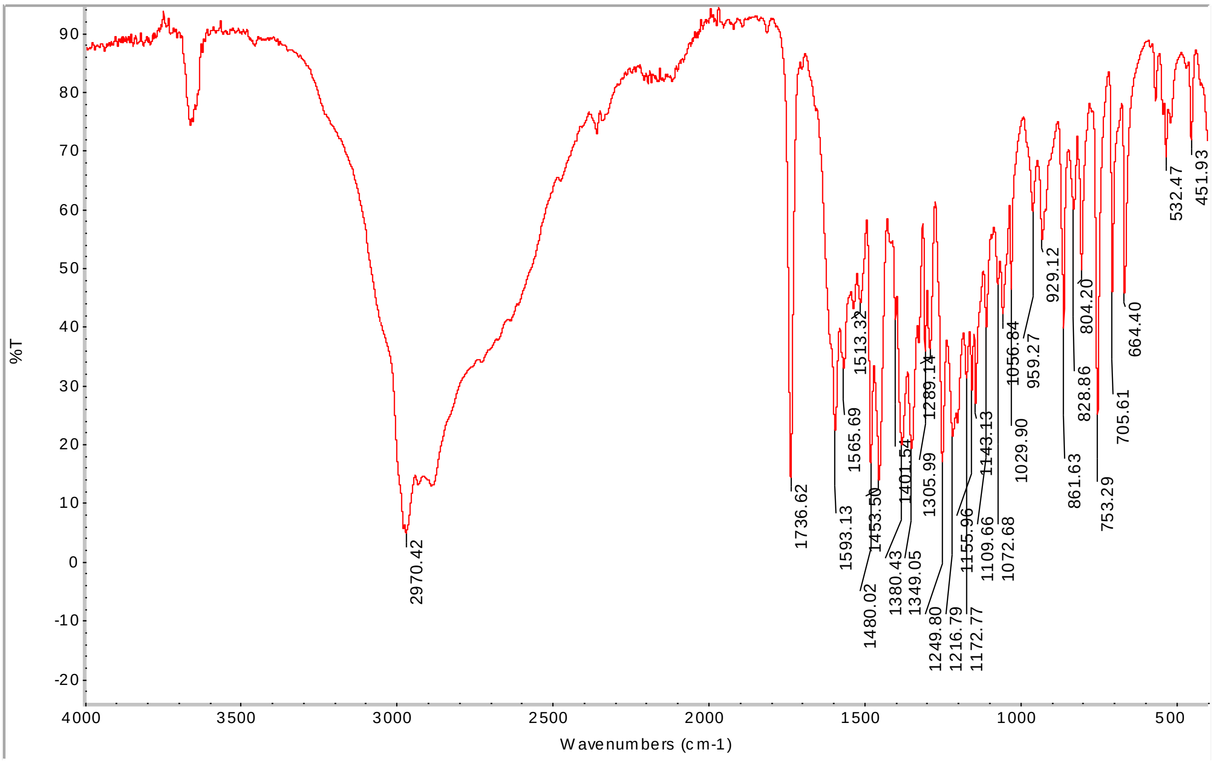

Figure S23. FT-IR spectra of [ValOiPr][SA].

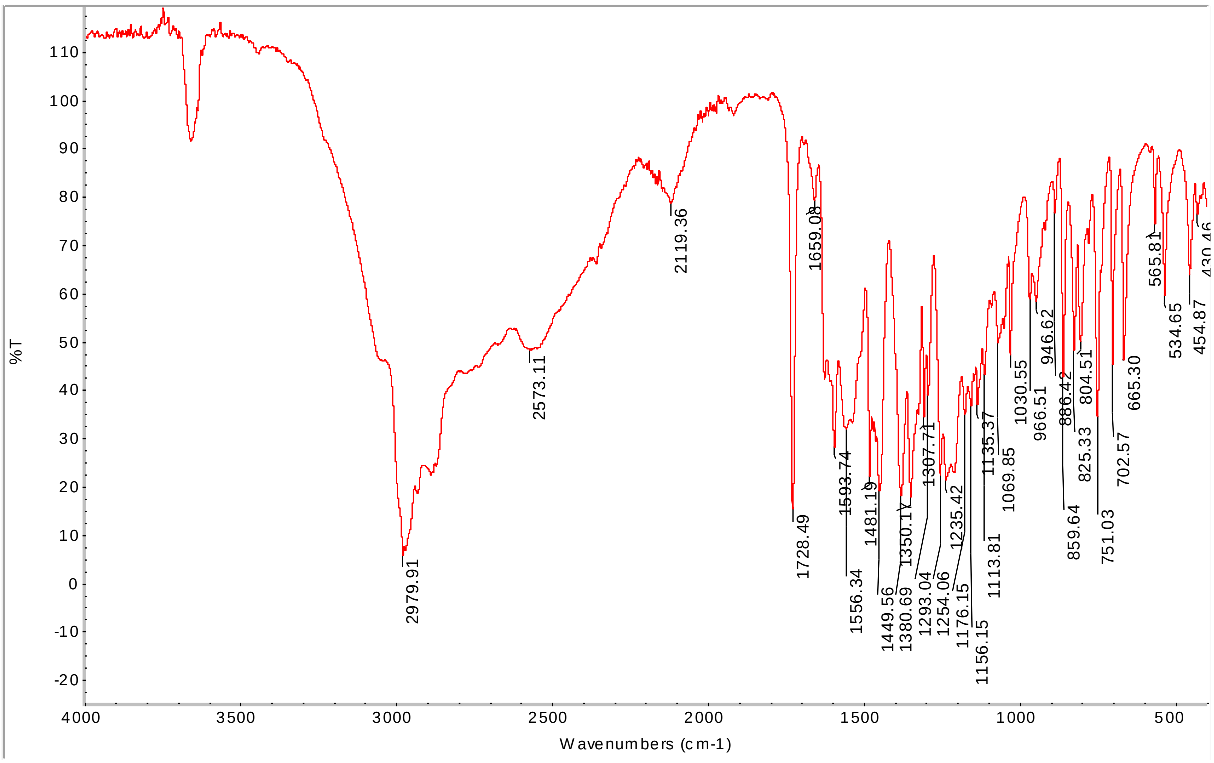

Figure S24. FT-IR spectra of [ValOBu][SA].

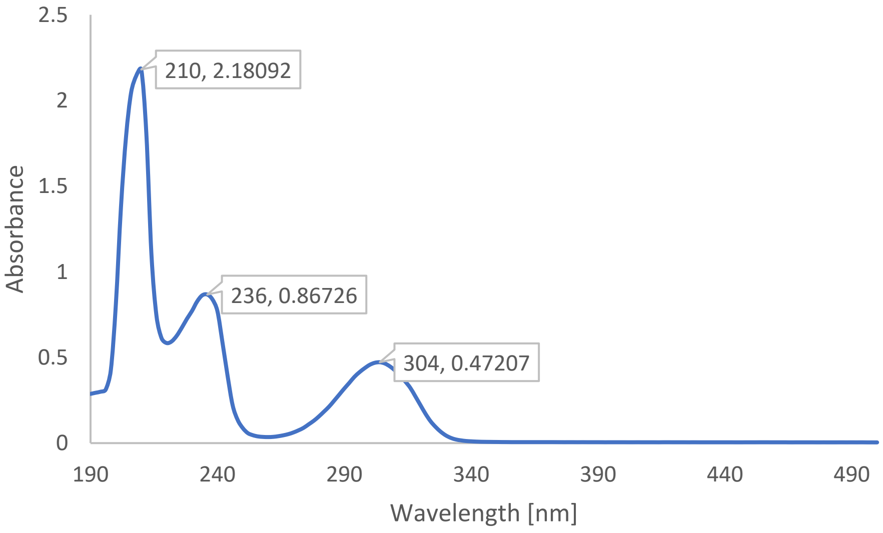

Figure S25. UV-Vis spectra of [PheOMe][SA].

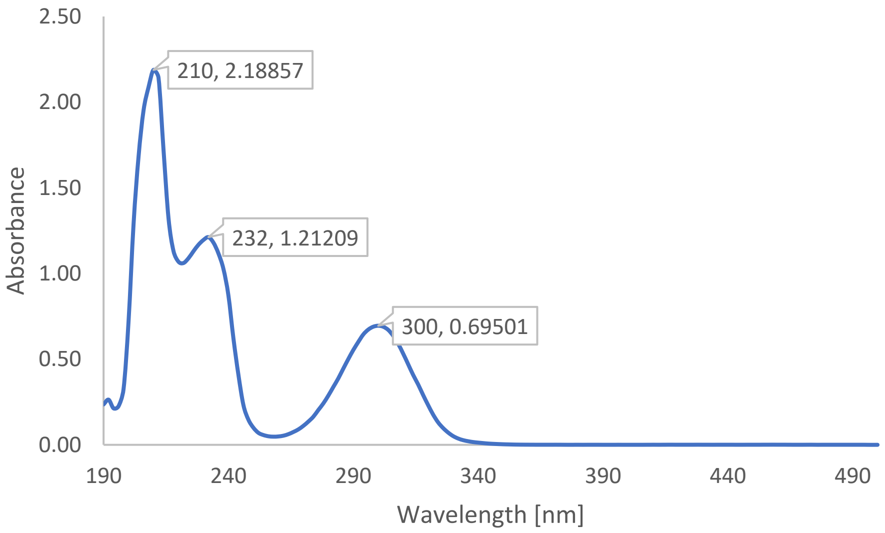

Figure S26. UV-Vis spectra of [ValOEt][SA].

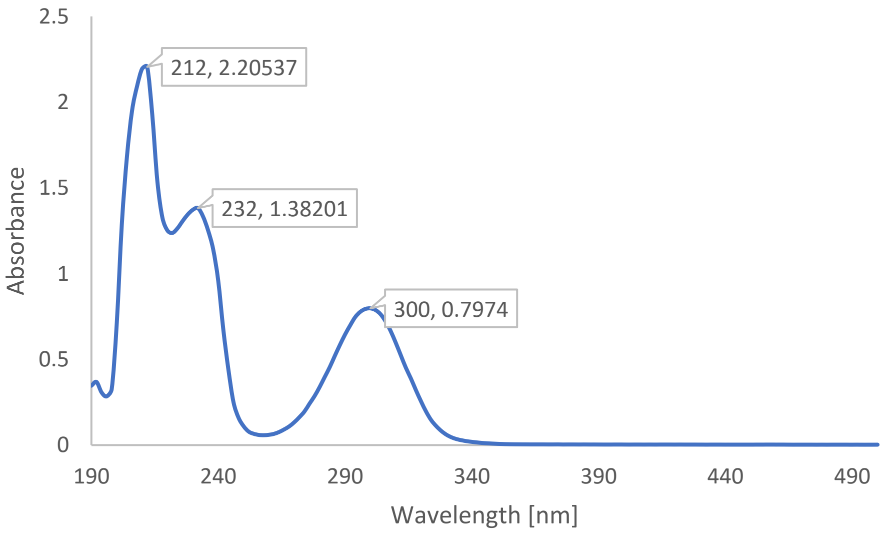

Figure S27. UV-Vis spectra of [LeuOEt][SA].

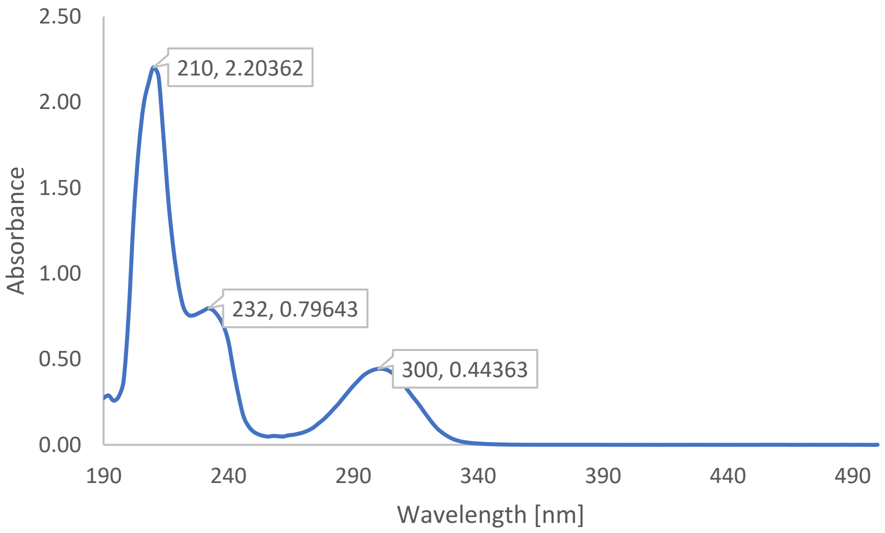

Figure S28. UV-Vis spectra of [PheOEt][SA].

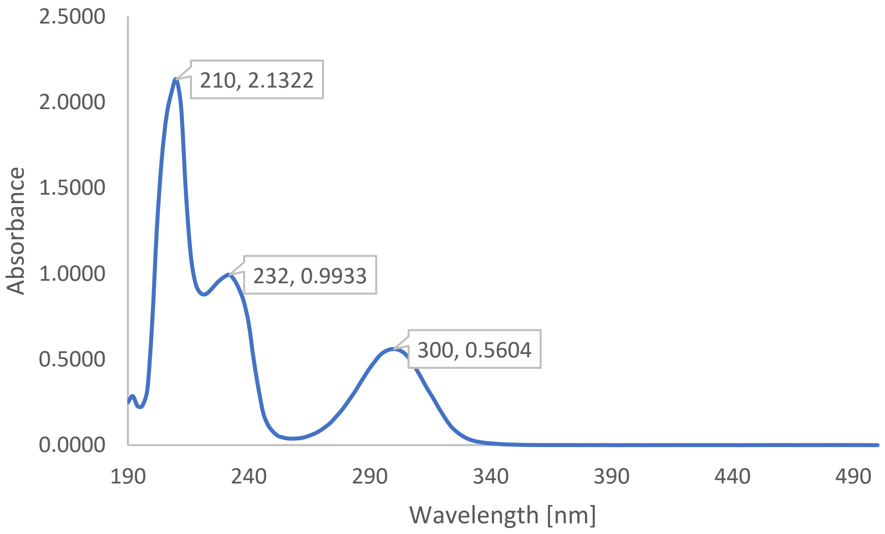

Figure S29. UV-Vis spectra of [ValOPr][SA].

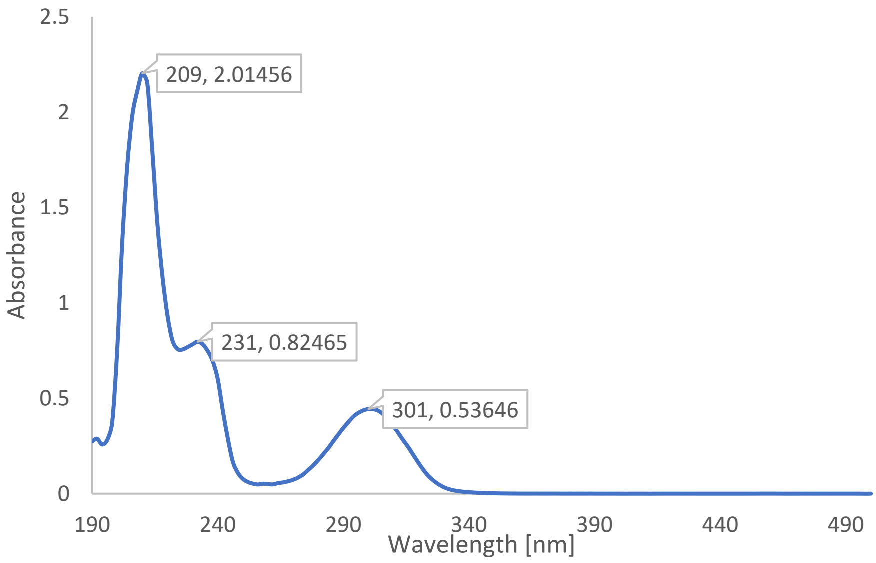

Figure S30. UV-Vis spectra of [PheOPr][SA].

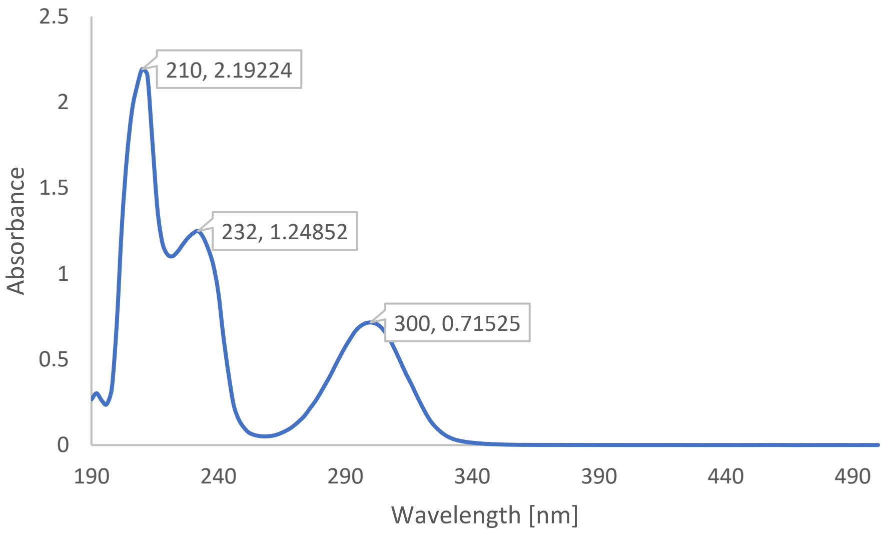

Figure S31. UV-Vis spectra of [ValOiPr][SA].

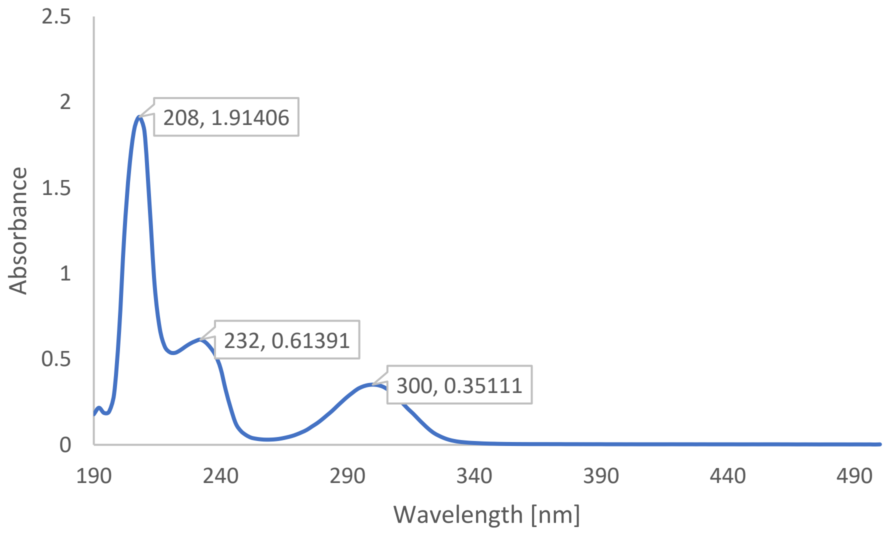

Figure S32. UV-Vis spectra of [ValOBu][SA].

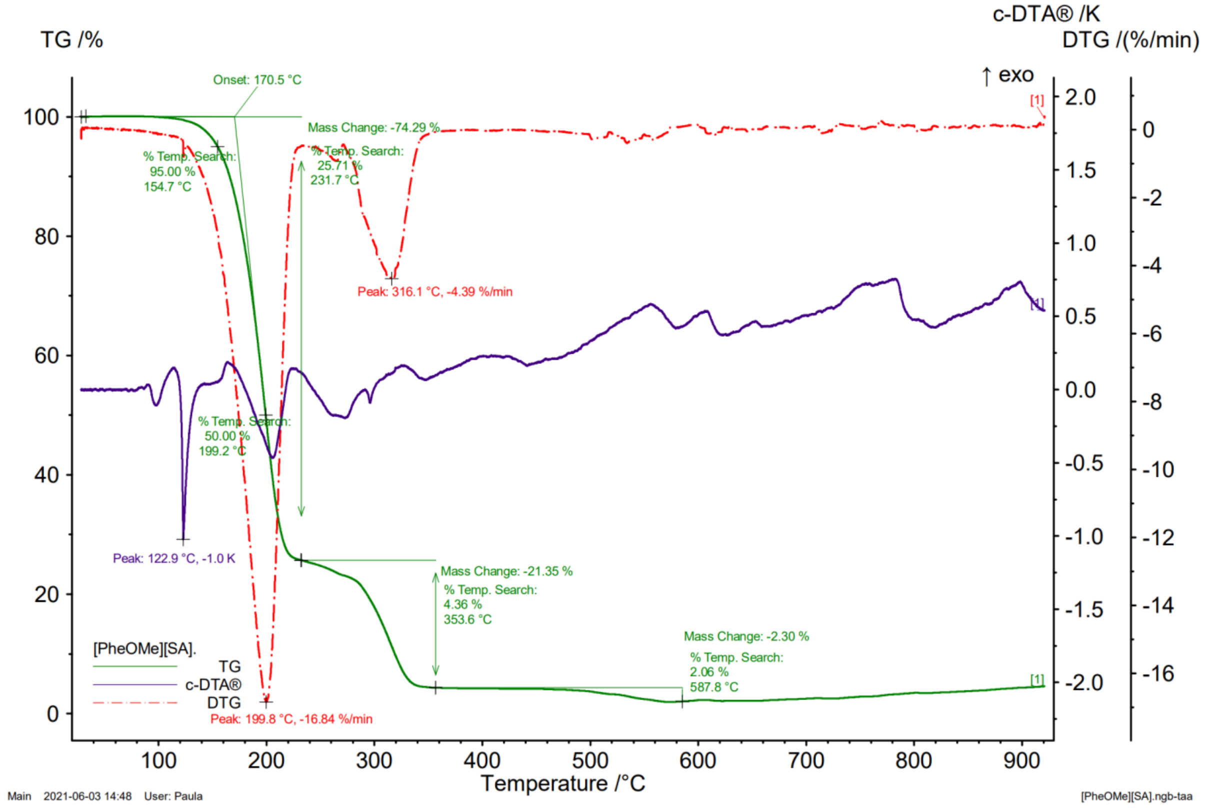

Figure S33. The TG, DTG, and c-DTA curves of [PheOMe][SA].

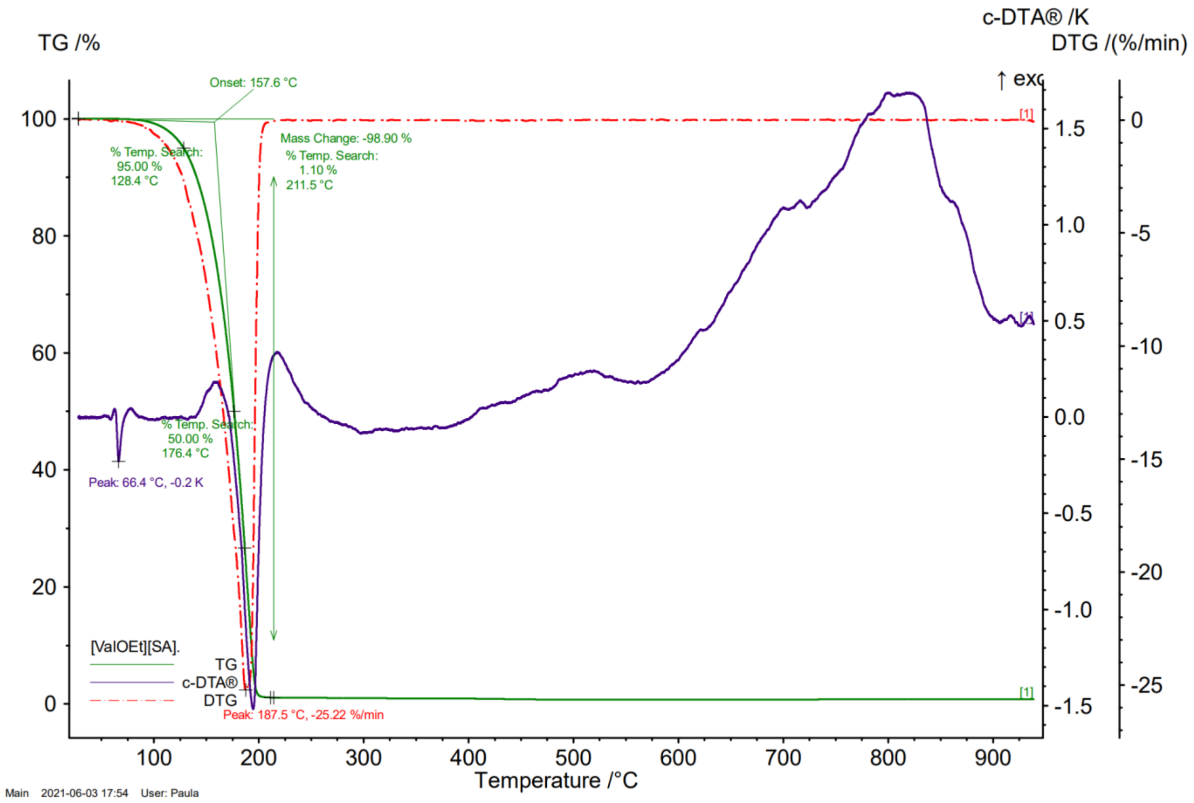

Figure S34. The TG, DTG, and c-DTA curves of [ValOEt][SA].

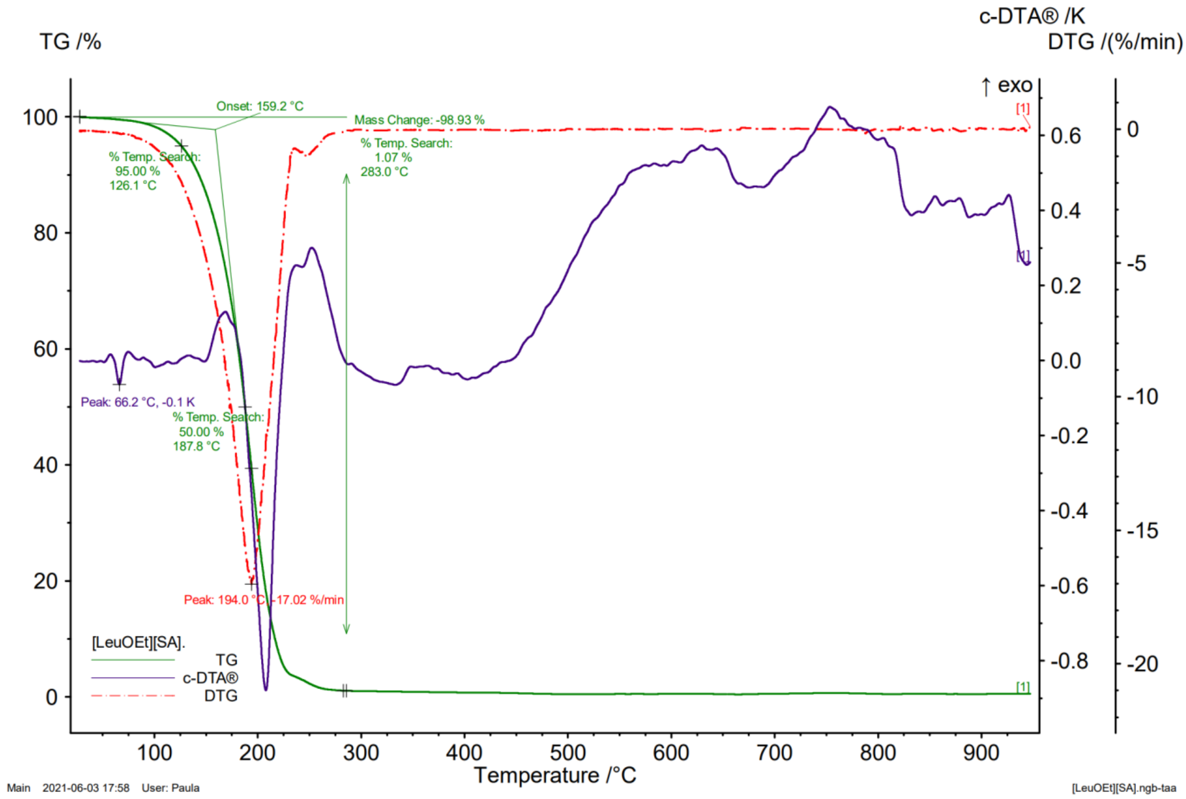

Figure S35. The TG, DTG, and c-DTA curves of [LeuOEt][SA].

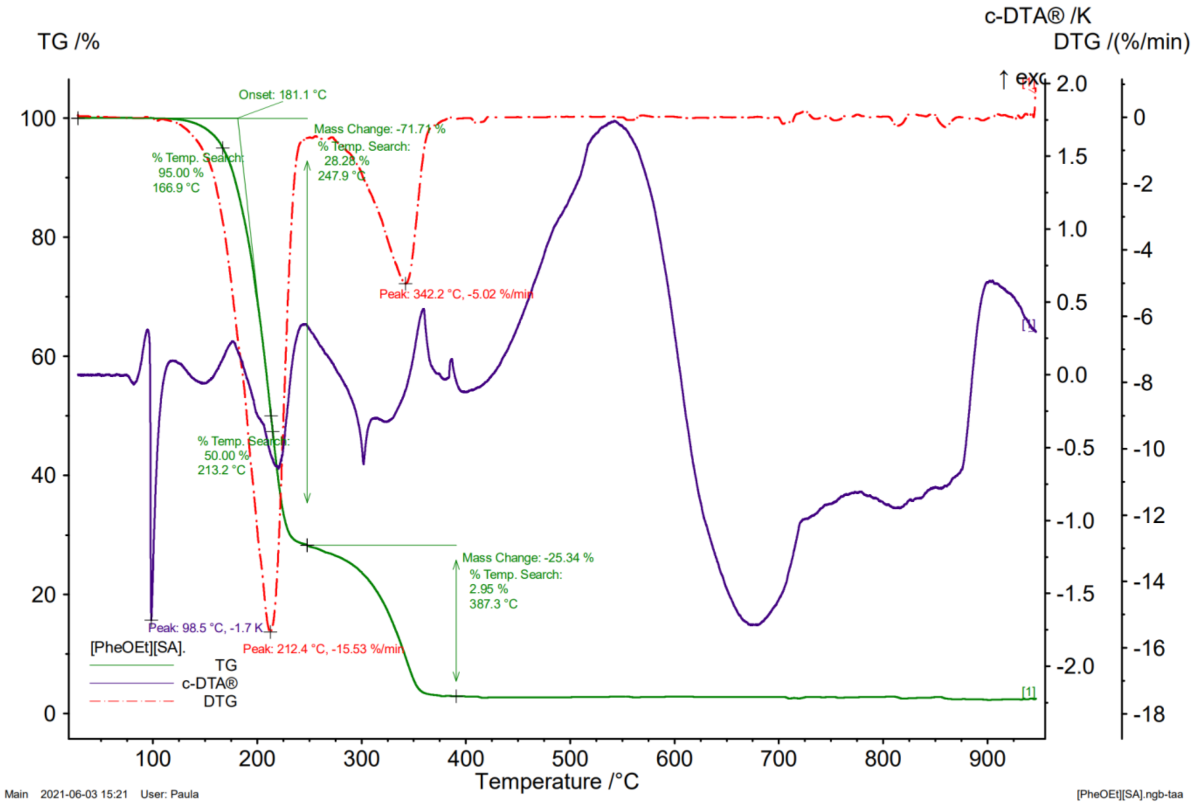

Figure S36. The TG, DTG, and c-DTA curves of [PheOEt][SA].

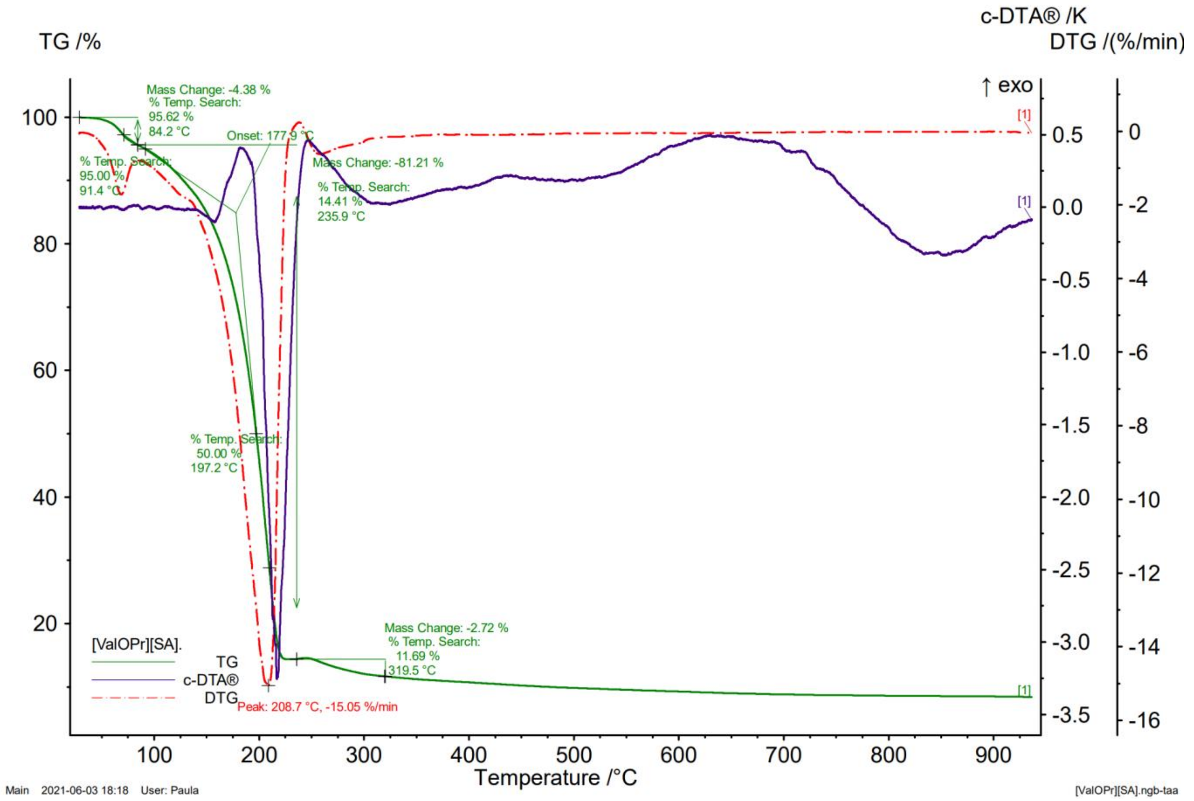

Figure S37. The TG, DTG, and c-DTA curves of [ValOPr][SA].

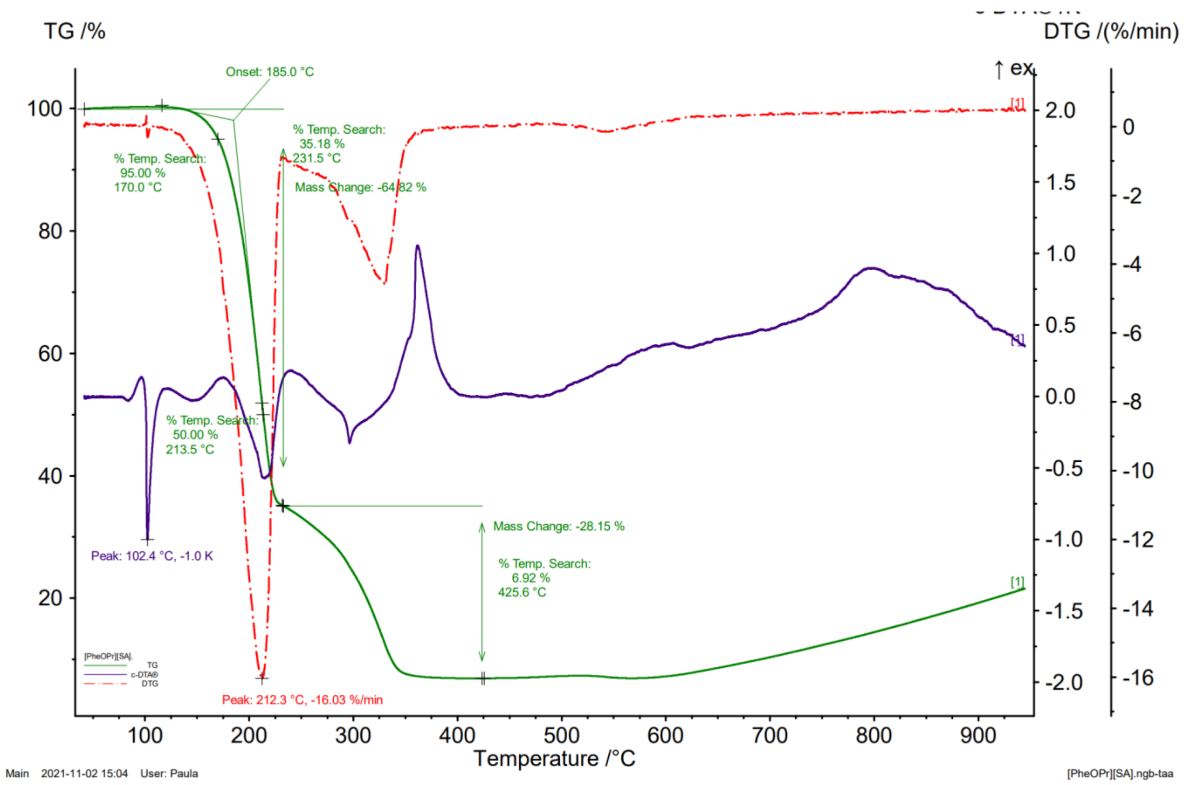

Figure S38. The TG, DTG, and c-DTA curves of [PheOPr][SA].

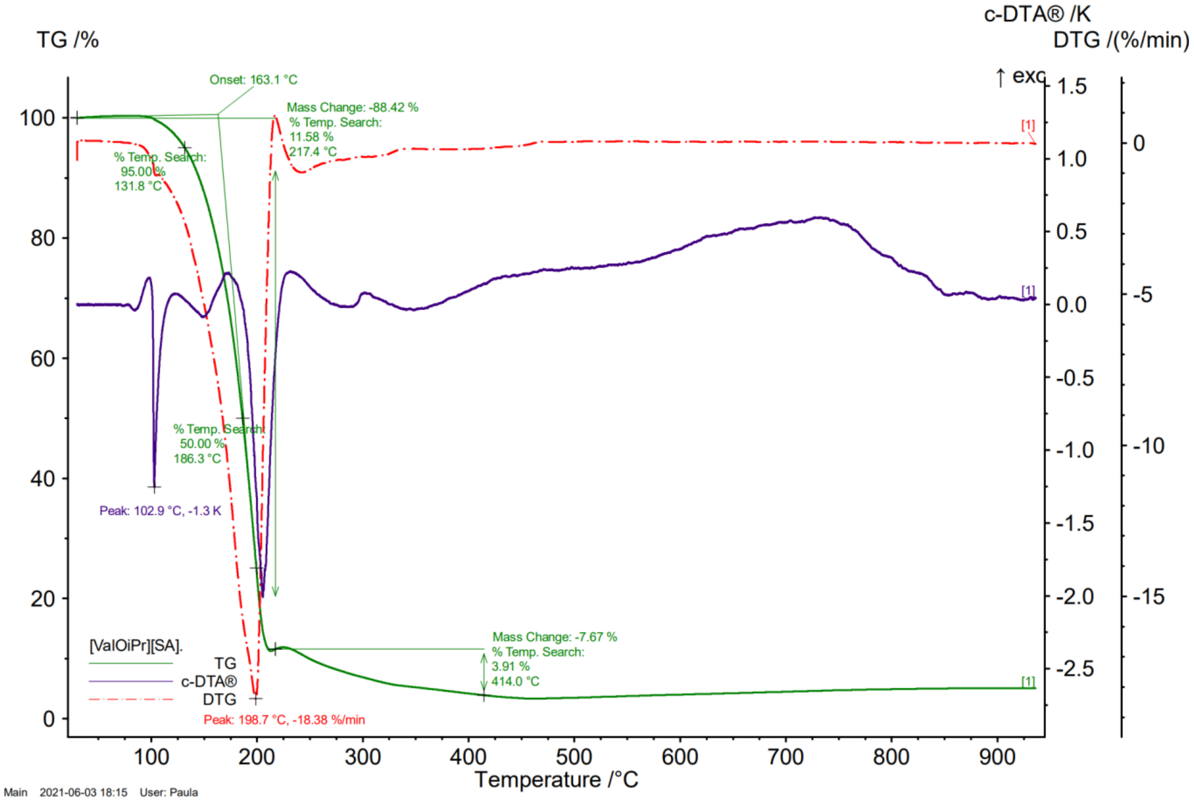

Figure S39. The TG, DTG, and c-DTA curves of [ValOiPr][SA].

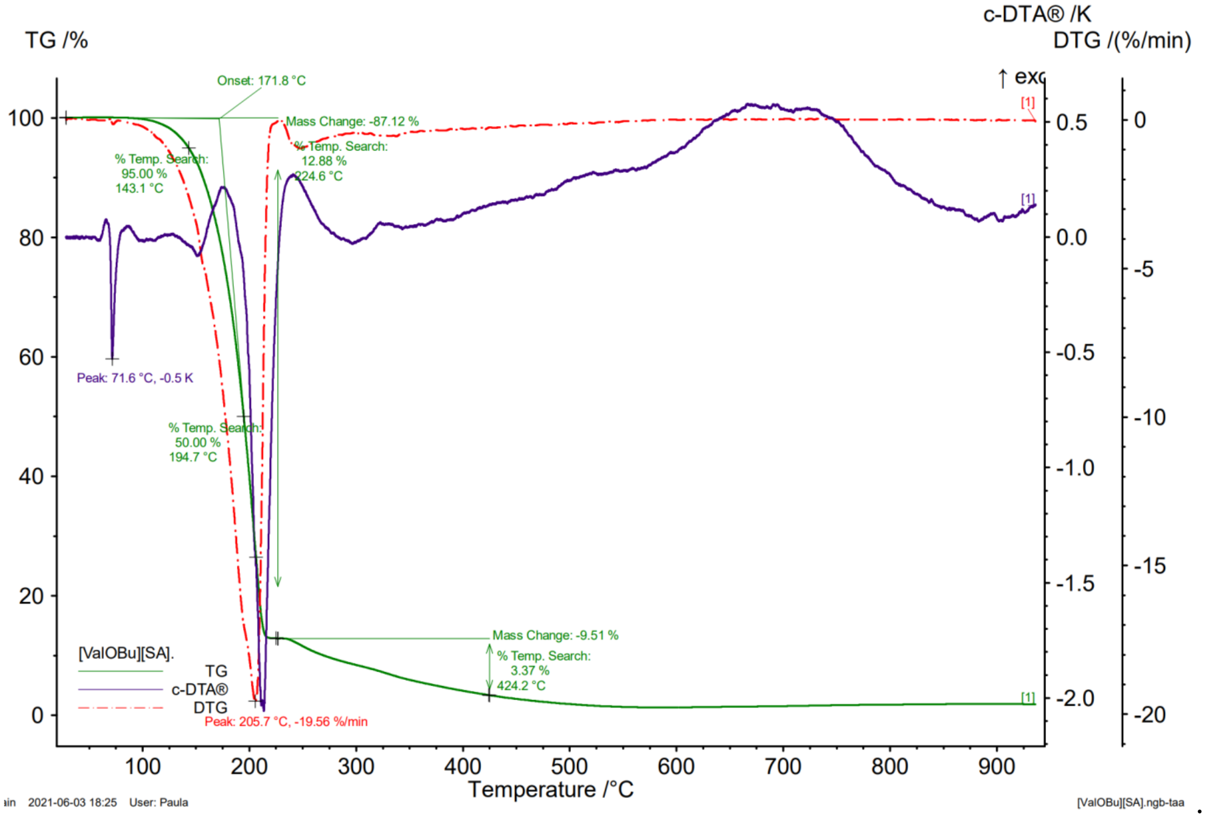

Figure S40. The TG, DTG, and c-DTA curves of [ValOBu][SA].

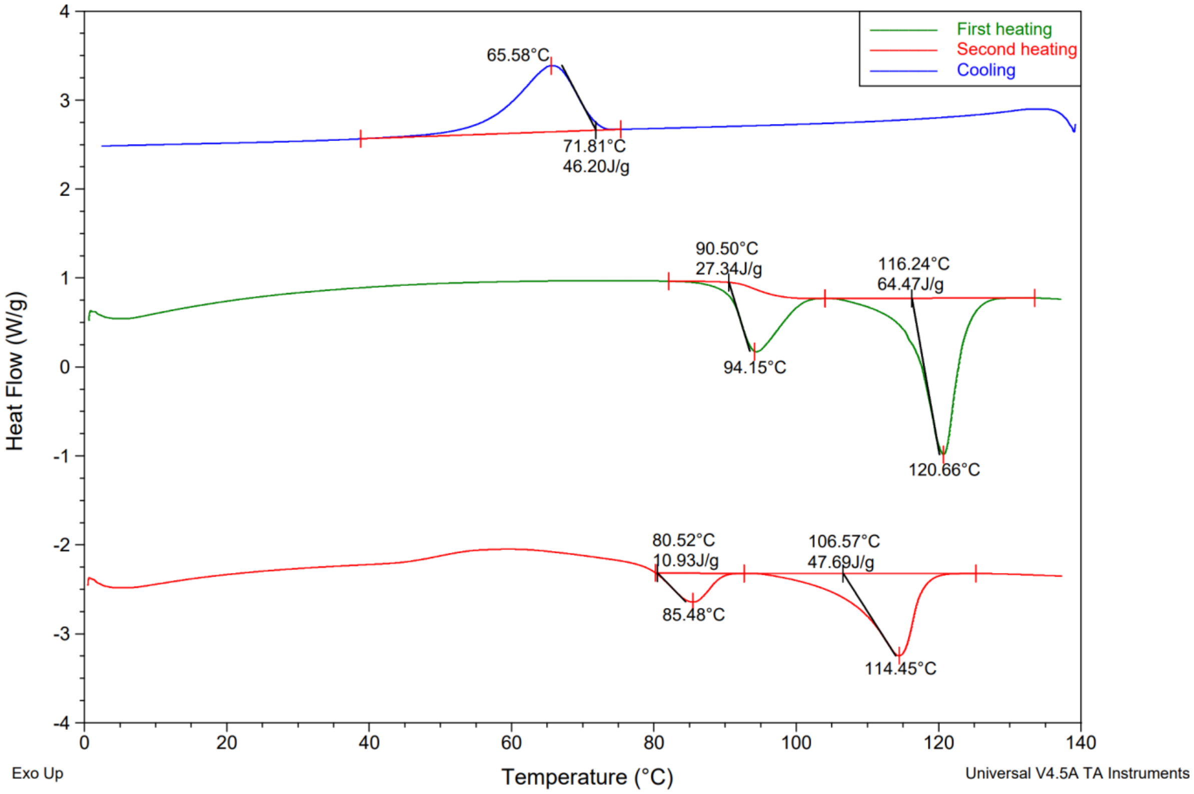

Figure S41. The DSC curves of [PheOMe][SA].

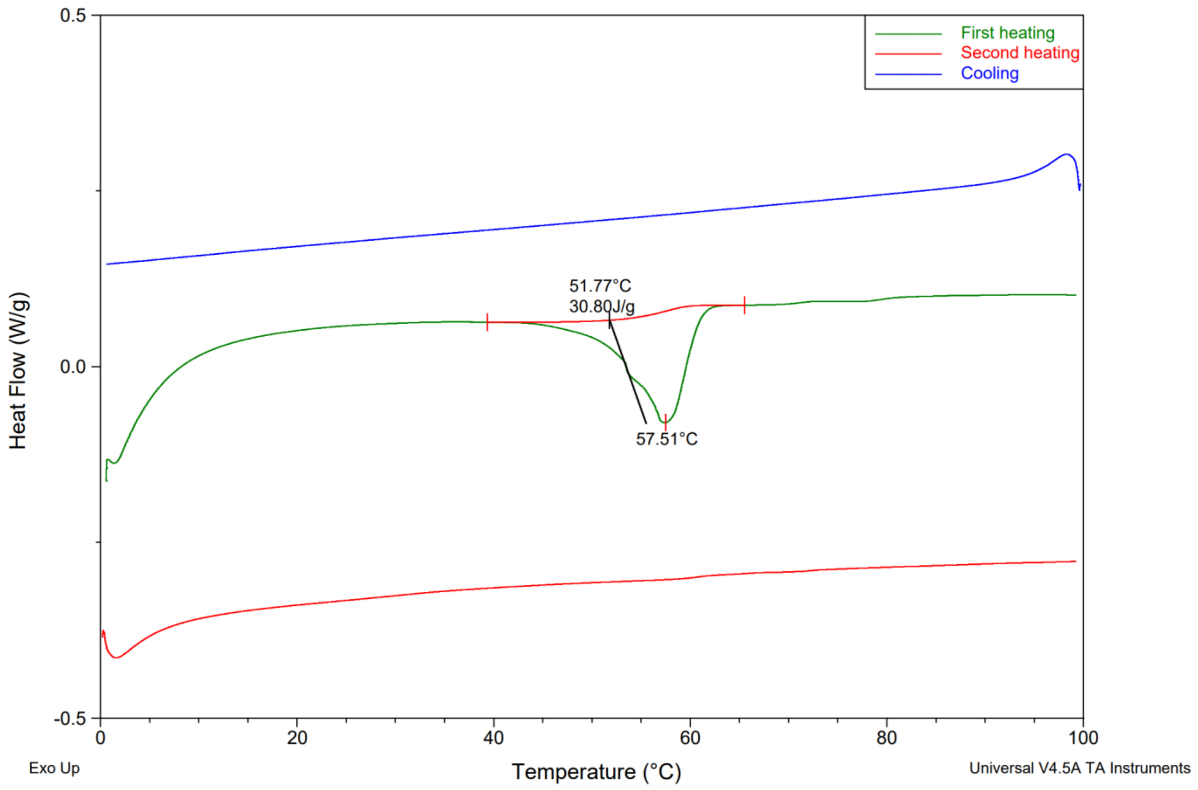

Figure S42. The DSC curves of [ValOEt][SA].

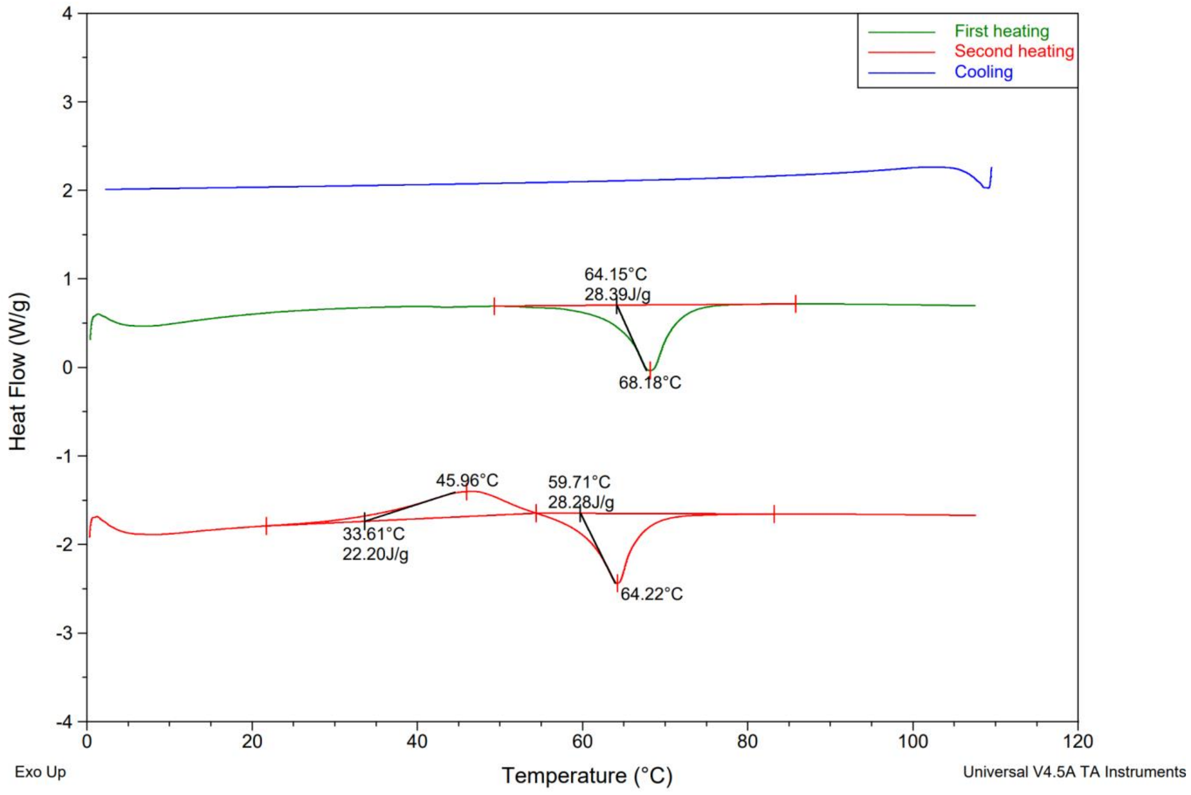

Figure S43. The DSC curves of [LeuOEt][SA].

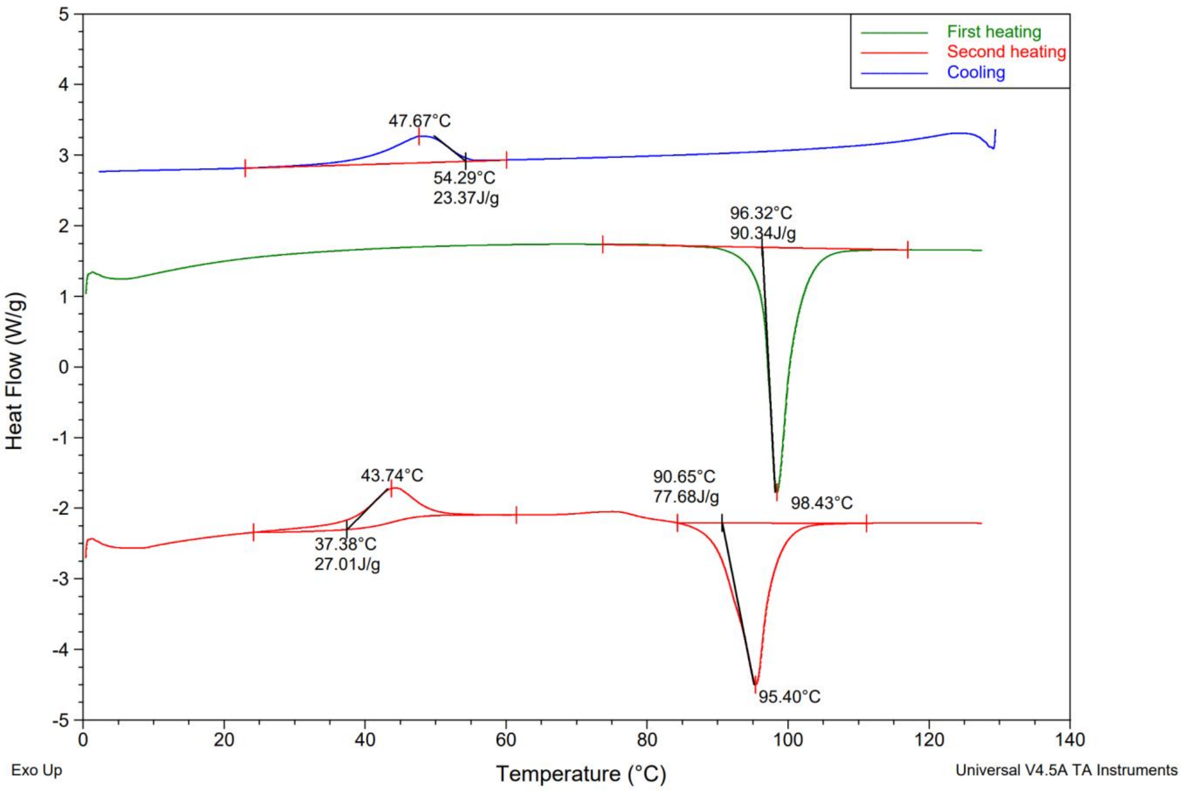

Figure S44. The DSC curves of [PheOEt][SA].

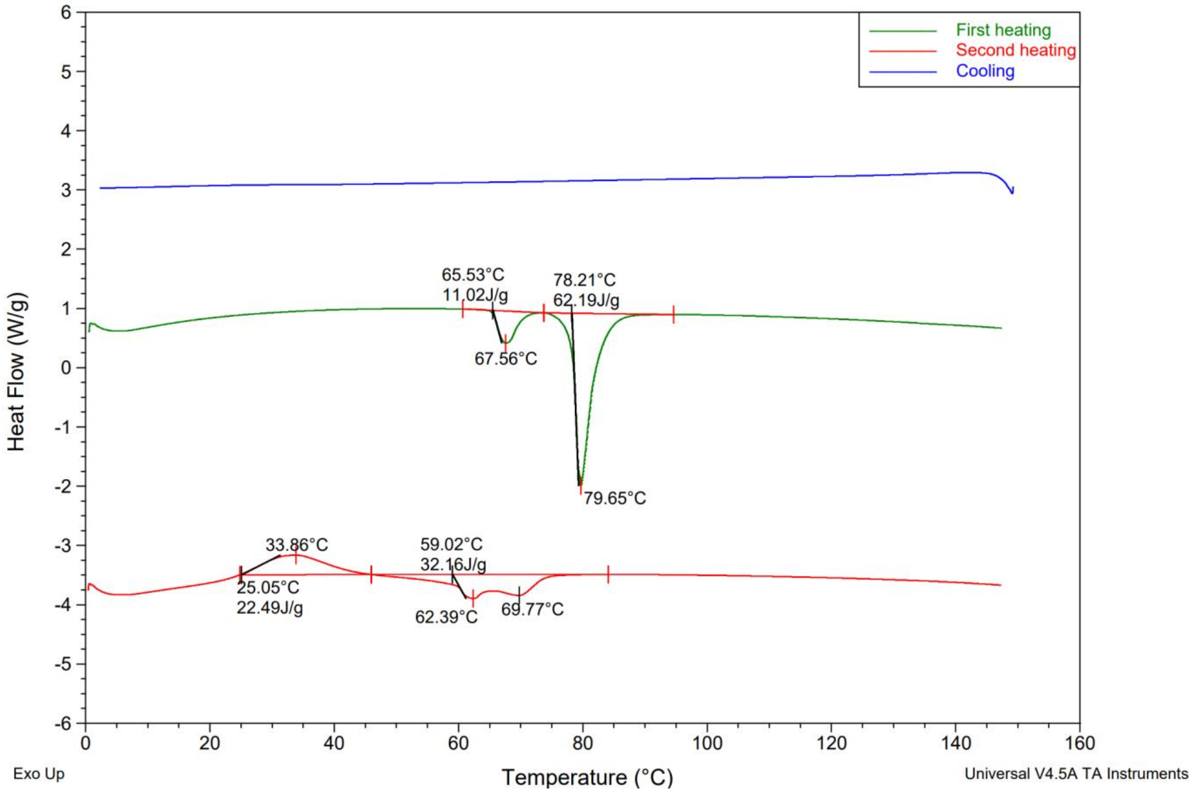

Figure S45. The DSC curves of [ValOPr][SA].

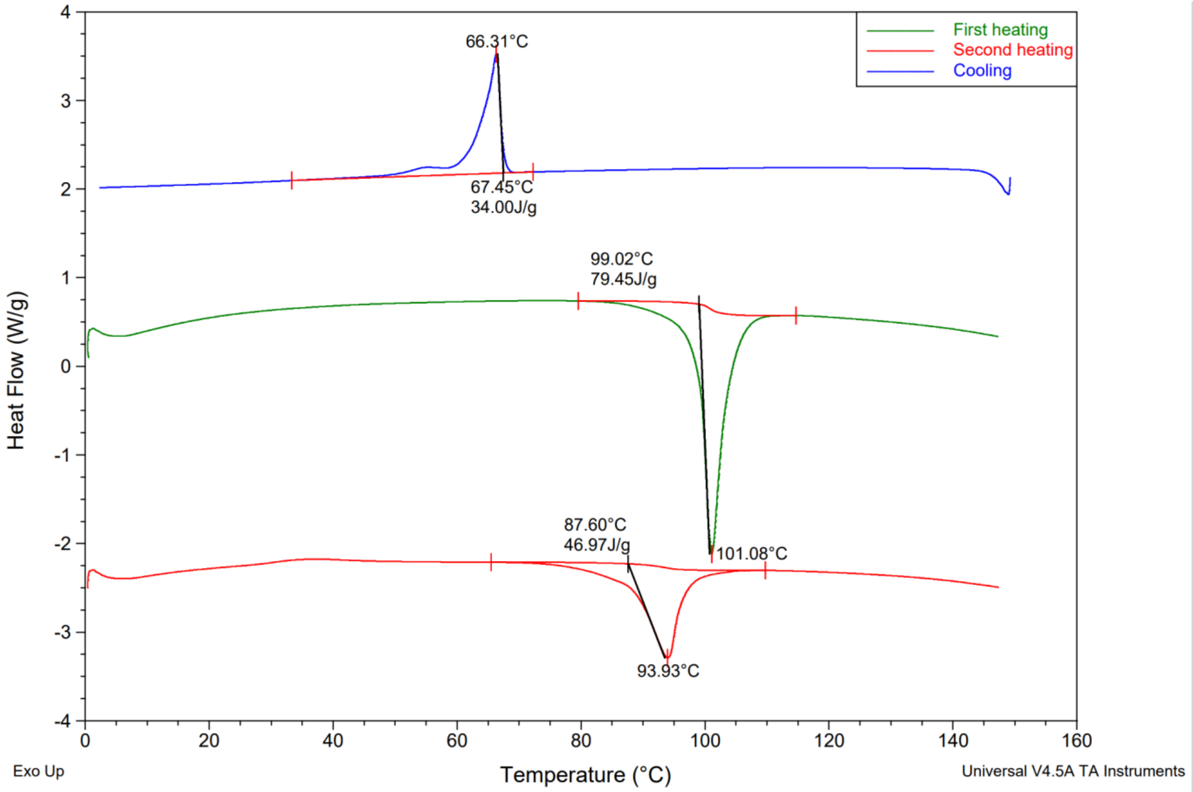

Figure S46. The DSC curves of [PheOPr][SA].

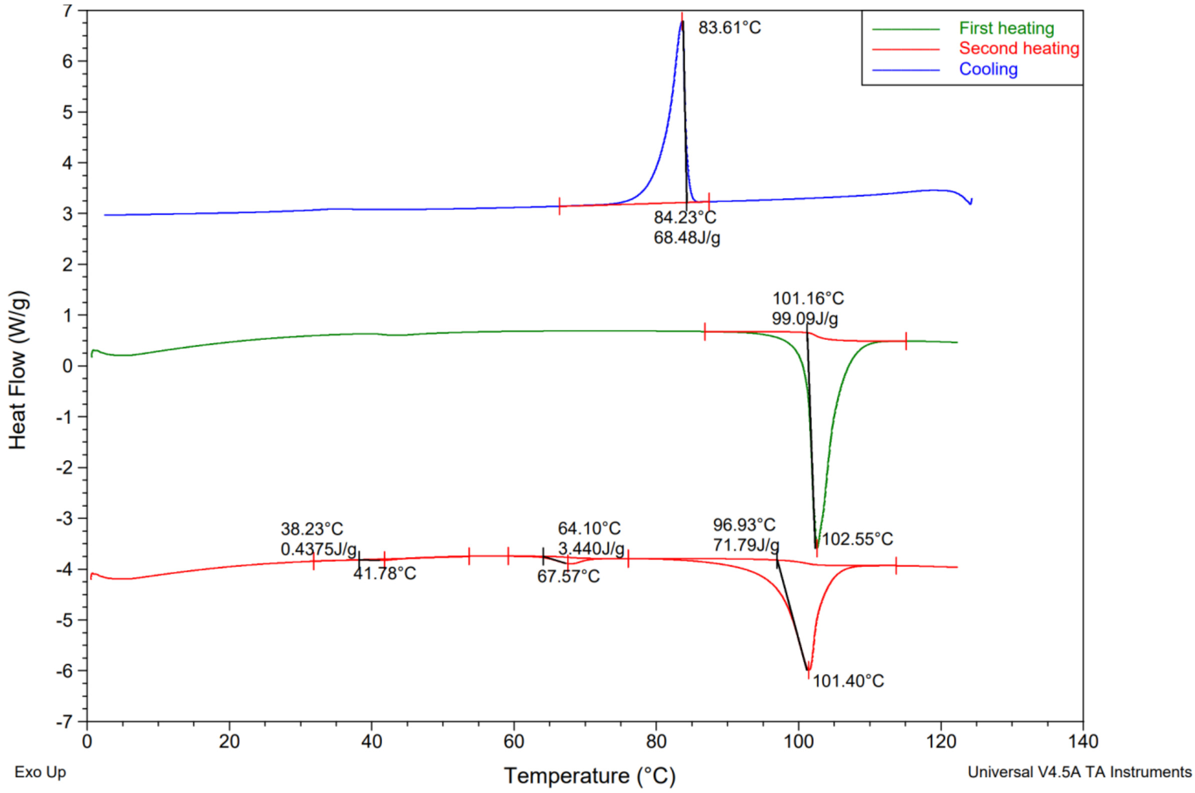

Figure S47. The DSC curves of [ValOiPr][SA].

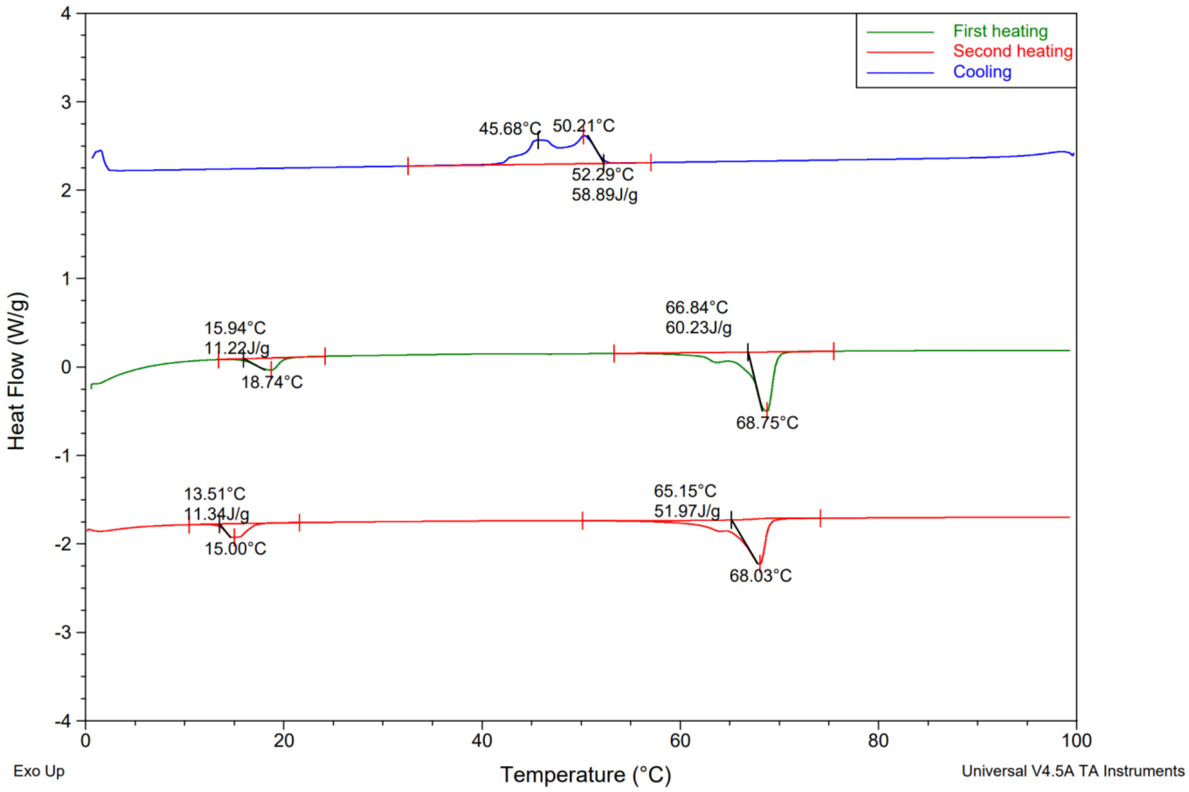

Figure S48. The DSC curves of [ValOBu][SA].

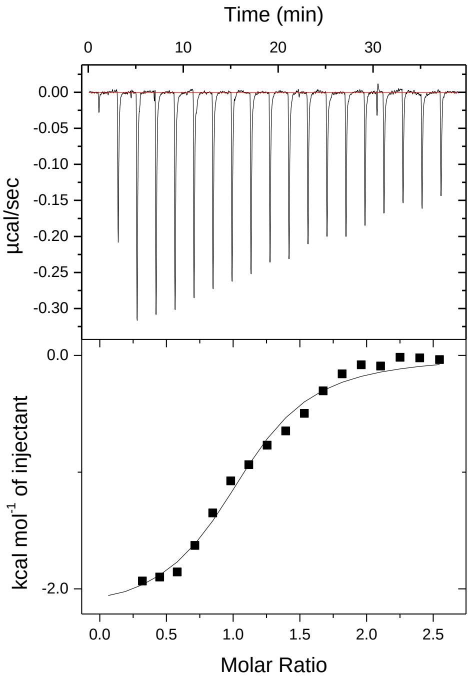

Figure S49. Raw data of titration of 350 µM [LeuOEt][SA] with 27 µM at 25 °C (upper panel). Integrated heat profile of calorimetric titration sown in upper panel (lower panel). The solid lines represents the best fit to a single binding site model.

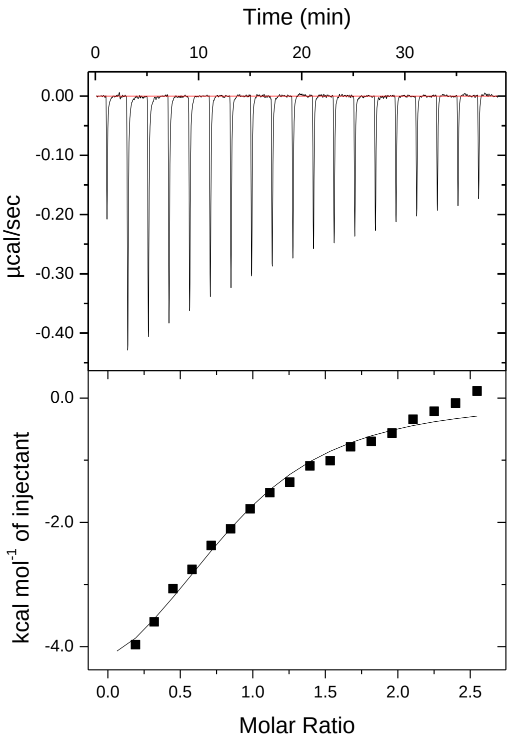

Figure S50. Raw data of titration of 500 µM [PheOPr][SA] with 27 µM at 25 °C (upper panel). Integrated heat profile of calorimetric titration sown in upper panel (lower panel). The solid lines represents the best fit to a single binding site model.

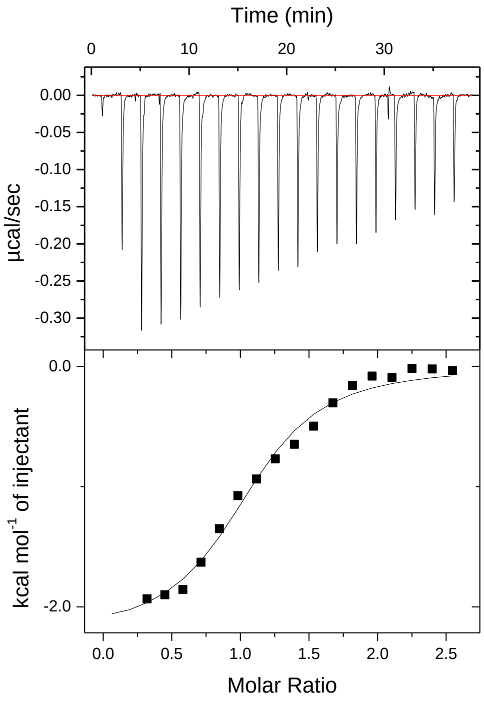

Figure S51. Raw data of titration of 500 µM [PheOPr][SA] with 27 µM at 25 °C (upper panel). Integrated heat profile of calorimetric titration sown in upper panel (lower panel). The solid lines represents the best fit to a single binding site model.

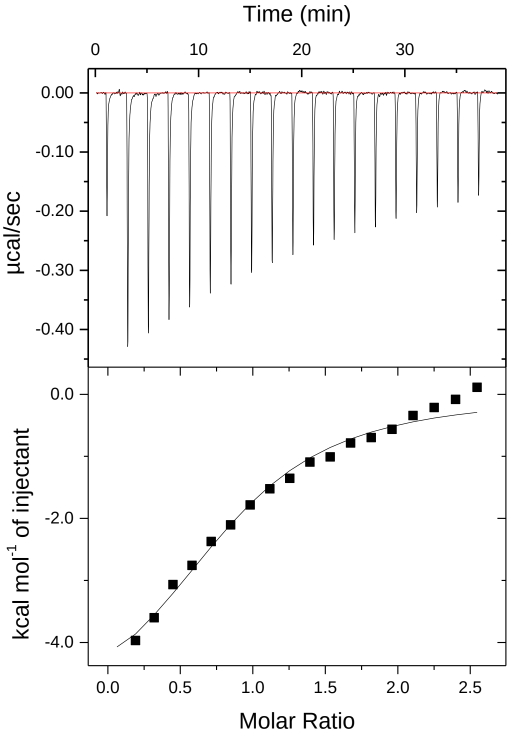

Figure S52. Raw data of titration of 350 µM [ValOiPr][SA] with 27 µM at 25 °C (upper panel). Integrated heat profile of calorimetric titration sown in upper panel (lower panel). The solid lines represents the best fit to a single binding site model.

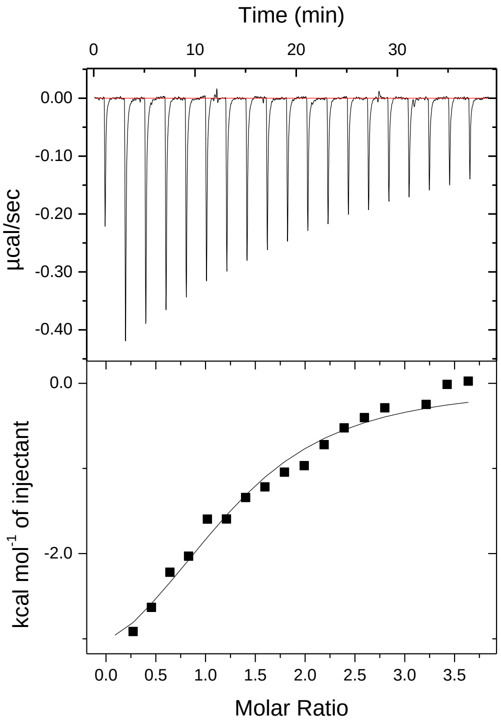

Figure S53. Raw data of titration of 500 µM [ValOBu][SA] with 27 µM at 25 °C (upper panel). Integrated heat profile of calorimetric titration sown in upper panel (lower panel). The solid lines represents the best fit to a single binding site model.

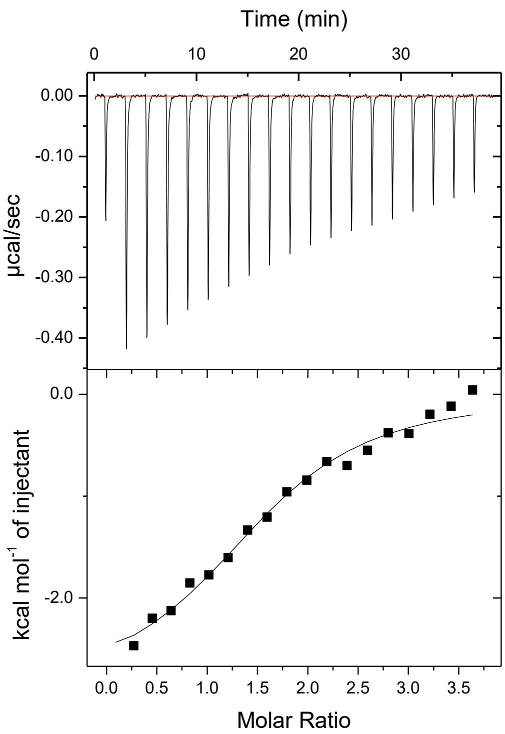

Figure S54. Raw data of titration of 500 µM [ValOPr][SA] with 27 µM at 25 °C (upper panel). Integrated heat profile of calorimetric titration sown in upper panel (lower panel). The solid lines represents the best fit to a single binding site model.

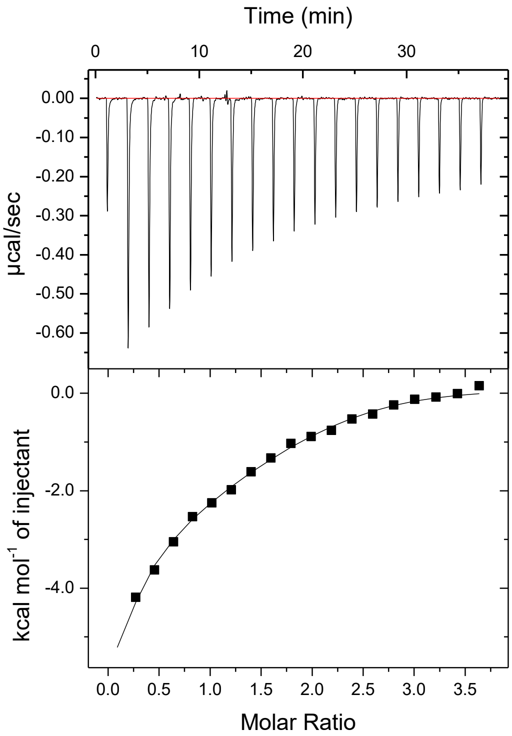

Figure S55. Raw data of titration of 500 µM [ValOEt][SA] with 27 µM at 25 °C (upper panel). Integrated heat profile of calorimetric titration sown in upper panel (lower panel). The solid lines represents the best fit to a single binding site model.

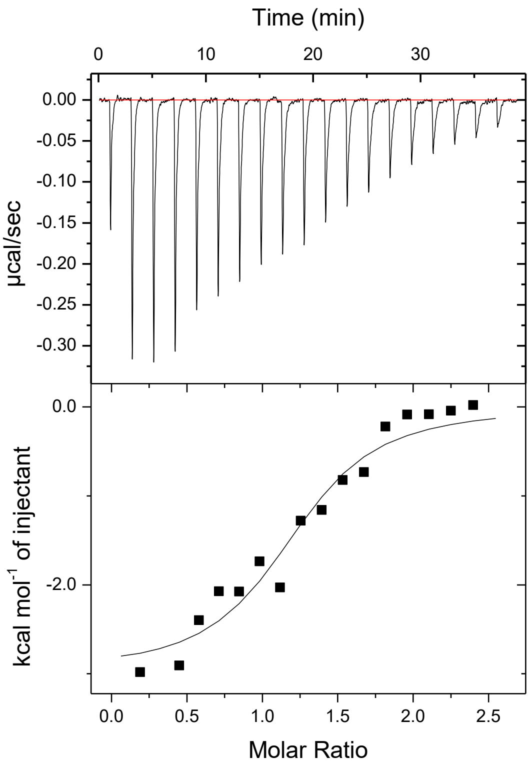

Figure S56. Raw data of titration of 500 µM [PheOEt][SA] with 27 µM at 25 °C (upper panel). Integrated heat profile of calorimetric titration sown in upper panel (lower panel). The solid lines represents the best fit to a single binding site model.

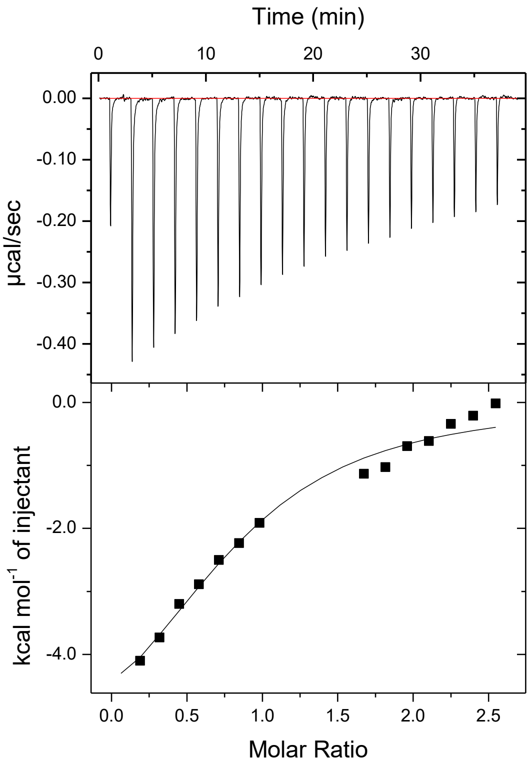

Figure S57. Raw data of titration of 500 µM [PheOMe][SA] with 27 µM at 25 °C (upper panel). Integrated heat profile of calorimetric titration sown in upper panel (lower panel). The solid lines represents the best fit to a single binding site model.
